# Characterization of the Simplest Thiolimine: The Higher Energy Tautomer of Thioformamide

**DOI:** 10.1002/chem.202005188

**Published:** 2021-03-12

**Authors:** Bastian Bernhardt, Friedemann Dressler, André K. Eckhardt, Jonathan Becker, Peter R. Schreiner

**Affiliations:** ^1^ Institute of Organic Chemistry Justus Liebig University Heinrich-Buff-Ring 17 35390 Giessen Germany; ^2^ Institute of Inorganic and Analytical Chemistry Justus Liebig University Heinrich-Buff-Ring 17 35390 Giessen Germany

**Keywords:** conformational interconversion, matrix isolation, prebiotic chemistry, tautomerism, tunneling

## Abstract

As sulfur‐containing organic molecules thioamides and their isomers are conceivable intermediates in prebiotic chemistry, for example, in the formation of amino acids and thiazoles and resemble viable candidates for detection in interstellar media. Here, we report the characterization of parent thioformamide in the solid state via single‐crystal X‐ray diffraction and its photochemical interconversion to its hitherto unreported higher energy tautomer thiolimine in inert argon and dinitrogen matrices. Upon photogeneration, four conformers of thiolimine form, whose ratio depends on the employed wavelength. One of these conformers interconverts due to quantum mechanical tunneling with a half‐life of 30–45 min in both matrix materials at 3 and 20 K. A spontaneous reverse reaction from thiolimine to thioformamide is not observed. To support our experimental findings, we explored the potential energy surface of the system at the AE‐CCSD(T)/aug‐cc‐pCVTZ level of theory and computed tunneling half‐lives with the CVT/SCT approach applying DFT methods.

## Graphical abstract

**Keep it simple**! Thioamides resemble viable prebiotic building blocks for biologically relevant molecules. However, only a handful of experimental reports on parent thioformamide exist. X‐ray diffraction data of thioformamide are reported and its tautomerization induced by UV irradiation under cryogenic conditions is presented. Four conformers of the corresponding thiolimine form, whose (tunneling) reactivity is investigated.

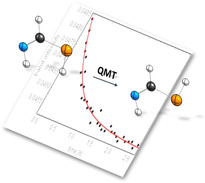

## Introduction

**Scheme 1:**
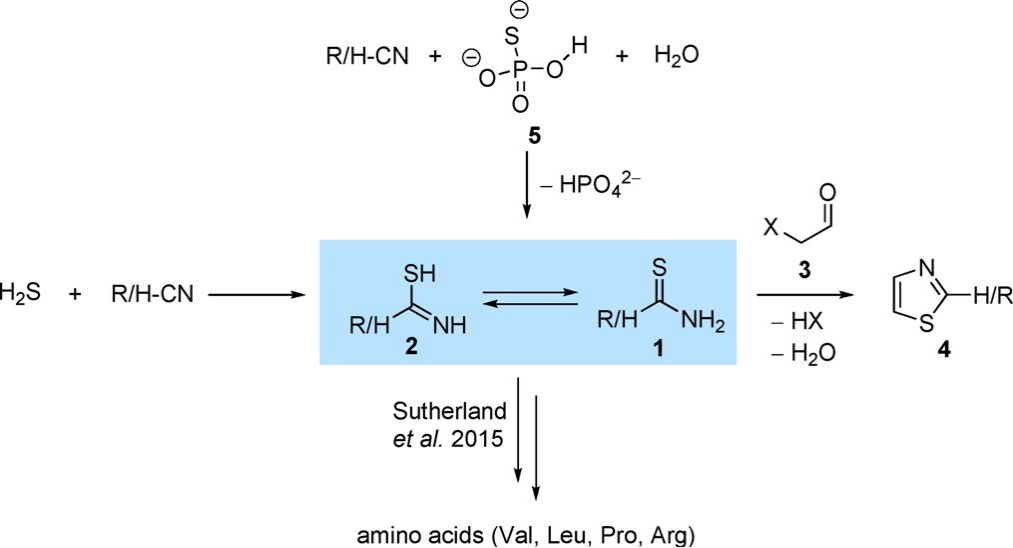
Thioamides are considered key intermediates in the generation of some highly relevant biologically active molecules like amino acids and thiazoles (**4**) in prebiotic context. Their formation from nitriles and H_2_S or thiophosphate (**5**) has been described.^[9]^ Here we focus on parent thioformamide (**1**, R=H) and its tautomerization to **2** (R=H).

Although thioformamide (**1**, methanethioamide, R=H) is the simplest thioamide, it is not as well characterized as one would assume. In nature as well as in synthetic chemistry^[1]^ thioamides resemble building blocks for thiazoles (**4**), a unit that is present in various biologically important molecules (e.g., thiamin) and drugs.^[2]^ General syntheses towards thiazoles build upon reacting thioamides with α‐halogenated aldehydes (**3**).^[3]^ Parent thioformamide is an adduct of HCN and H_2_S,^[4]^ both of which were detected in interstellar media.[^5^, ^6^, ^7^] This makes thioformamide a probable participant in the prebiotic synthesis of sulfur containing compounds important for the development of life (Scheme 1). Accordingly, Sutherland et al. demonstrated that various thioamide derivatives might play a role in the formation of amino acids through what has been termed “cyanosulfidic protometabolism”.^[8]^ In a prebiotic context they can form from the reaction of nitriles with either H_2_S or thiophosphate (**5**).^[9]^ This chemistry displays similarities to that of HCN reacting with H_2_O, which has been investigated computationally recently.^[10]^ The general idea of constructing organic molecules from simple building blocks has been widely accepted since the famous Urey–Miller experiments.^[11]^ Mechanistically, addition of H_2_S to nitriles is expected to proceed via a thiolimine (**2**, methanimidothioic acid, R=H), which is the thermodynamically disfavored tautomer of **1**. To the best of our knowledge, no data exist for **2**, which prompted us to study the tautomerization between **1** and **2** in cryogenic argon (Ar) and dinitrogen (N_2_) matrices to provide first‐hand evidence for the existence and spectral identity of **2**. We also report the first single‐crystal X‐ray diffraction structure of **1**.

Various thioamide derivatives have been isolated under cryogenic conditions and characterized by infrared (IR) spectroscopy during the last decades. Several studies focused on the UV‐induced thioamide→thiolimine tautomerization, which has been reported for 2(1*H*)‐pyridinethione,^[12]^ 4(3*H*)‐pyrimidinethione and 3(2*H*)‐pyridazinethione,^[13]^ 2(1*H*)‐quinolinethione,^[14]^ methimazole,^[15]^ 2‐thiobenzimidazole,^[16]^ thiourea,[^17^, ^18^] and thioacetamide.[^19^, ^20^, ^21^] In the case of thiourea the reverse thiolimine → thioamide reaction has been observed even under cryogenic conditions and the results were interpreted as to involve quantum mechanical tunneling (QMT).[^17^, ^18^] Dithiooxamide shows UV‐induced double proton transfer forming the dithiolimine and the reverse reaction due to QMT.^[22]^ Thioacetamide, on the other hand, does not display such a tunneling reaction under experimental laboratory conditions.[^19^, ^20^] Generally speaking, QMT is an abundant, yet often underappreciated phenomenon in chemical reactions, and it is especially well observable under cryogenic matrix isolation conditions when first order tunneling half‐lives range from a few seconds to several days.[^23^, ^24^, ^25^, ^26^] Due to the similarities regarding cryogenic temperatures and very low concentrations, many of such reactions are also highly relevant in interstellar media.

Owing to its quick decomposition at ambient conditions (see Supporting Information) reports on experimental data of **1** are rare.[^27^, ^28^] Only two infrared studies on **1** in the liquid and solid phases exist, which provide IR spectra in good agreement with each other, but are controversial in the assignment of the observed bands.[^29^, ^30^] However, no literature exists on the (photo‐)reactivity of **1**. In stark contrast, the amide→enolimine interconversion of the congener formamide induced by irradiation with 248 nm in an Ar matrix at 10 K has been known since 2000^[31]^ and its matrix IR spectra as early as 1970.[^32^, ^33^] Irradiation with wavelengths >160 nm leads to its photodecomposition into complexes of HCN, HNC, HNCO, and H_2_O.^[34]^ The tautomerizations of selenoformamide^[35]^ and telluroformamide have been investigated only theoretically.^[36]^ For all homologues this process has also been studied computationally by modelling aqueous hydration shells, which yielded significantly lower proton transfer barriers compared to the unimolecular gas phase reaction.^[37]^

The mechanism of the UV‐induced tautomerization of thioamides has been discussed. Excited state intramolecular proton transfer (ESIPT) has been ruled out by Lapinski et al. due to the lack of intramolecular hydrogen bonds in these systems.^[19]^ For methimazole^[15]^ and 2‐thiobenzimidazole^[16]^ Brás and Fausto considered a photoinduced hydrogen‐atom detachment‐association (PIDA) mechanism, which has been described theoretically by Sobolewski et al.[^38^, ^39^, ^40^, ^41^] Eventually, a study combining Raman spectroscopy and theory reconsidered the possibility of ESIPT to play a role in the tautomerization mechanism in thioacetamide.^[42]^

## Results and Discussion

### Computations and X‐ray diffraction data

**Scheme 2:**
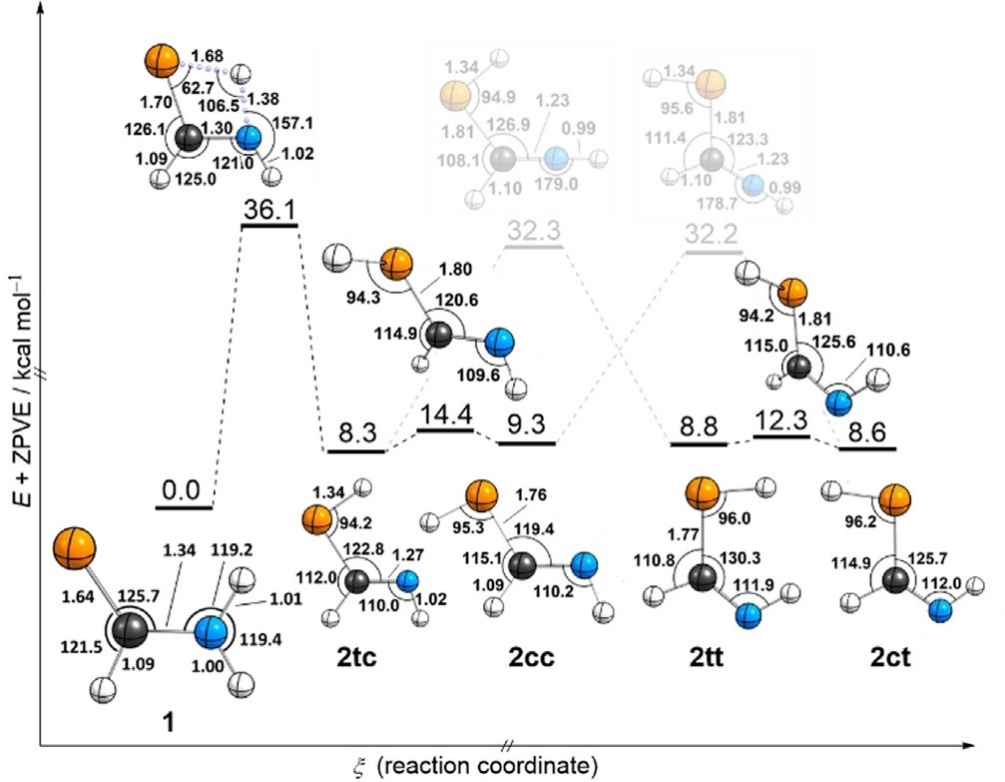
Potential energy surface of thioformamide (**1**) and its thiolimine tautomers (**2**) computed at the AE‐CCSD(T)/aug‐cc‐pCVTZ level of theory. In our nomenclature for **2** the first letter represents *s‐cis* or *s‐trans* orientation of the S−H bond while the second letter represents *s‐cis* or *s‐trans* orientation of the N−H bond. Bond lengths are given in Å and angles in degrees of geometries optimized at the level of theory mentioned above. Only those internal coordinates that change throughout the displayed reactions are depicted, for exhaustive geometric data see the Supporting Information. Color code: Carbon—black, hydrogen—white, nitrogen—blue, sulfur—yellow.

We computed the most significant part of the HC(S)NH_2_ potential energy surface with density functional theory (DFT; see Supporting Information for details) as well as with coupled cluster methods (Scheme 2). All computed minima shown in Scheme 2 display *C*_s_ symmetry at all levels of theory applied in this study. According to the relative energies the ground state structure at cryogenic temperatures is the thioamide tautomer **1**, which is more than 8 kcal mol^−1^ lower in energy than its thiolimine tautomers **2**. There are four conceivable conformers of **2** that are very close (within 1 kcal mol^−1^) in energy. The order of stability is **2 cc**<**2 tt**<**2 ct**<**2 tc** at every computational level applied here. The conformers can interconvert through C−S bond rotations or inversion of the C=N−H angle. The latter requires overcoming high lying *C*_s_ symmetric transition states.

**Figure 1:**
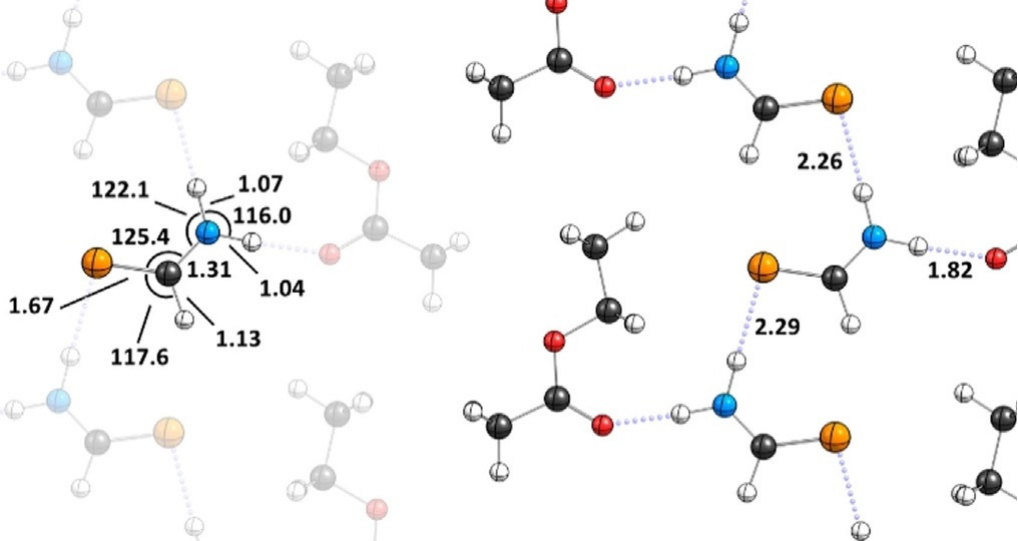
Slice through a crystal containing **1** and ethyl acetate. Left: Geometric details of a thioformamide molecule embedded in the crystal. Right: Intramolecular interactions between molecules of **1** with each other and ethyl acetate. Lengths are given in Å and angles in degrees. Color code: carbon—black, hydrogen—white, nitrogen—blue, oxygen—red, sulfur—yellow.

We obtained single crystals of **1** in ethyl acetate and solved their structure by X‐ray diffraction and refined the data with the independent atom model and Hirshfeld atom refinement (Figure 1).^[43]^ The excellent agreement of the computed geometry of **1** (Scheme 2) with the X‐ray structure lends credibility to the theoretical approach. Within the crystal molecules of **1** are aligned in an array between ethyl acetate clusters. There are hydrogen bonding contacts within these arrays, but also between **1** and ethyl acetate. The structure of a single crystal of pure formamide has already been reported in 1954.^[44]^ Formamide forms *C*_2*h*_ symmetric dimers bounded with two N−H⋅⋅⋅O contacts. Gas‐phase B3LYP/6–311++G(3df,3pd) computations predict that the analogous *C*_2*h*_ dimer of **1** containing two N−H⋅⋅⋅S contacts is 2.0 kcal mol^−1^ lower in energy than a *C*_s_ dimer as depicted in Figure 1 containing only one such interaction. Note that the C−H⋅⋅⋅S contact in the crystal is 2.64 Å and, hence, cannot be accounted for a relevant binding contact.^[45]^ The strong interaction between **1** and ethyl acetate (N−H⋅⋅⋅O contact of only 1.82 Å) is presumably the reason for the difference from the crystal structure in Figure 1 from the one of formamide and the computations. In a different vein, analogous S=C−N−H⋅⋅⋅O=C interactions resemble the central motif in thiourea catalysis.[^46^, ^47^]

### Matrix isolation and characterization of thioformamide

**Figure 2:**
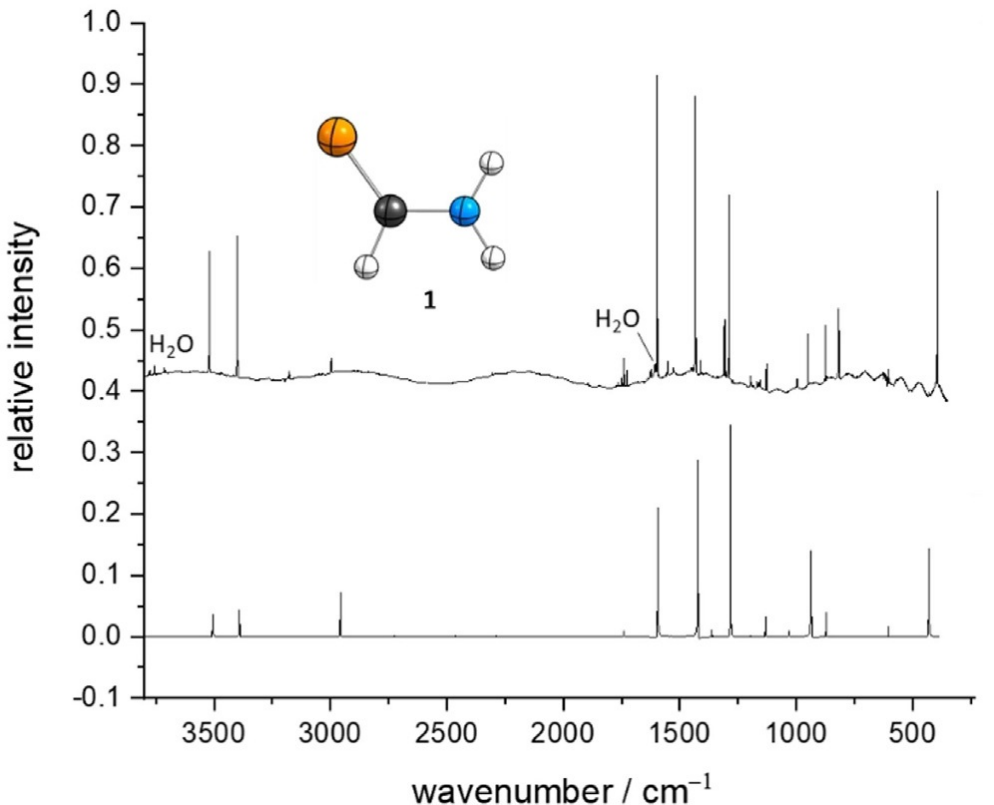
Top: IR spectrum of thioformamide (**1**) after deposition in an Ar matrix at 3 K. Bottom: Computed anharmonic IR spectrum of **1** at B3LYP/6–311++G(3df,3pd).

After deposition of a sample of freshly synthesized thioformamide (see Supporting Information for details) together with a large excess of Ar on the cold (3 K) matrix window, we measured IR and UV/Vis spectra of all matrix isolated species. The IR spectrum displayed in Figure 2 shows excellent agreement with the anharmonic spectrum computed at B3LYP/6–311++G(3df,3pd). Using Ar as the host material, the strongest bands are observed at 3519.4 (calc. 3507.2), 3402.2 (3391.1), 1597.6 (1596.7), 1432.0 (1424.2), 1287.6 (1283.3), and 393.1 (432.3) cm^−1^. We also recorded an IR spectrum of **1** in an N_2_ matrix. For the complete assignments see the Supporting Information. Our results are in good agreement with the experimental data by Davies and Jones^[29]^ and Suzuki.^[30]^ Additionally, there is a good match with a computational study by Kowal.^[48]^ Our assignment is based on this latter study, which is in general accord with the one provided by Suzuki and the computations conducted herein.

**Figure 3:**
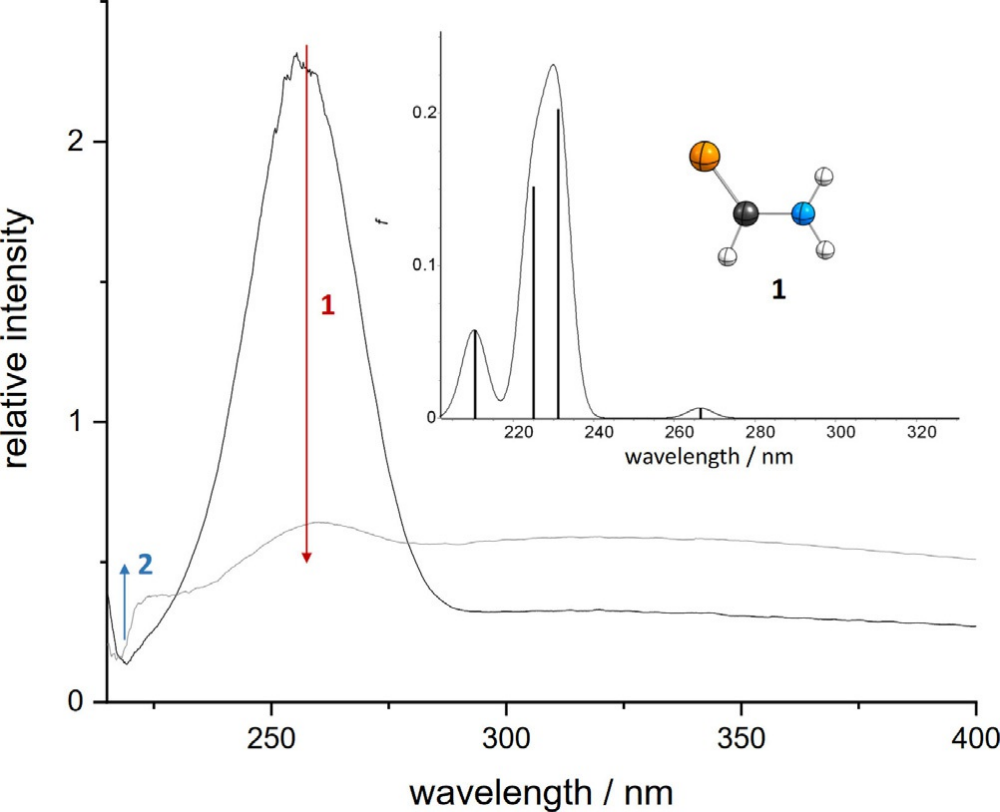
UV/Vis spectrum of the Ar matrix containing **1**. Black: After deposition of **1** in an Ar matrix. Grey: After irradiation with 254 nm for 10 min. Inset: Computed spectrum of **1** at TD‐B3LYP/6–311++G(3df,3pd).

The recorded matrix UV/Vis spectrum (Figure 3) shows a strong absorption at ca. 260 nm. TD‐B3LYP/6–311++G(3df,3pd) computations for thioformamide suggests two strong absorptions at 231 nm (*f*=0.2027) and 225 nm (*f*=0.1517 nm). After UV irradiation (vide infra) a small new feature at ca. 225 nm appears. This is in accord with the computational result that all thiolimine tautomers show absorptions between 210 nm and 215 nm (0.05<*f*<0.15). For an analogous spectrum obtained in N_2_ matrices as well as details of the nature of the transitions see the Supporting Information.

### UV‐induced thioamide to thiolimine interconversion

Irradiation of the matrix with UV light led to a decrease of the thioformamide bands. New bands appear in the IR spectra that can be assigned to **2**. The four conceivable conformers of this species can all be observed in these spectra. For ease of identification, we computed anharmonic IR spectra of **1** and **2** at B3LYP/6–311++G(3df,3pd). DFT in conjunction with a large Pople basis set generally reproduces matrix IR spectra very reasonably; recent precedence is provided in a study on thiotropolone.^[49]^ Herein, the inclusion of anharmonicities leads to an overall good agreement between computations and measurements (see Supporting Information for details).

**Figure 4:**
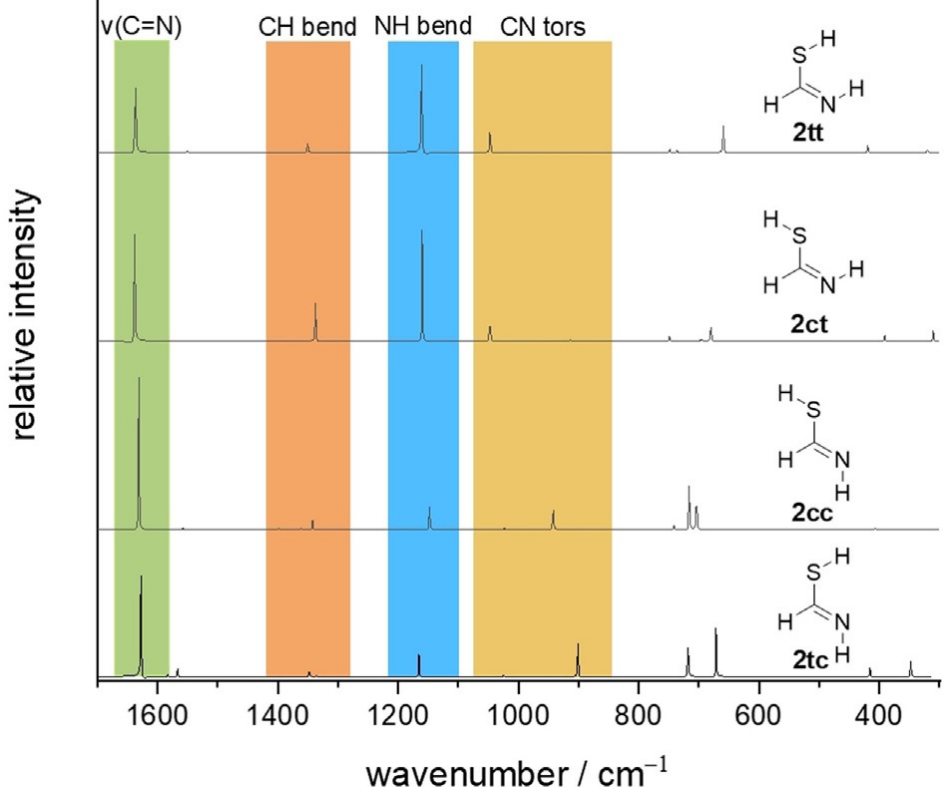
Computed anharmonic IR spectra of the four conceivable conformers of thiolimine **2** at B3LYP/6–311++G(3df,3pd).

The computed intensities of the N−H and the S−H stretching vibrations of **2** are very low. The remainder of the simulated spectra is similar for all four conformers. The intensity pattern, especially in the C−H and N−H bending regions, turns out to be very useful to distinguish between the two groups **2 tc**/**2 cc** and **2 ct**/**2 tt**. In the CN torsion region, the bands of **2 ct** and **2 tt** have nearly the same computed value. On the other hand, **2 tc** and **2 cc** display large shifts, such that this region is the most prominent for assignment (Figure 4). According to the anharmonic computations, only the CN torsion region in the IR spectrum differs significantly for the four conformers: two bands at 940.0 and 912.7 cm^−1^ appear after UV irradiation and only **2 tc** and **2 cc** are computed to show IR absorptions. We assign the first band to **2 cc** (calc. 944.2 cm^−1^) and the second to **2 tc** (calc. 902.5 cm^−1^). The band at 1059 cm^−1^ only has a computed counterpart in conformers **2 ct** and **2 tt**. As discussed below both conformers are present in the matrix and their IR bands can only be resolved in difference spectra (1058.9 and 1059.9 cm^−1^, see Figure 6). The computed values are 1048.8 and 1048.3 cm^−1^ for **2 ct** and **2 tt**, respectively.

**Figure 5:**
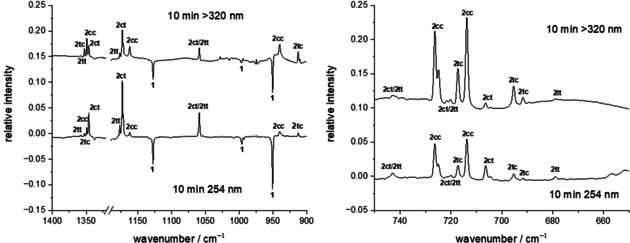
IR difference spectra of spectra measured before and after UV irradiation of the matrix. The ratio of the groups **2 tc**/**2 cc** and **2 ct**/**2 tt** is sensitive to the applied wavelength allowing for band assignment throughout the displayed spectral regions.

A study by Lapinski et al. demonstrated that the thioamide to thiolimine interconversion of thioacetamide yields different ratios of thiolimine conformers when the matrix is irradiated at different wavelengths.^[19]^ Following this strategy we performed one experiment using 254 nm and another in which only wavelengths >320 nm could pass through a cut‐off filter installed in front of the matrix window. The difference spectra of the spectra recorded before and after 10 min of irradiation are presented in Figure 5. The intensity ratio of the new bands is indeed sensitive to the applied wavelength. At lower energies (>320 nm) **2 tc** and **2 cc** are the main products after UV excitation while **2 ct** and **2 tt** are more easily accessible at lower wavelengths. This can be rationalized by taking into account the higher activation barriers for bending the N=C−S angle compared to rotating around the C−S bond (Scheme 2). This finding supports our assignment in the CN torsion region and allows to assign other bands to the groups **2 tc**/**2 cc** and **2 ct**/**2 tt**. Intensity patterns, comparing experimental with computational shifts, and interconversion of the conformers (see below) serve to resolve these two groups further and enables a confident assignment of each observed band to one conformer.

In the CH stretching region at around 1350 cm^−1^ and the NH bending region at around 1170 cm^−1^ four new bands appear after UV irradiation. Taking the described strategy into account we assign the strongest of these bands at 1346.2 cm^−1^ to **2 ct** (calc. 1338.4 cm^−1^). With the same strategy the band at 1349.3 cm^−1^ is assigned to **2 cc** (calc. 1343.8 cm^−1^), the band at 1352.7 cm^−1^ to **2 tc** (calc. 1349.1 cm^−1^), and the band at 1358.0 cm^−1^ to **2 tt** (calc. 1352.2 cm^−1^). The different shifts and intensities throughout the spectrum allow us to draw the conclusion that indeed **2 ct** and **2 tt** are both present. In the N−H bending region the band at 1162.2 cm^−1^ can be assigned to **2 cc**, the strong bands at 1171.8 cm^−1^ and 1173.2 cm^−1^ to **2 ct**, and the band at 1176.9 cm^−1^ to **2 tt**. The computed values are 1149.7, 1161.3, and 1161.9 cm^−1^ for **2 cc**, **2 ct**, and **2 tt**, respectively, which reproduce the experimental spectra in good agreement.

Our assignment is further supported by the observation of a tunneling process from **2 tt** to **2 ct** and the rotamerization of **2 tc** to **2 cc** (vide infra). In the 670–750 cm^−1^ range, all conformers are computed to show several characteristic bands as well. The assignment is possible by combining the above strategies. Analogously, we observed all four thiolimine conformers in N_2_ matrices. Spectra and assignments are available in the Supporting Information.

After prolonged UV irradiation the new bands assigned to the thiolimine tautomers vanished. The strongest bands after irradiation for 1 h are at 3361.8, 3202.3, 2091.2, 2026.2, 1233.4, 647.2, and 638.9 cm^−1^. These bands cannot be confidently assigned to the decomposition products H_2_S, HCN, HNC, CS, NH_3_, H_2_, HSCN, or HCNS because in the present mixture significant shifts of the predicted bands of the pure compounds might occur due to the formation of complexes. Bands at 1624.1, 1553.0, and 1408.1 cm^−1^ remain unchanged during UV irradiation and can thus be assigned to unknown impurities in our sample. The highest concentration of the thiolimine tautomers is observed after 10 min of irradiation when **1** is still among the main components.

### Quantum mechanical tunneling (QMT)

**Figure 6:**
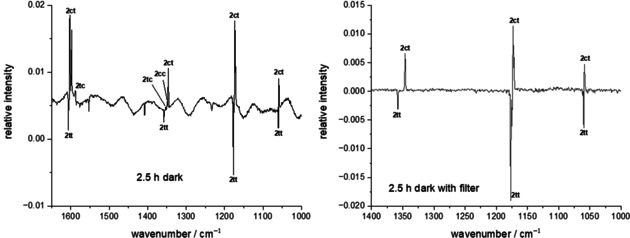
IR difference spectra of spectra measured before and after recording the kinetics for 2.5 h. Usage of a 4.5 μm cut‐off filter prevented the reaction **2 tc→2 cc** induced by the Globar of the spectrometer while **2 tt**→**2 ct** proceeds due to QMT.

After keeping the matrix for 2.5 h in the dark we observed a decay of bands belonging to **2 tt** and a concomitant increase of those assigned to **2 ct**. We measured the kinetics of this process by collecting IR spectra in 5 min intervals. Figure 6 shows that IR measurements without the use of a 4.5 μm (≙2222 cm^−1^) cut‐off filter leads to the formation of **2 cc** from **2 tc**. This can be rationalized by the low activation barrier of only 6.1 kcal mol^−1^. We notice that wavelengths >4.5 μm (≙6.4 kcal mol^−1^) would in principle suffice to initiate this process, however, we observe that when using such a cut‐off filter only the reaction **2 tt**→**2 ct** occurs. It is important to stress that even after longer times (up to 48 h) the intensities of bands of **2 tt** do not reach zero. This might on the one hand be due to the IR Globar irradiation from the spectrometer (the activation barrier for the reverse reaction **2 ct**→**2 tt** is only 3.7 kcal mol^−1^). On the other hand, such phenomena have been assigned to matrix effects before, namely that specific matrix environments (or matrix sites) inhibit QMT.^[50]^ Recent precedence is provided in a study on thioacetamide.^[20]^ Furthermore, for the *N*,*N*‐dideuterated isotopologue of thioformamide dark reactions of the thiolimine tautomers were not observed when installing the cut‐off filter. This provides another hint that the reaction **2 tt**→**2 ct** is due to QMT and resembles only the second example of conformational QMT leading to a rotation of a C−S bond.^[20]^

**Figure 7:**
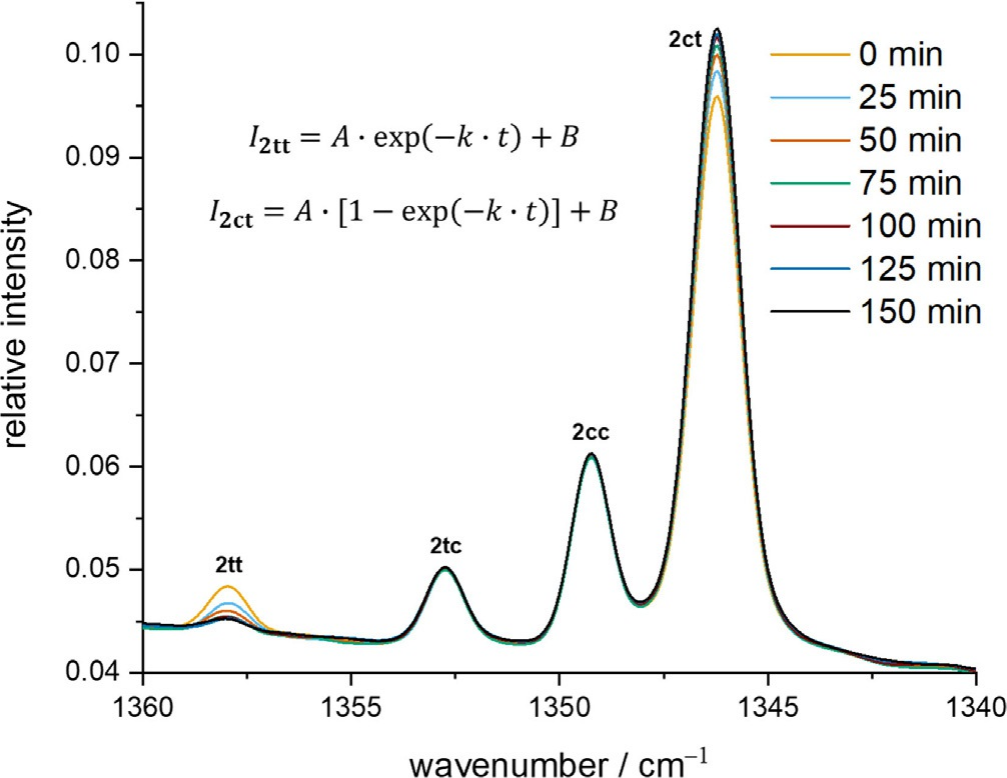
Kinetic evaluation of bands of the conformers of **2** in an Ar matrix at 3 K. A 4.5 μm cut‐off filter was used to limit photochemistry induced by the Globar of the spectrometer. The bands of **2 tt** vanish with a half‐life of ca. 30 min. Measurements were repeated every 5 min, but for clarity not all spectra are displayed here.

We obtained the experimental tunneling half‐life of the reaction **2 tt**→**2 ct** by following the decay (increase) of the strongest bands of **2 tt** (**2 ct**). A first‐order kinetic model with the concentrations of **2 ct** and **2 tt** (that are proportional to the observed band intensities *I*), the pre‐exponential factor *A* (equal to the band intensity at *t=*0) and the time *t* as in Figure 7 was used. The offset *B* needs to be included because the bands of **2 tt** will not reach the asymptotical value of zero.

Especially when measuring the spectra with the 4.5 μm cut‐off filter the band intensities of **2** are rather small making an exact analysis difficult. However, changing the temperature from 3 to 20 K or placing the cut‐off filter between the spectrometer and the matrix window does not change the results significantly neither in Ar nor in N_2_ (see the Supporting Information for details). The half‐life remains between 25 and 45 min for both matrix materials.

We computed the tunneling half‐lives for all conceivable reactions between the thiolimine conformers with canonical variational theory (CVT) in conjunction with small‐curvature tunneling (SCT). This method has often been demonstrated to yield reliable results for matrix‐isolated species.^[24]^ Our results are shown in Table 1. In agreement with the experiment, **2 tt**→**2 ct** is computed to be the fastest reaction. Even though according to the computations **2 cc**→**2 tc** should be observable at laboratory time scales, we did not observe such a process. This becomes apparent when applying a scaling factor (219.5) to the computed half‐lives accounting for the deviation of the experimental (ca. 30 min) and the computed value of the reaction **2 tt**→**2 ct**. A somewhat more realistic value for **2 cc**→**2 tc** results (1361 d), far outside reasonable experimental time scales. Similar results were recently discussed for matrix isolated thioacetamide.^[20]^ CVT/SCT underestimates tunneling half‐lives probably because the matrix environment has a stabilizing effect on trapped species. As expected, for the reactions **2 tt**→**2 tc** and **2 cc**→**2 ct** the computed tunneling half‐lives are much longer, due to the greater widths of the corresponding reaction barriers.

**Table 1:** Unscaled computed tunneling half‐lives at the CVT/SCT//B3LYP/6–311++G(3df,3pd) level of theory. Experimentally, only **2 tt→2 ct** is observed.

Reaction	Symmetry number^[a]^	Tunneling half‐life	
		Computation	Experiment
**2 tt**→**2 ct**	2	8.2 s	25–45 min
**2 cc**→**2 tc**	2	6.2 d	n.o.^[b]^
**2 tt**→**2 tc**	1	338 d	n.o.^[b]^
**2 cc**→**2 ct**	1	389 d	n.o.^[b]^

[a] Accounts for the degeneracy of the reaction coordinate [b] Not observed.

In contrast to the findings for thiourea[^17^, ^18^] but in agreement with those for thioacetamide^[20]^ no back‐reaction to **1** was observed after keeping the matrix for several days in the dark. Substitution apparently has a strong effect on the tunneling half‐lives in thioamides. In this respect it is worth mentioning the different reactivities of amides, thioamides, and selenoamides. Such studies were carried out by Nowak et al. on thiourea[^17^, ^18^] and selenourea.^[51]^ The tunneling half‐lives in Ar at 10 K are 52 h and 16 h with activation barriers of 25.1 kcal mol^−1^ and 22.7 kcal mol^−1^, respectively, determined at the MP2/6–311++G(2d,p) level of theory.^[51]^ At this level the minima are 14.8 kcal mol^−1^ and 16.0 kcal mol^−1^ apart from each other with the thioamide (selenoamide) form being more stable.^[51]^ For matrix isolated urea such a reaction has not been reported, probably indicating a tunneling half‐life too long to be observable. A very recent computational study employing the least‐action tunneling (LAT) method and CVT/SCT supports the experimental trend in this series.^[52]^

As the tunneling rate increases for heavier homologues in this series, we aimed to determine whether this trend is also apparent in the parent structures. Table 2 shows the energies associated with the enolimine→amide tautomerization. The tunneling half‐lives obtained with the Wentzel–Kramers–Brillouin (WKB) method are given in Table 2. Although the M06‐2X/6–311++G(2d,p) level of theory predicts a decrease in the tunneling half‐life, all values would be much too high to observe the corresponding reaction at laboratory time scales. This result is in agreement with our experimental findings reported herein as well as the absence of a tunneling reaction in the formamide system, which has been investigated by Maier and Endres.^[31]^ We draw the conclusion that enolimine→amide tunneling is enabled by the effects of the (remote) substituent, whereas the parent systems themselves do not show such a reactivity intrinsically. This also implies that **1** and **2** might co‐exist in interstellar media as quenching of **2** only seems possible through bimolecular reactivity, which is highly unlikely under interstellar conditions.

**Table 2:** Relative energies, barriers, and tunneling half‐lives of the enol→ketone tautomerization at B3LYP/6‐311++G(3df,3pd) (M06‐2X/6‐311+G(2d,p) in brackets). The WKB method implemented in Tunnex^[53]^ was used.

	Formamide	Thioformamide	Selenoformamide
ΔΔ*H*_0_ between minima [kcal mol^−1^]	12.8 (11.1)	10.0 (9.7)	10.4 (11.1)
ΔΔ*H*_0_^≠^ enolimine→amide [kcal mol^−1^]	26.4 (33.6)	27.1 (28.0)	25.4 (25.5)
computed QMT half‐life [years]	2057 (44 210)	2307 (7079)	4703 (1315)

Substituent effects have been shown to affect tunneling rates before, most prominently in systematic studies on carboxylic acids[^54^, ^55^, ^56^, ^57^, ^58^, ^59^, ^60^, ^61^] and highly reactive hydroxycarbenes.[^62^, ^63^, ^64^, ^65^, ^66^, ^67^, ^68^, ^69^] In hydroxycarbenes +I‐donating substituents like methyl result in reduced tunneling half‐lives for [1,2]H‐shifts towards the corresponding aldehyde.^[69]^ Contradicting results are obtained for rotamerizations in carbonic acid^[70]^ and its monomethyl ester.^[71]^ When comparing our result for the reaction **2 tt**→**2 ct** (ca. 30 min) with the analogue reaction in thioacetamide (ca. 80 min)^[20]^ a similar opposing trend is apparent. The absence of thiolimine→thioamide tunneling in thioacetamide^[19]^ and thioformamide in contrast to thiourea^[18]^ is puzzling, since π‐donating substituents incorporating heteroatoms (like NH_2_) lead to prolonged tunneling half‐lives for [1,2]H‐shifts in hydroxycarbenes.^[69]^ Hence, substituent effects apparently do not affect QMT in a systematic way when comparing distinct compound classes with one another. On the one hand, barrier heights (and in this regard also barrier widths) are dependent on remote substitution. Also, the matrix material might interact differently with various derivatives of the same compound class. A very recent study in the realm of heavy‐atom tunneling^[72]^ addresses the interplay of these effects.^[73]^ Disentangling the influences on tunneling half‐lives to draw a clearer picture how to effectively control QMT remains an ongoing endeavor.

## Conclusions

We isolated thioformamide in Ar and N_2_ matrices and generated four conceivable conformers of the energetically higher lying thiolimine tautomer by UV excitation. One of these conformers (**2 tt**) vanished with a half‐life of ca. 30 min to form **2 ct**. This process can be ascribed to QMT and is analogous to the findings reported recently on thioacetamide.^[20]^

In contrast to thiourea a tunneling reaction back to the thioamide tautomer has not been observed. This illustrates that (remote) substitution can enhance or inhibit QMT as it has already been shown in previous studies. As the effect of the substituent on QMT is intricate, further research is planned in our laboratories to fine‐tune QMT and further establish its role as the ‘third reactivity paradigm’.^[26]^

## Experimental Section

Thioformamide and its *N*‐deuterated isotopologues were prepared according to the procedure by Willstätter and Wirth.^[74]^ For details see the Supporting Information. A typical matrix isolation experiment is described in the Supporting Information. Computational details can be found there as well.

Deposition numbers 2033186, and 2032761 contain the supplementary crystallographic data for this paper. These data are provided free of charge by the joint Cambridge Crystallographic Data Centre and Fachinformationszentrum Karlsruhe Access Structures service.

## Conflict of interest

The authors declare no conflict of interest.

## Supporting information

As a service to our authors and readers, this journal provides supporting information supplied by the authors. Such materials are peer reviewed and may be re‐organized for online delivery, but are not copy‐edited or typeset. Technical support issues arising from supporting information (other than missing files) should be addressed to the authors.

Supplementary
