# Supplementary material for: Characterization of the Simplest Thiolimine: The Higher Energy Tautomer of Thioformamide
Source: Chemistry. 2021 Mar 12;27(22):6732–9. doi: 10.1002/chem.202005188 (PMC8252572; doi:10.1002/chem.202005188)
Supplement: Supplementary file 1 — Supplementary [file CHEM-27-6732-s001.pdf]

Chemistry–A European Journal

Supporting Information

Characterization of the Simplest Thiolimine: The Higher Energy Tautomer of Thioformamide

Bastian Bernhardt,[a] Friedemann Dressler,[a] André K. Eckhardt,[a] Jonathan Becker,[b] and Peter R. Schreiner*[a]

Supporting Information

Table of Contents

Experimental Procedures **S3**
Synthesis of Thioformamide[1] S3
Matrix Isolation S4
Computational Details S4
Spectral and Diffraction Data; Computational Results **S5**
NMR Spectra S5
Crystallographic Data Collection and Processing S10
Independent Atom Model (IAM) Refinement S11
Hirshfeld Atom Refinement (HAR) S15
Matrix-IR Spectra S18
IR Spectroscopic Data S25
Matrix-UV/Vis Spectrum in N_2 ..... S31
UV/Vis Spectroscopic Data S32
Natural Bond Orbital Analysis S35
Potential Energy Surface and Cartesian Coordinates of Selected Structures S37
Tunneling Computations S46
Kinetic Analysis S49
Other Matrix Isolation Studies of Thioamides S51
More Isomers of Thioformamide S53
References **S54**
Full Citations for Electronic Structure Codes **S55**
Author Contributions **S56**

Experimental Procedures

Synthesis of Thioformamide[1]

8.60 g (19.3 mmol) phosphorus pentasulfide were added in six portions to 12.21 g (271.1 mmol, 1.4 equiv.) formamide at 0 °C within 40 min. After 1 h, the solution was warmed to room temperature and stirred for 2 h. 30 mL of diethyl ether were added and the solution was stirred for 12 h. The ether phase was decanted and the resulting yellow viscous mass extracted with diethyl ether, until the ether phase was colorless (6 x 100 mL). After removal of the solvent under reduced pressure, crude thioformamide was obtained and dissolved in 30 mL water. The solution was saturated with ammonium sulfate. Thioformamide was extracted with diethyl ether, until the ether phase was colorless (8 x 50 mL). The collected ether extracts were dried over sodium sulfate and the solvent was removed under reduced pressure. The obtained yellow oil was diluted in 100 mL diethyl ether and about 15 g phosphorus pentoxide was added. After 14 h, the solution was filtered and concentrated to 10 mL. The solution was cooled to −15 °C and thioformamide precipitated upon addition of dry pentane. The precipitate was dissolved in a minimum of dry ethyl acetate and cooled to −50 °C, where colorless crystals were obtained. After filtration, the crystals were dried under reduced pressure ($5 \cdot 10^{-2}$ mbar) at 0 °C (at higher temperatures thioformamide sublimated). 2.95 g (48.3 mmol, 25%) of thioformamide were obtained as a pure colorless solid. Thioformamide is stable under air for 30 min and afterwards begins to decompose to a yellow oil. This oil is insoluble in any common solvent, which might hint towards the formation of poly- or oligomers. Thioformamide is stable for more than a week under argon at −20 °C.
^{1}H-NMR (400 MHz, CD_2Cl_2): δ = 9.43 (dd, J = 16.3, 5.6 Hz, 1H), 8.00-7.44 (m, 1H), 7.44-6.92 (m, 1H) ppm.
^{13}C-NMR (101 MHz, CD_2Cl_2): δ = 193.5 ppm.

Synthesis of *N*,*N*-dideuterated Thioformamide

Crude thioformamide was dissolved in *O*-deuterated methanol and the solvent was evaporated. This procedure was repeated eight times. The crude dideuterated thioformamide was dissolved in deuterium oxide and the solution was saturated with deuterated ammonium sulfate followed by extraction with dry diethyl ether and drying over sodium sulfate. To obtain *N*,*N*-dideuterated thioformamide, the product was not further purified by drying over phosphorus pentoxide and recrystallization in ethyl acetate, because the standard procedure led to a decrease of deuteration. Hence, purification yields a mixture of undeuterated, *N*-monodeuterated (two isotopomers), and (mainly) *N*,*N*-dideuterated thioformamide.
^{1}H-NMR (600 MHz, CD_2Cl_2): δ = 9.43 (s, 1H) ppm.
^{2}H-NMR (92 MHz, CD_2Cl_2): δ = 7.73 (brs, 1D), 7.19 (brs, 1D) ppm.
^{13}C-NMR (151 MHz, CD_2Cl_2): δ = 193.3 ppm.

Matrix Isolation

For matrix isolation experiments we used a Sumitomo cryostat system consisting of an RDK 408D2 closed-cycle refrigerator cold head and an F-70 compressor unit. The vacuum shroud was equipped with polished potassium bromide windows allowing for infrared (IR) measurements. A polished cesium iodide window (Korth Kristalle GmbH) mounted in the sample holder on the cold head served as the matrix support. The temperature of the matrix was determined with silicon diodes at the base of the sample holder. The deposition temperature was 15 K. The storage bulb containing thioformamide was cooled to −20 °C for deposition. For deposition on the matrix window argon (Ar) or dinitrogen (N_2) (gas purity of 99.999%) was transferred to a 2 L storage gas balloon. The balloon was refilled and flushed three times with Ar or N_2, respectively, before every experiment. UV irradiation of the deposited sample was carried out with a high-pressure mercury lamp (SP200) or an LOT Oriel system equipped with a double Czerny-Turner monochromator and a 150 W high pressure Hg(Xe) arc lamp (Hamamatsu Photonics) at 254 nm. For some experiments broadband irradiation > 320 nm was achieved by placing a cut-off filter in front of the lamp. IR spectra were recorded with a Bruker Vertex 70 FTIR spectrometer (standard potassium bromide or wide-range beams-splitter) at 3 K. Spectral data were recorded in the range from 7000 cm^{-1} to 350 cm^{-1} with a resolution of 0.7 cm^{-1}. Each measurement consisted of 50 scans. Measurements in the ultra-violet/visible (UV/Vis) region were conducted on a Jasco V-760 spectrophotometer in the range from 190 nm to 900 nm with a resolution of 1 nm. Background spectra were measured against the cold matrix window before deposition. The matrix was kept in the dark during deposition and between measurements in order to prevent unwanted photochemistry.

Computational Details

DFT computations at the B3LYP/6-311++G(3df,3pd)[2,3] and the M06-2X/6-311+G(2d,p)[4] levels of theory were conducted with Gaussian16 Revision B.01 (for full citations of the electronic structure codes used herein see the end of this document). An ultrafine grid and very tight conversion criteria were used. Anharmonic frequencies were computed at the B3LYP/6-311++G(3df,3pd) level of theory by using the keyword Freq=Anharmonic. All minima on the potential energy surface featured only real frequencies while every optimized transition state displayed exactly one imaginary frequency. Absorptions in the UV/Vis spectral region were computed at the B3LYP/6-311++G(3df,3pd) level of theory by using the keyword TD=(Singlets,Root=1,NStates=6).

For the tunneling computations the intrinsic reaction coordinate was followed for every transition state until the interconnected minima were obtained. For every structure on these reaction paths zero-point vibrational energies were computed by using the keyword Freq=TProjected and the algorithm implemented in make_projections. Tunneling half-lives were computed from these data with Tunnex.[5] Additionally, tunneling half-lives were also computed with Polyrate at the CVT/SCT//B3LYP/6-311++G(3df,3pd) and the CVT/SCT//M06-2X/6-311+G(2d,p) levels of theory.

All coupled-cluster computations at the CCSD(T)/cc-pVTZ and the AE-CCSD(T)/aug-cc-pCVTZ levels of theory[6–11] were performed with CFOUR (for full citations of the electronic structure codes used herein see the end of this document). The keywords ABCDTYPE=AOBASIS, CC_PROG=ECC, SCF_CONV=8, and CC_CONV=10 were used in every computation. For geometry optimizations CONVERGENCE=8 was used additionally. Harmonic frequencies were computed analytically by adding VIB=EXACT.

Spectral and Diffraction Data; Computational Results

NMR Spectra

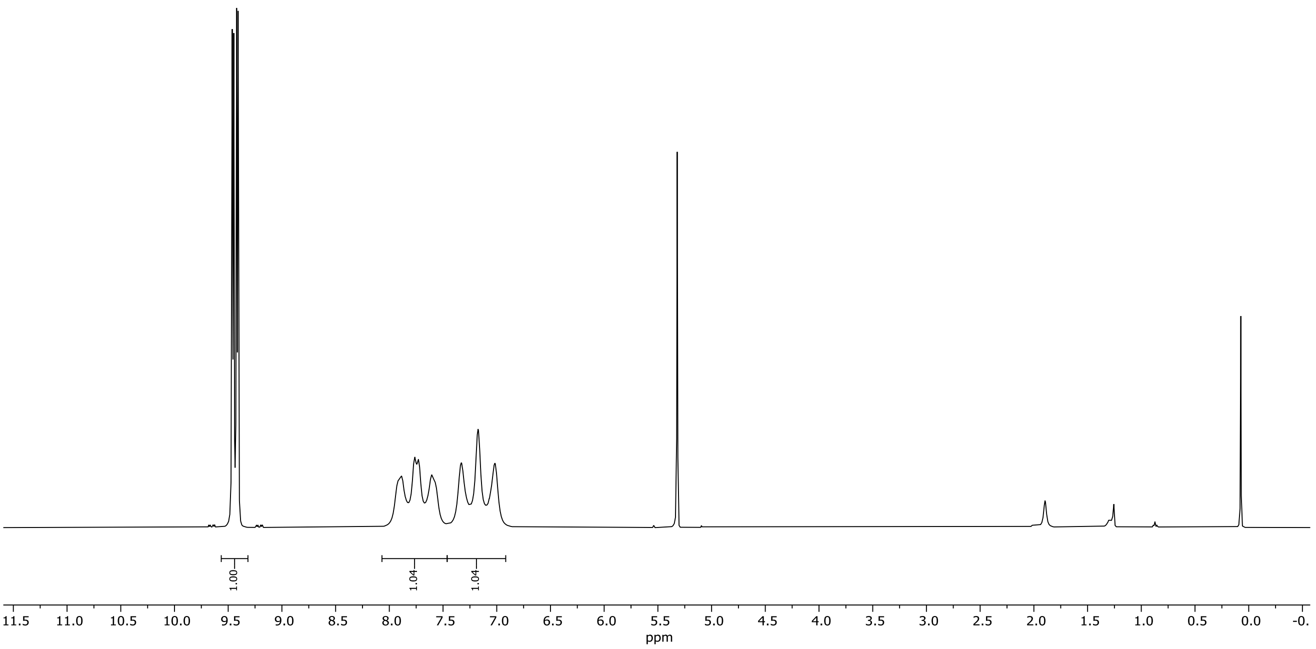

Figure S1. ^{1}H-NMR (400 MHz, CD_2Cl_2) of thioformamide.

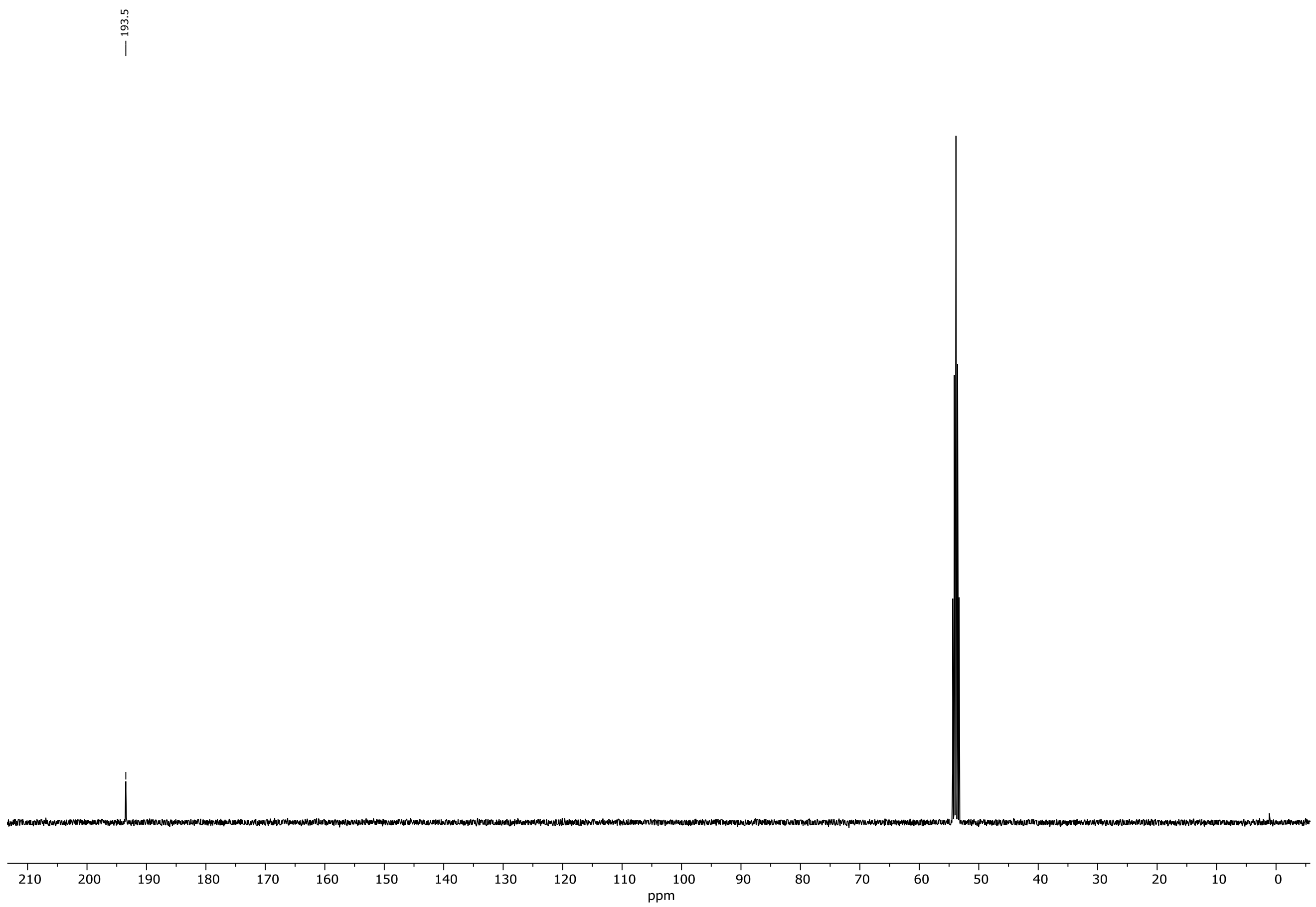

Figure S2. ^{13}C-{^{1}H}-NMR (101 MHz, CD_2Cl_2) of thioformamide.

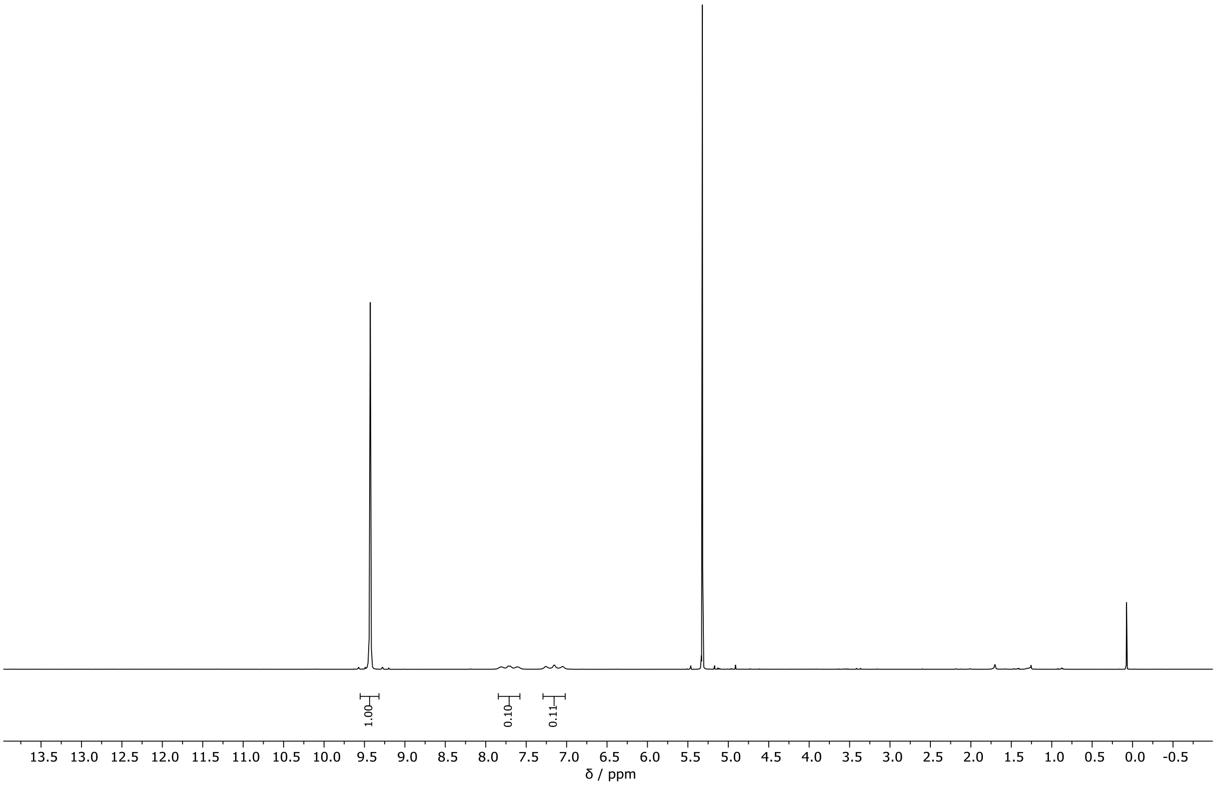

Figure S3. ^{1}H-NMR (600 MHz, CD_2Cl_2) of *N*,*N*-dideuterated thioformamide.

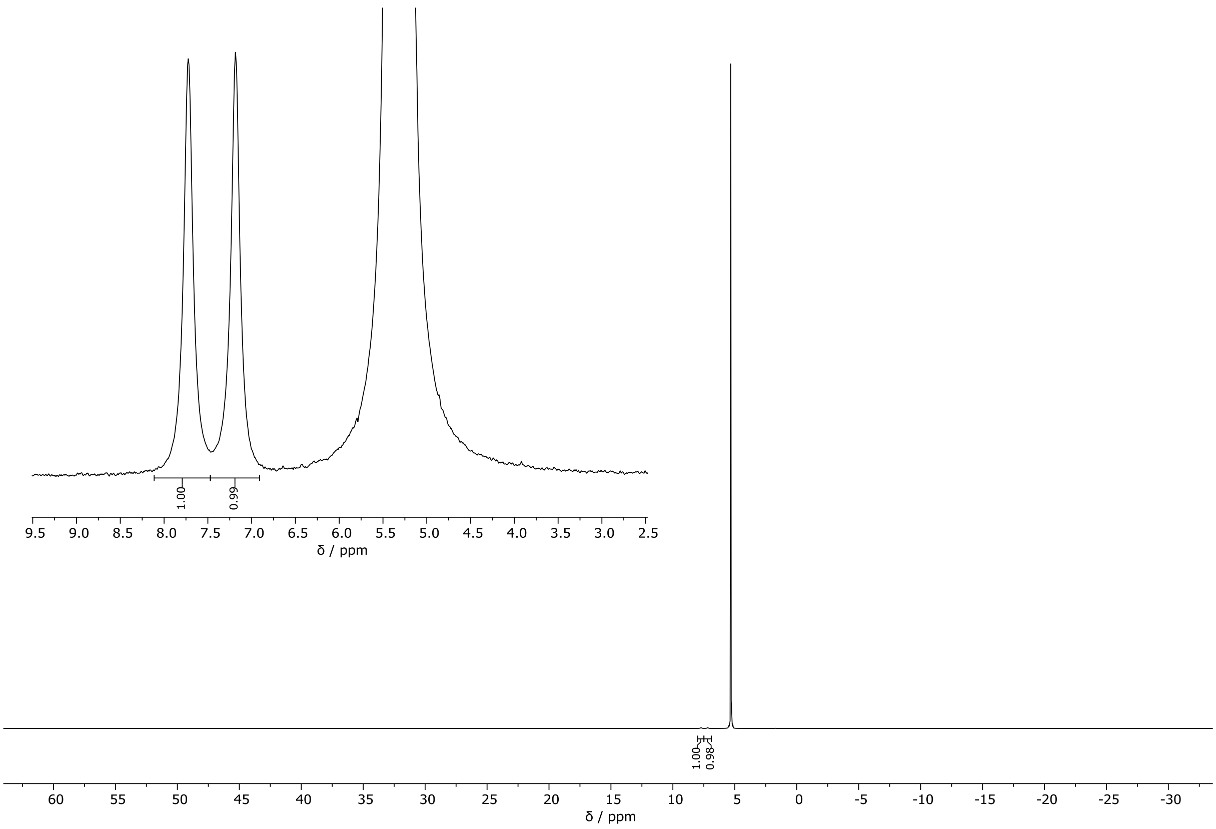

Figure S4. ^{2}H-NMR (92 MHz, CD_2Cl_2) of *N*,*N*-dideuterated thioformamide.

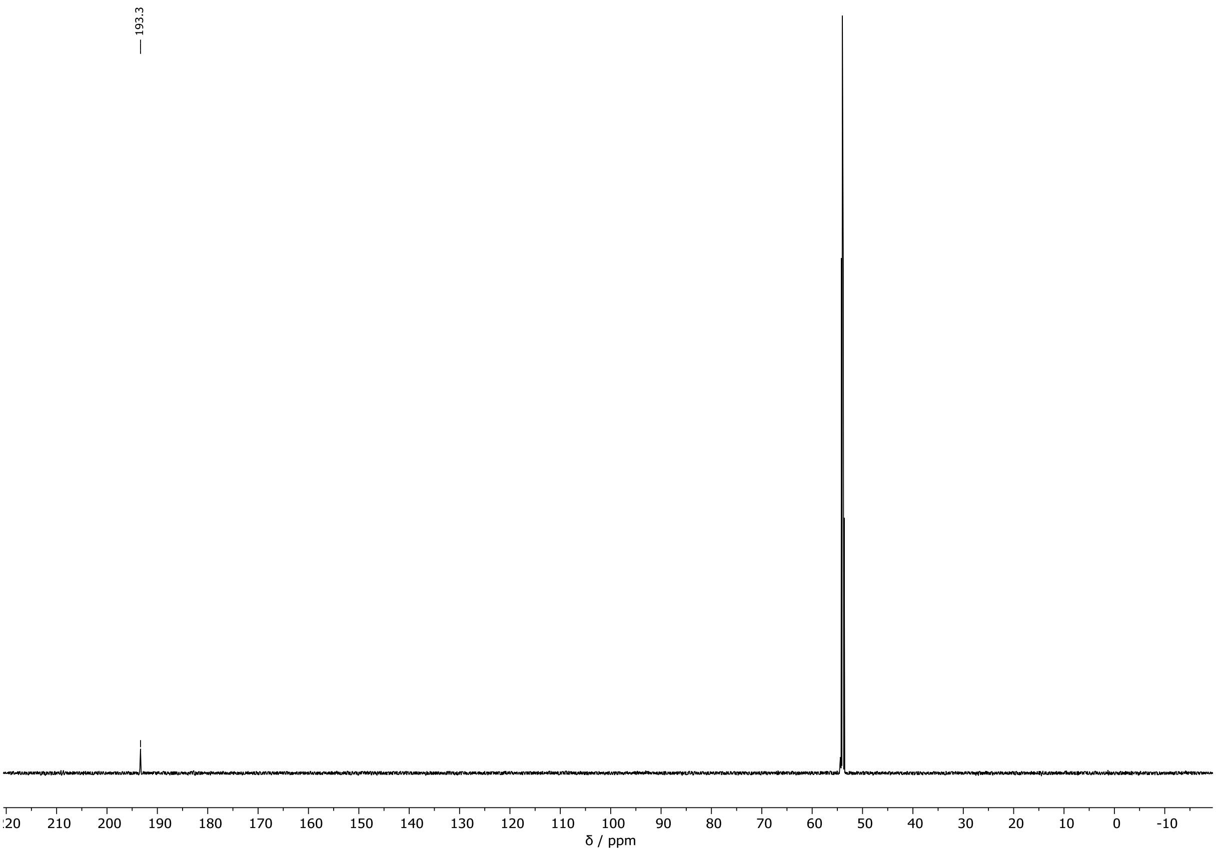

Figure S5. ^{13}C-{^{1}H}-NMR (150 MHz, CD_2Cl_2) of *N*,*N*-dideuterated thioformamide.

Crystallographic Data Collection and Processing

Crystals of thioformamide · ethyl acetate were selected and prepared for measurement in a low temperature (about −50 °C) nitrogen stream to avoid melting. Diffraction data were collected at low temperatures (100 K) using φ- and ω-scans on a BRUKER D8 Venture system equipped with dual IµS microfocus sources, a PHOTON100 detector and an OXFORD CRYOSYSTEMS 700 low temperature system. Mo-K_α radiation with a wavelength of 0.71073 Å and a collimating Quazar multilayer mirror were used. Semi-empirical absorption correction from equivalents was applied using SADABS-2016/2.[12] The structure was solved by the dual space algorithm implemented in SHELXT2014/5[13] in the orthorhombic space group *Pnma*.

The crystallographic data have been deposited at the Cambridge Crystallographic Data Centre as CCDC No. 2033186 and 2032761 (with and without Hirshfeld atom refinement, see below) and can be obtained free of charge at https://www.ccdc.cam.ac.uk/structures/.

Independent Atom Model (IAM) Refinement

The refinement was performed against F^2 on all data by full-matrix least squares using SHELXL2018/3.[13] All non-hydrogen atoms were refined anisotropically. Hydrogen atoms were located in the difference map and were set to ideal distances. The isotropic displacement parameters of all hydrogen atoms were fixed to 1.2x (NH_2, CH_2, and CH) or 1.5x (CH_3) the U_{eq} value of the atom they are linked. Ethyl acetate showed two possible conformers due to rotation of its methyl group. The good data quality allowed for refinement of those. The disorder ratio was allowed to refine freely and converged to 0.72(2).

Table S1. Crystal data and structure refinement of thioformamide · ethyl acetate.

CCDC No	2033186	
Empirical formula	C_5 H_{11} N O_2 S	
Formula weight	149.21	
Temperature	100(2) K	
Wavelength	0.71073 Å	
Crystal system	Orthorhombic	
Space group	*Pnma*	
Unit cell dimensions	a = 7.8517(3) Å	α= 90°.
	b = 6.5006(2) Å	β= 90°.
	c = 15.3960(5) Å	γ = 90°.
Volume	785.82(5) $Å^3$	
Z	4	
Density (calculated)	1.261 Mg/m^3	
Absorption coefficient	0.347 mm^{-1}	
F(000)	320	
Crystal size	0.547 x 0.301 x 0.178 mm^3	
Theta range for data collection	2.646 to 32.028°.	
Index ranges	$-11 \le h \le 11$, $-9 \le k \le 9$, $-22 \le l \le 22$	
Reflections collected	26957	
Independent reflections	1474 [R(int) = 0.0533]	
Completeness to theta = 25.242°	100.0 %	
Absorption correction	Semi-empirical from equivalents	
Refinement method	Full-matrix least-squares on F^2	
Data / restraints / parameters	1474 / 10 / 81	
Goodness-of-fit on F^2	1.078	
Final R indices [I>2σ(I)]	R1 = 0.0285, wR2 = 0.0686	
R indices (all data)	R1 = 0.0362, wR2 = 0.0722	
Extinction coefficient	0.021(2)	
Largest diff. peak and hole	0.395 and −0.385 e.$Å^{-3}$	

Table S2. Atomic coordinates (x10^4) and equivalent isotropic displacement parameters (Å^2 x10^3) of thioformamide · ethyl acetate. U(eq) is defined as one third of the trace of the orthogonalized Uij tensor.

	x	y	z	U(eq)
S(1)	3853(1)	7500	1953(1)	16(1)
N(1)	4763(1)	7500	3612(1)	16(1)
C(1)	3555(2)	7500	3025(1)	14(1)
O(11)	4156(1)	7500	5444(1)	22(1)
O(12)	6629(1)	7500	6180(1)	17(1)
C(11)	4104(2)	7500	7002(1)	19(1)
C(12)	4929(2)	7500	6128(1)	16(1)
C(13)	7556(2)	7500	5359(1)	18(1)
C(14)	9427(2)	7500	5573(1)	21(1)

Table S3. Bond lengths in Å and angles in degrees of thioformamide · ethyl acetate. The symmetry transformation #1 x,−y+3/2,z was used to generate equivalent atoms.

S(1)-C(1)	1.6663(12)	C(1)-N(1)-H(1A)	118.8(12)
N(1)-C(1)	1.3105(15)	C(1)-N(1)-H(1B)	121.3(12)
N(1)-H(1A)	0.872(14)	H(1A)-N(1)-H(1B)	120.0(17)
N(1)-H(1B)	0.871(14)	N(1)-C(1)-S(1)	125.59(10)
C(1)-H(1)	0.952(13)	N(1)-C(1)-H(1)	115.8(10)
O(11)-C(12)	1.2157(15)	S(1)-C(1)-H(1)	118.6(10)
O(12)-C(12)	1.3366(15)	C(12)-O(12)-C(13)	116.54(10)
O(12)-C(13)	1.4593(15)	C(12)-C(11)-H(11A)	110.1(12)
C(11)-C(12)	1.4931(17)	C(12)-C(11)-H(11B)	111.2(16)
C(11)-H(11A)	0.969(14)	H(11A)-C(11)-H(11B)	109.2(15)
C(11)-H(11B)	0.983(17)	C(12)-C(11)-H(11C)	113(5)
C(11)-H(11C)	0.98(2)	C(12)-C(11)-H(11D)	108(3)
C(11)-H(11D)	0.979(19)	H(11C)-C(11)-H(11D)	112(4)
C(11)-H(11A)#1	0.969(14)	C(12)-C(11)-H(11A)#1	110.1(12)
C(11)-H(11D)#1	0.979(19)	H(11A)-C(11)-H(11A)#1	107(2)
C(13)-C(14)	1.5050(19)	H(11B)-C(11)-H(11A)#1	109.2(15)
C(13)-H(13)	0.976(11)	C(12)-C(11)-H(11D)#1	108(3)
C(13)-H(13)#1	0.976(11)	H(11A)-C(11)-H(11D)#1	142(3)
C(14)-H(14A)	0.975(12)	H(11B)-C(11)-H(11D)#1	53(3)
C(14)-H(14B)	0.986(15)	H(11A)#1-C(11)-H(11D)#1	61(3)
C(14)-H(14A)#1	0.975(12)	O(11)-C(12)-O(12)	123.40(11)
		O(11)-C(12)-C(11)	124.30(12)
		O(12)-C(12)-C(11)	112.30(11)
		O(12)-C(13)-C(14)	107.27(10)
		O(12)-C(13)-H(13)	108.9(7)
		C(14)-C(13)-H(13)	112.1(7)
		O(12)-C(13)-H(13)#1	108.9(7)
		C(14)-C(13)-H(13)#1	112.1(7)
		H(13)-C(13)-H(13)#1	107.5(15)
		C(13)-C(14)-H(14A)	111.6(8)
		C(13)-C(14)-H(14B)	109.4(13)
		H(14A)-C(14)-H(14B)	107.6(11)
		C(13)-C(14)-H(14A)#1	111.6(8)
		H(14A)-C(14)-H(14A)#1	108.7(16)
		H(14B)-C(14)-H(14A)#1	107.6(11)

Table S4. Anisotropic displacement parameters (Å^2 x 10^3) for thioformamide · ethyl acetate. The anisotropic displacement factor exponent takes the form: $-2p^2 (h^2 a^{*2} U^{11} + ... + 2 h k a^* b^* U^{12})$.

	U^{11}	U^{22}	U^{33}	U^{23}	U^{13}	U^{12}
S(1)	12(1)	24(1)	13(1)	0	−1(1)	0
N(1)	13(1)	21(1)	13(1)	0	0(1)	0
C(1)	12(1)	17(1)	13(1)	0	0(1)	0
O(11)	20(1)	31(1)	14(1)	0	−1(1)	0
O(12)	18(1)	22(1)	12(1)	0	0(1)	0
C(11)	22(1)	21(1)	13(1)	0	3(1)	0
C(12)	19(1)	14(1)	14(1)	0	1(1)	0
C(13)	19(1)	22(1)	12(1)	0	1(1)	0
C(14)	17(1)	27(1)	20(1)	0	−1(1)	0

Table S5. Hydrogen coordinates (x10^4) and isotropic displacement parameters (Å^2 x 10^3) of thioformamide · ethyl acetate.

	x	y	z	U(eq)
H(1A)	5821(18)	7500	3441(12)	19
H(1B)	4520(20)	7500	4165(9)	19
H(1)	2420(18)	7500	3242(11)	17
H(11A)	4460(20)	8700(30)	7330(12)	28
H(11B)	2860(20)	7500	6951(18)	28
H(11C)	4930(70)	7500	7480(30)	28
H(11D)	3340(50)	8690(50)	7030(30)	28
H(13)	7225(15)	8711(17)	5024(8)	21
H(14A)	9750(17)	8720(20)	5903(8)	32
H(14B)	10090(30)	7500	5031(12)	32

Table S6. Hydrogen bonds of thioformamide · ethyl acetate in Å and degrees. The symmetry transformations #1 x,−y+3/2,z and #2 x+1/2,y,−z+1/2 were used the generate equivalent atoms.

N-H···S	d(N-H)	d(H···S)	d(N···S)	<(NHS)
N(1)-H(1A)···S(1)#2	0.872(14)	2.456(14)	3.3274(11)	176.7(16)
N(1)-H(1B) ···O(11)	0.871(14)	1.991(14)	2.8606(14)	176.0(16)

Hirshfeld Atom Refinement (HAR)

The HAR[14] was performed with the HARt interface in Olex2[15] using the Kohn-Sham/becke88lyp/SCCF method with the def2-TZVP basis set.[16] The IAM structure reported above was used as the input. For the wavefunction computation a full molecule of thioformamide · ethyl acetate (two asymmetric units) was used. The computation was performed with a cluster of point charges and dipoles, simulating the crystal effect of all full molecules within a 5 Å radius. Aspherical atomic scattering factors ("Hirshfeld atoms") were obtained from the computed electron density and used for the structure refinement. Hydrogen atoms were refined isotropically and completely free. Analysis of the normal probability distribution and structure factor differences (experiment/theory) over sin(θ) / λ indicate a partially problematic data set. This has to be attributed to the crystal quality which was limited by the difficult handling of the crystals.

Table S7. Additional refinement data for thioformamide · ethyl acetate (HAR).

CCDC No	2032761
Data / restraints / parameters	1274 / 0 / 74
Final R indices [I>2σ(I)]	R1 = 0.0334, wR2 = 0.0295
R indices (all data)	R1 = 0.0334, wR2 = 0.0295
Largest diff. peak and hole	0.2183 and −0.3337 e.Å^{-3}

Table S8. Atomic coordinates (x10^5) and equivalent isotropic displacement parameters (Å^2 x 10^4) for thioformamide · ethyl acetate (HAR). U(eq) is defined as one third of the trace of the orthogonalized Uij tensor.

	x	y	z	U(eq)
S(1)	38520(5)	75000	19528(3)	144(2)
N(1)	47671(16)	75000	36127(9)	151(6)
C(1)	35530(18)	75000	30248(11)	142(7)
O(11)	41567(14)	75000	54444(7)	205(6)
O(12)	66275(13)	75000	61776(7)	163(6)
C(11)	41035(21)	75000	69981(11)	192(8)
C(12)	49333(19)	75000	61277(10)	146(7)
C(13)	75515(20)	75000	53629(11)	176(8)
C(14)	94277(21)	75000	55725(12)	206(9)
H(1A)	60808(247)	75000	34404(132)	188
H(1B)	45022(247)	75000	42739(137)	188
H(1)	21925(222)	75000	326956(113)	171
H(11A)	45478(165)	86019(185)	73889(81)	286
H(11B)	27542(246)	75000	69893(119)	286
H(13)	71694(143)	86984(159)	49792(79)	213
H(14A)	97861(161)	88438(204)	59525(86)	316
H(14B)	101759(229)	75000	49758(14)	316

Table S9. Bond lengths in Å and angles in degrees of thioformamide · ethyl acetate (HAR).

S(1)-C(1)	1.6671(16)	C(1)-N(1)-H(1A)	122.1(11)
O(11)-C(12)	1.2159(18)	C(1)-N(1)-H(1B)	122.0(11)
O(12)-C(12)	1.3324(18)	H(1A)-N(1)-H(1B)	116.0(15)
O(12)-C(13)	1.4490(18)	N(1)-C(1)-S(1)	125.42(11)
N(1)-C(1)	1.3145(18)	N(1)-C(1)-H(1)	117.1(9)
N(1)-H(1a)	1.065(19)	S(1)-C(1)-H(1)	117.5(9)
N(1)-H(1b)	1.04(2)	C(12)-O(12)-C(13)	116.74(13)
C(11)-C(12)	1.490(2)	C(12)-C(11)-H(11A)	112.9(8)
C(13)-C(14)	1.508(2)	C(12)-C(11)-H(11B)	115.2(10)
C(1)-H(1)	1.133(17)	H(11A)-C(11)-H(11B)	110.9(10)
C(11)-H(11a)	0.998(12)	O(11)-C(12)-O(12)	123.41(15)
C(11)-H(11b)	1.059(19)	O(11)-C(12)-C(11)	123.97(15)
C(13)-H(13)	1.023(11)	O(12)-C(12)-C(11)	112.62(14)
C(14)-H(14a)	1.088(13)	O(12)-C(13)-C(14)	107.69(13)
C(14)-H(14b)	1.09(2)	O(12)-C(13)-H(13)	110.7(7)
		C(14)-C(13)-H(13)	114.2(7)
		C(13)-C(14)-H(14A)	111.6(7)
		C(13)-C(14)-H(14B)	110.2(10)
		H(14A)-C(14)-H(14B)	108.3(9)

Table S10. Anisotropic displacement parameters ($Å^2$ x 10^4) of thioformamide · ethyl acetate (HAR). The anisotropic displacement factor exponent takes the form: $-2\pi^2$ (h^2 a^{*2} U^{11} + ... + 2 h k a* b* U^{12}).

	U^{11}	U^{22}	U^{33}	U^{23}	U^{13}	U^{12}
S(1)	104(2)	215(2)	112(2)	0(0)	−11(1)	0(0)
N(1)	131(6)	211(7)	111(7)	0(0)	−10(5)	0(0)
C(1)	109(7)	188(7)	128(7)	0(0)	2(1)	0(0)
O(11)	179(6)	310(6)	125(6)	0(0)	−14(1)	0(0)
O(12)	174(5)	209(6)	107(5)	0(0)	−2(4)	0(0)
C(11)	218(9)	217(8)	140(8)	0(0)	24(7)	0(0)
C(12)	177(8)	151(7)	110(8)	0(0)	11(6)	0(0)
C(13)	180(8)	214(8)	135(7)	0(0)	2(6)	0(0)
C(14)	162(8)	250(9)	205(9)	0(0)	−7(6)	0(0)

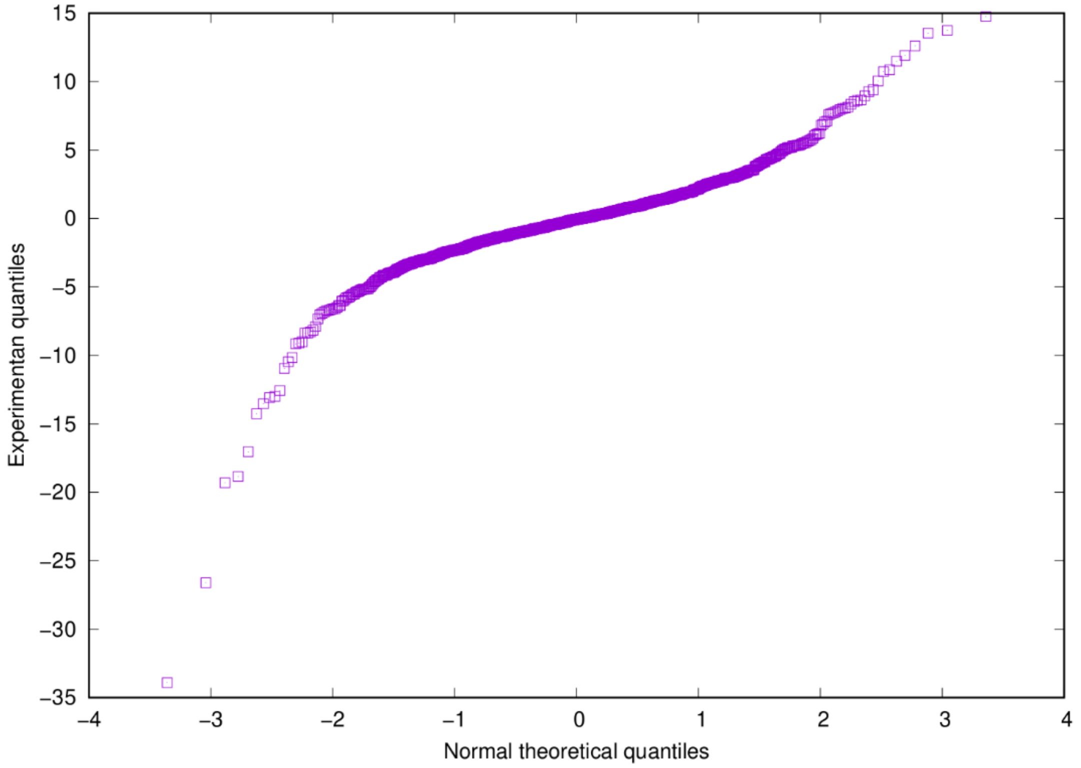

Figure S6. Normal probability plot (Q-Q plot) of the Hirshfeld Atom refinement.

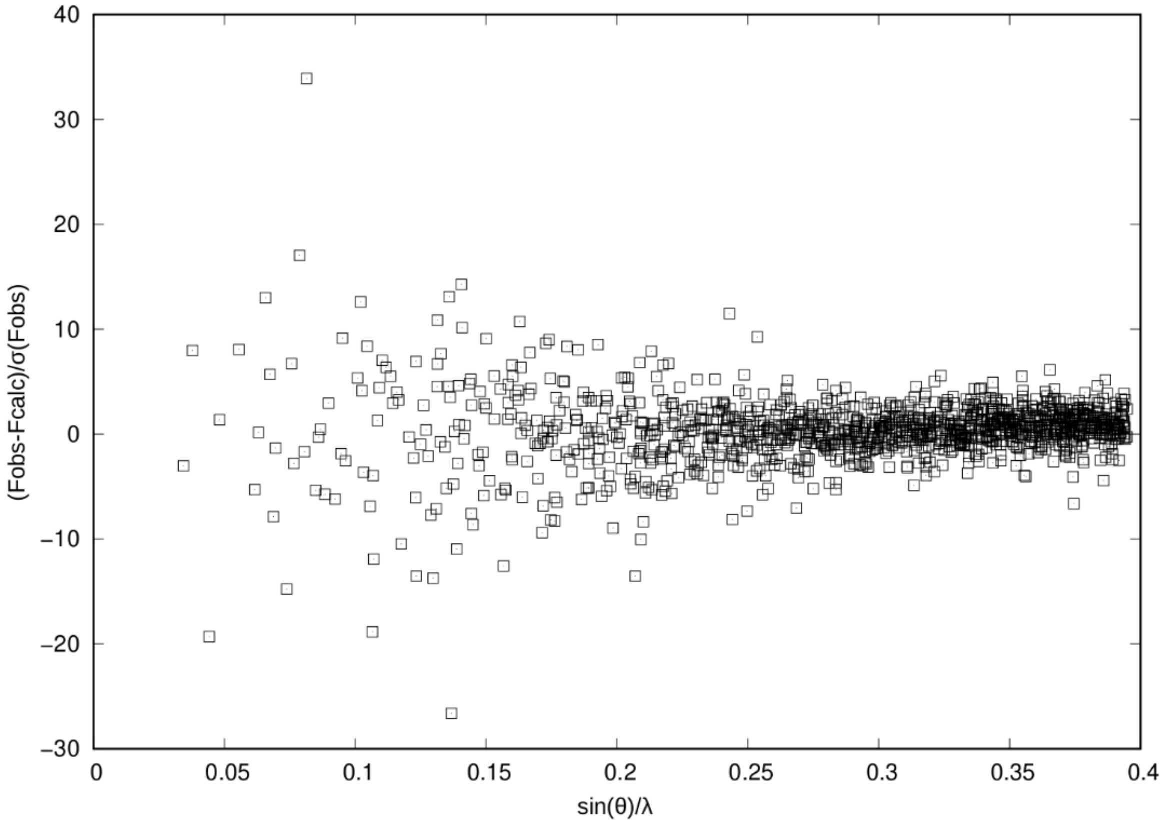

Figure S7. Scatter plot of the Hirshfeld Atom refinement.

Matrix-IR Spectra

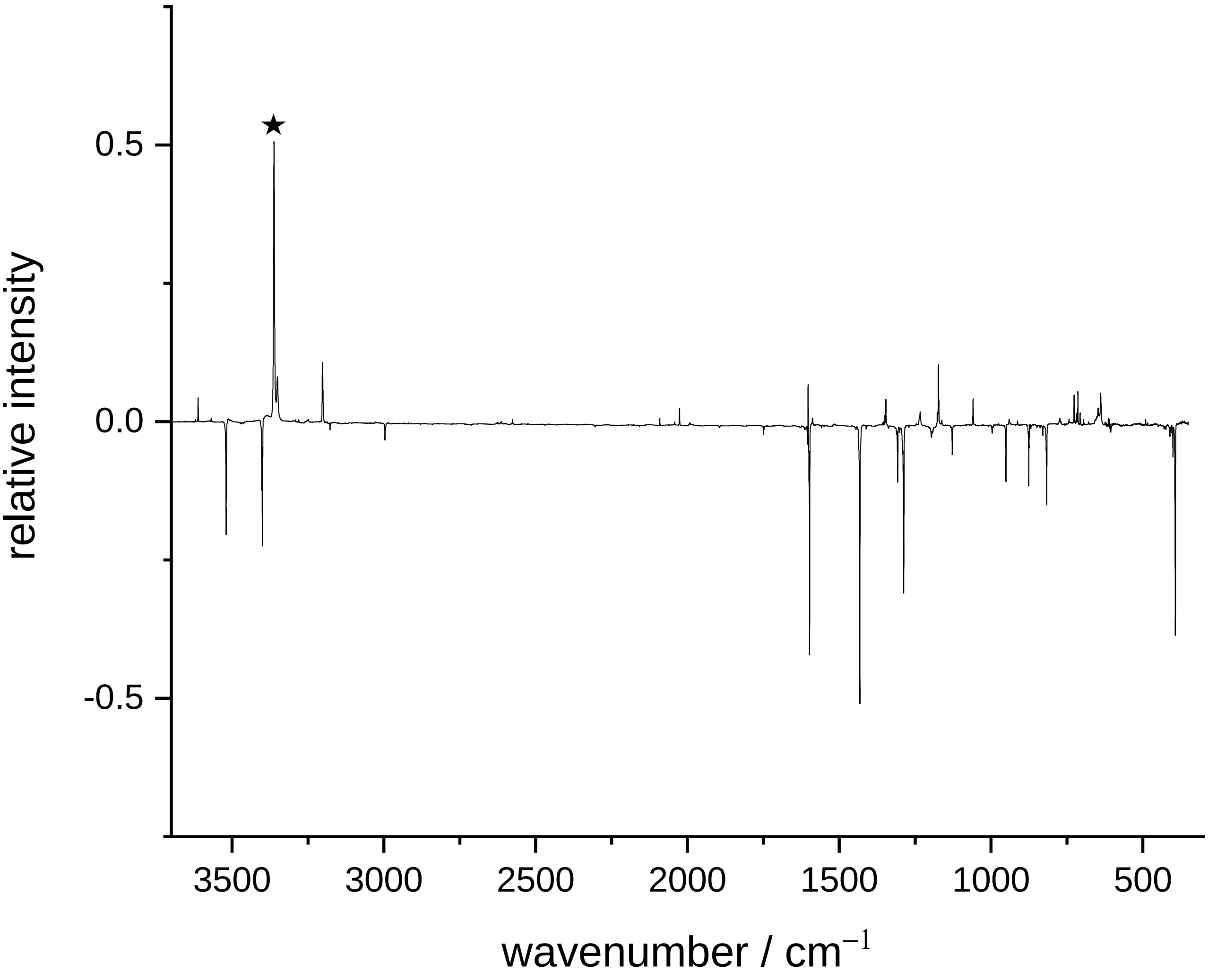

Figure S8. Difference spectrum of spectra measured before and after irradiation of **1** with 254 nm for 10 min in an Ar matrix. Downward pointing bands belong to **1**, upward pointing bands belong to **2**. We assign the high-intensity band at 3361.9 cm^{-1} marked with an asterisk tentatively to an NCH···H_2S complex.

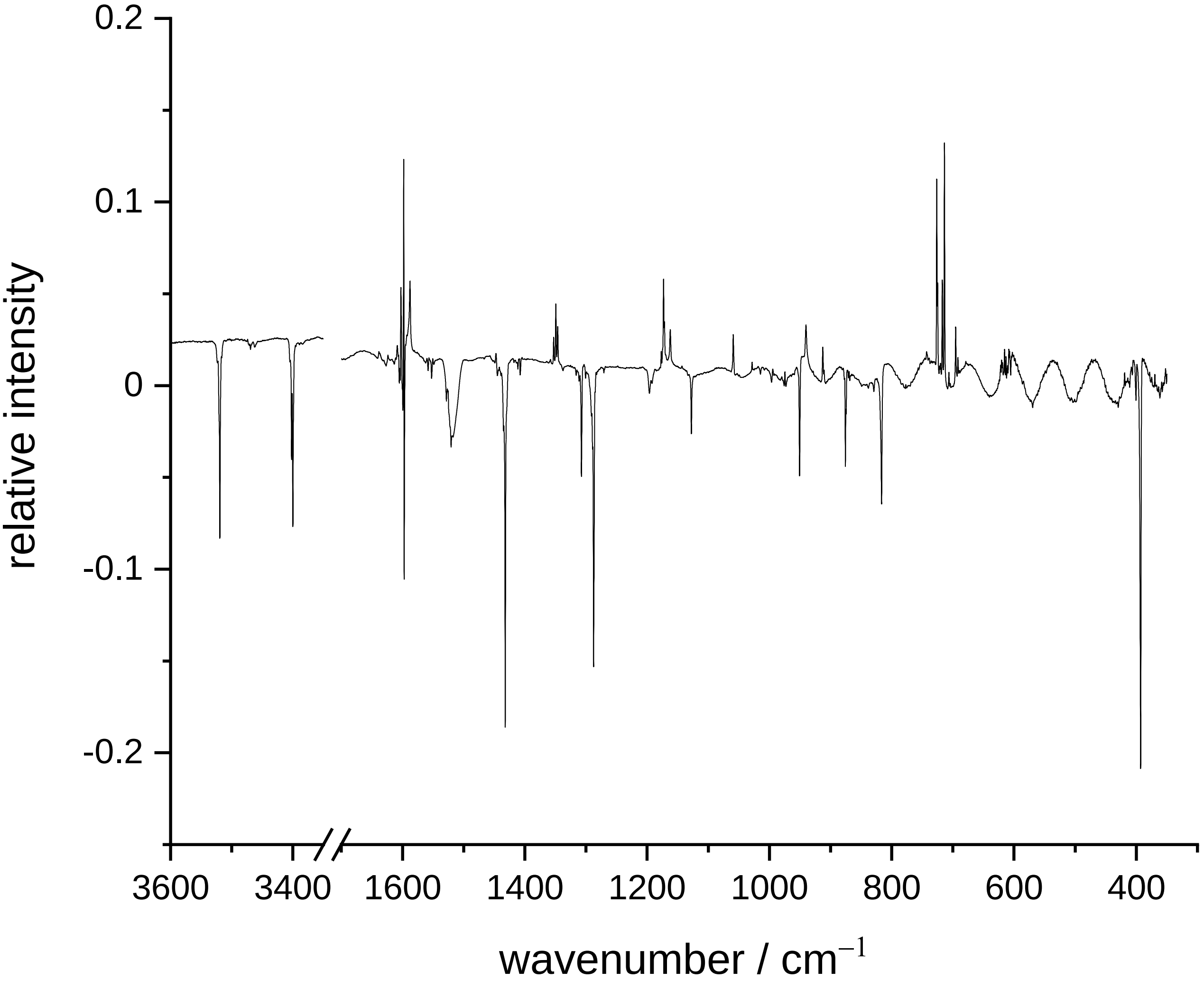

Figure S9. Difference spectrum of spectra measured before and after irradiation of **1** with > 320 nm for 10 min in an Ar matrix. Downward pointing bands belong to **1**, upward pointing bands belong to **2**.

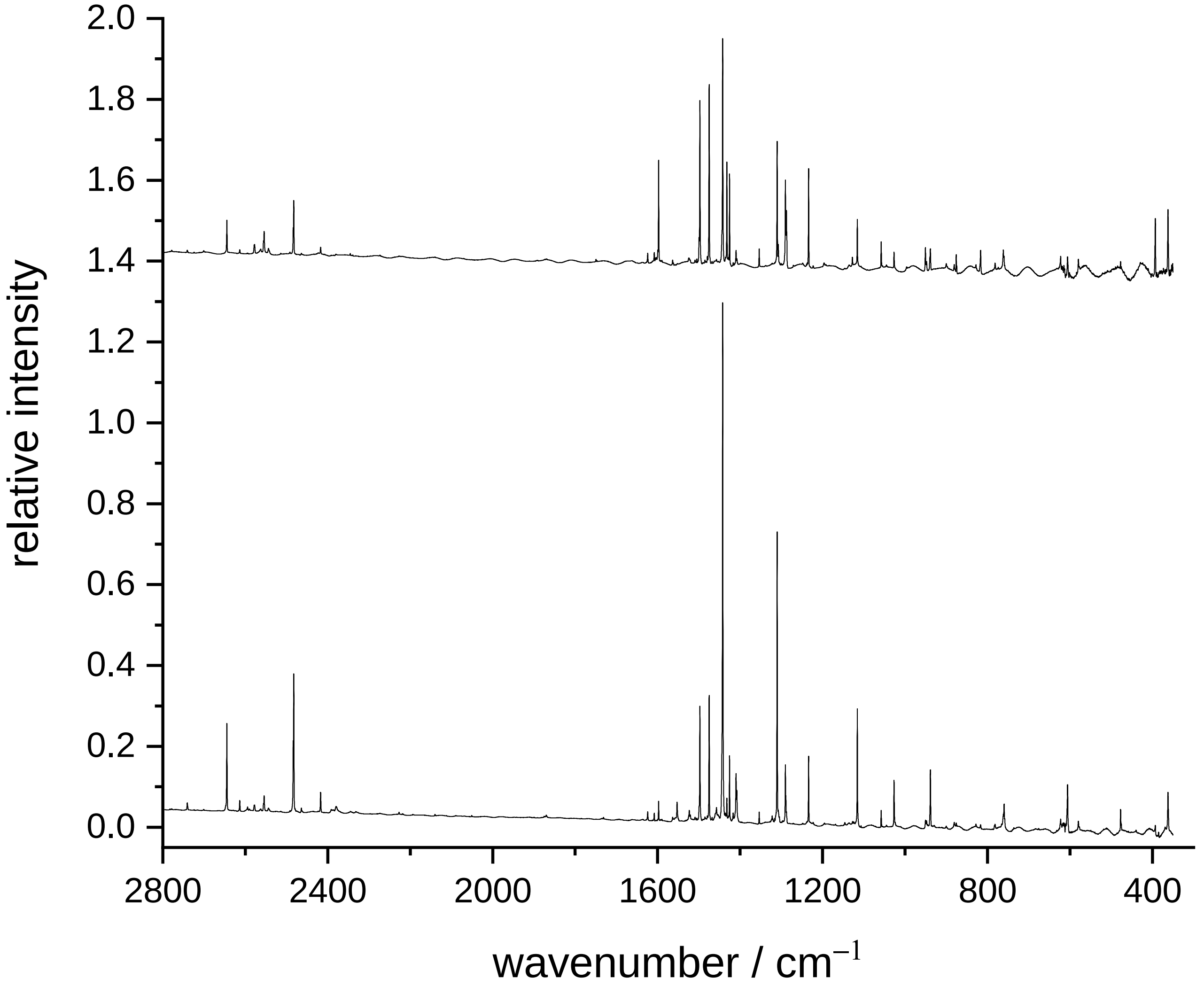

Figure S10. IR spectra of *N*-deuterated thioformamide in an Ar matrix. After synthesis, mixtures of three isotopologues (di- and monodeuterated forms) were obtained. Top: Higher concentration of the two *N*-monodeuterated thioformamide isotopomers. Bottom: Higher concentration of *N*,*N*-dideuterated thioformamide.

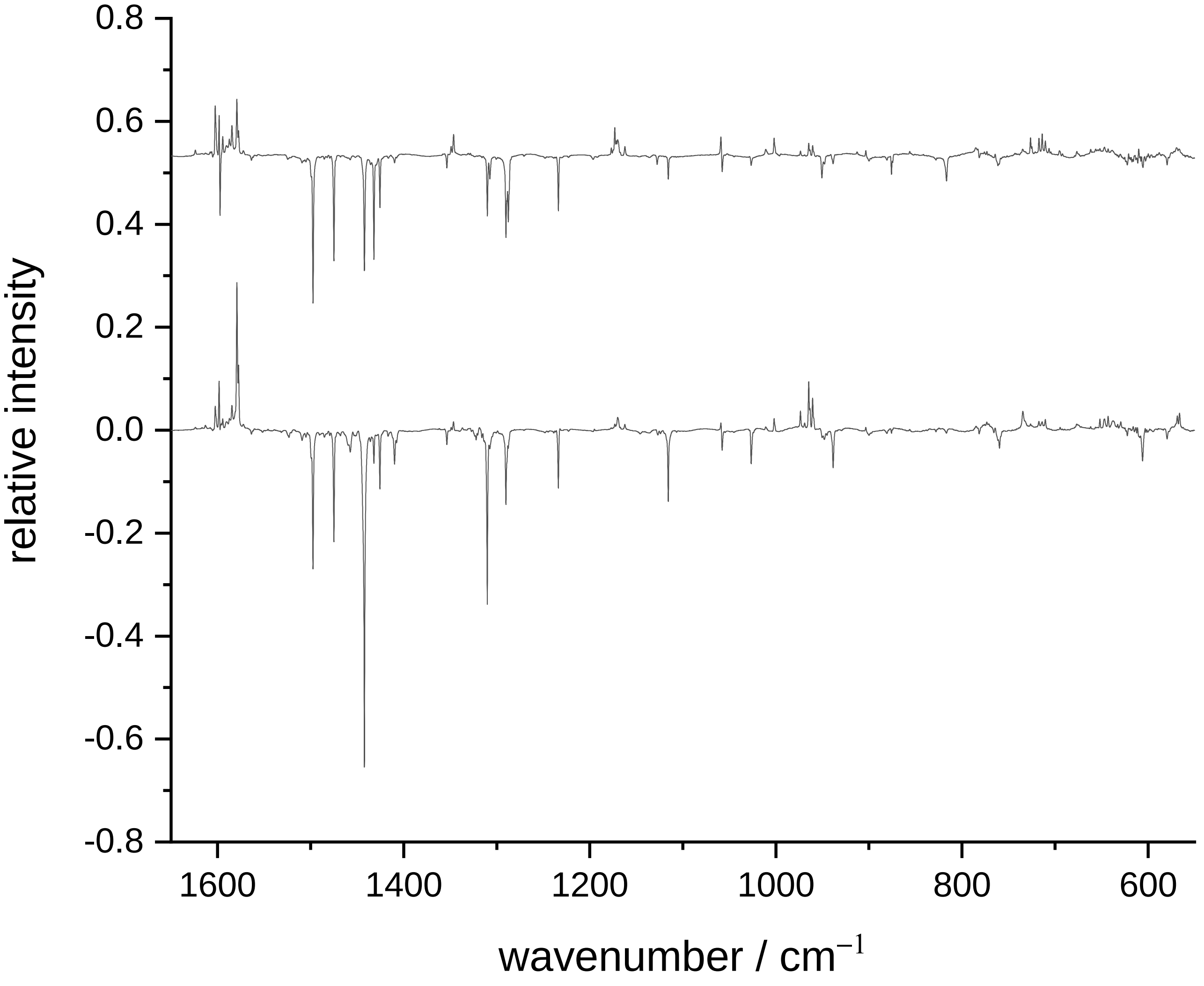

Figure S11. Difference IR spectra of spectra measured before and after irradiation with 254 nm for 10 min of the matrix using Ar as the host material. Downward pointing bands belong to isotopologues and isotopomers of *N*-deuterated thioformamide. Upward pointing bands belong to isotopologues and isotopomers of *N*,*S*-deuterated **2**. These bands are tentatively assigned to the four conformers of the dideuterated isotopologues based on computed anharmonic B3LYP/6-311++G(3df,3pd) frequencies. These bands remain unchanged after keeping the matrix for two days in the dark. Top: Higher concentration of the two *N*-monodeuterated thioformamide isotopomers. Bottom: Higher concentration of *N*,*N*-dideuterated thioformamide.

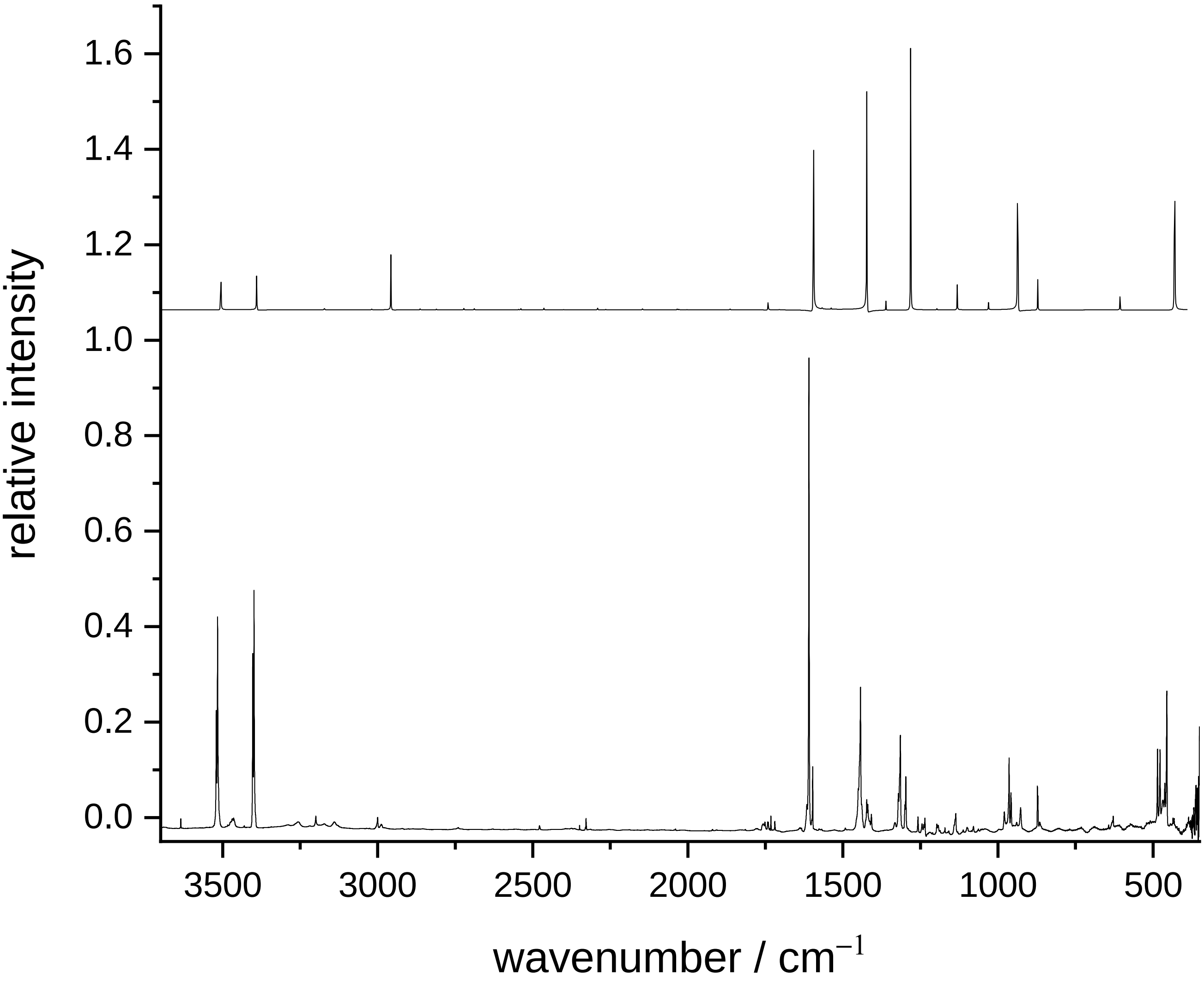

Figure S12. Bottom: IR spectrum of thioformamide (**1**) after deposition in an N_2 matrix at 3 K. Top: Computed anharmonic IR spectrum of **1** at B3LYP/6-311++G(3df,3pd).

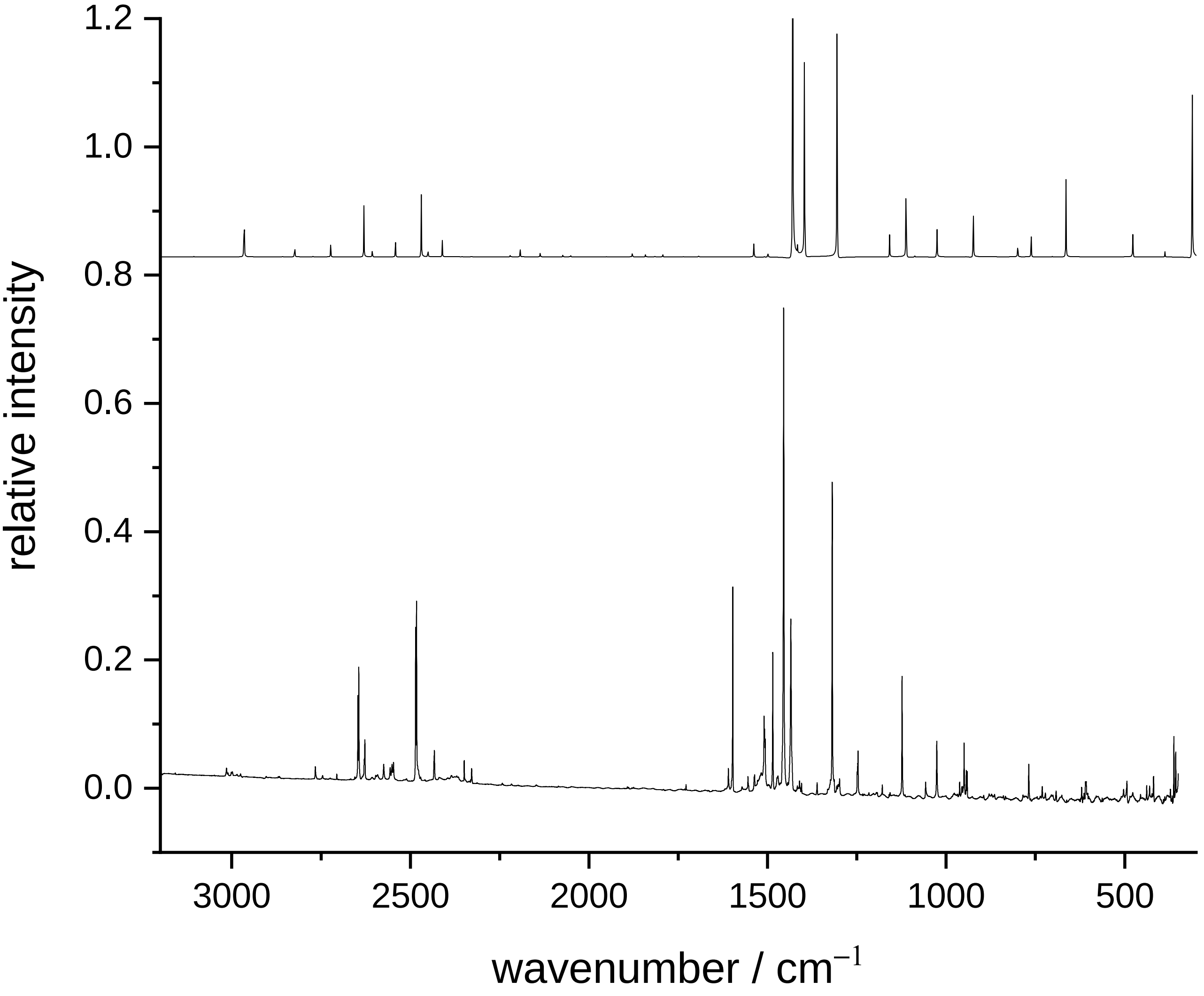

Figure S13. IR spectra of *N*-deuterated thioformamide in an N_2 matrix. Top: Computed anharmonic IR spectrum of *N*,*N*-dideuterated thioformamide at B3LYP/6-311++G(3df,3pd). Bottom: Experimental spectrum. *N*,*N*-dideuterated thioformamide was the main component after deposition but some bands of the un- and monodeuterated isotopologues are observable as well.

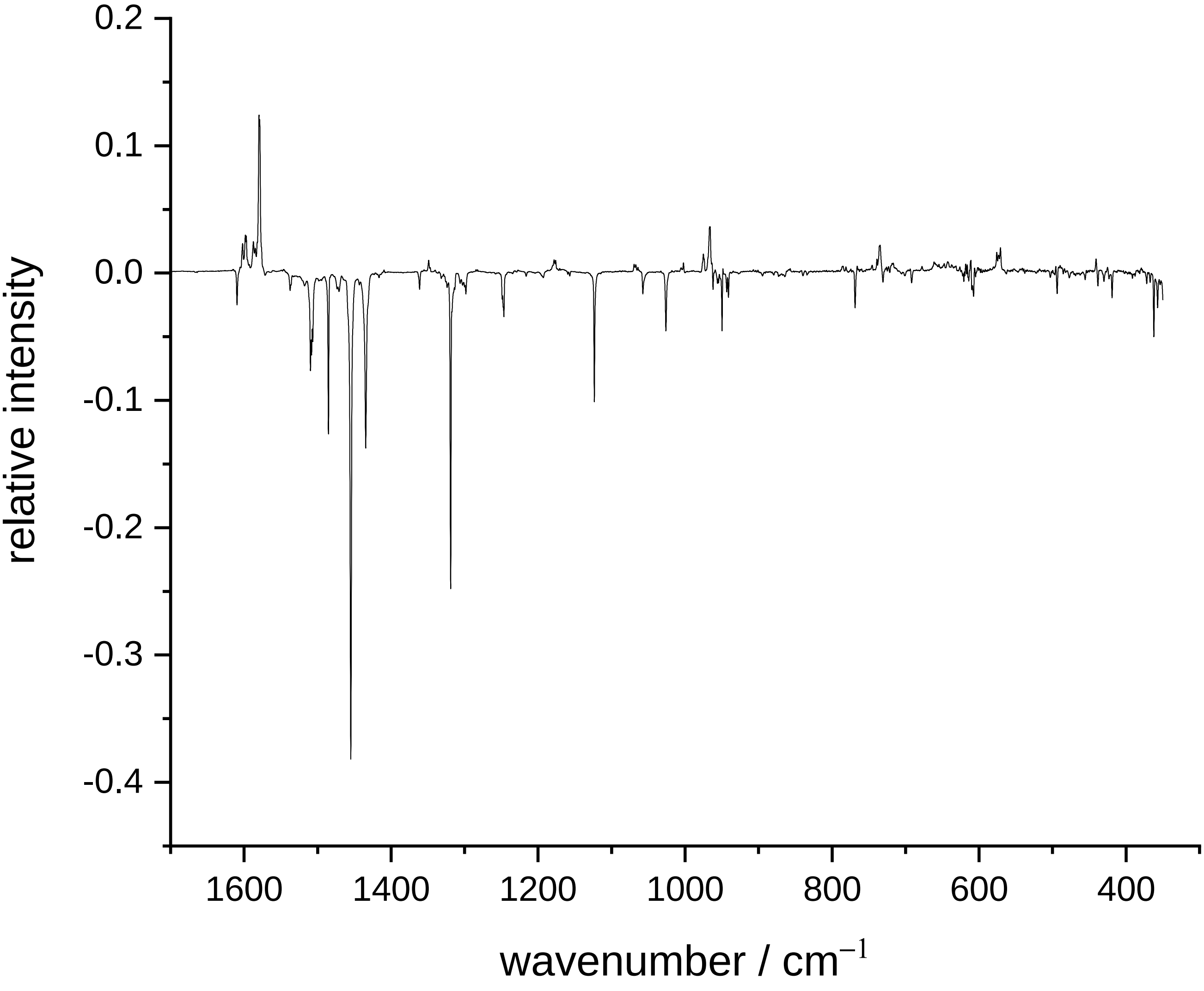

Figure S14. Difference spectrum of spectra measured before and after irradiation of a matrix using N_2 as the host with 254 nm for 10 min. Bands of *N*-deuterated thioformamide isotopologues and isotopomers are pointing downwards. Upward pointing bands can be assigned tentatively to the four *N*,*S*-dideuterated conformers of **2** based on anharmonic B3LYP/6-311++G(3df,3pd) computations. These bands remain unchanged after keeping the matrix for two days in the dark.

IR Spectroscopic Data

Table S11. Comparison of experimental vibrational frequencies of thioformamide **1** isolated in an Ar or N_2 matrix at 3 K and computed vibrational frequencies at the B3LYP/6-311++G(3df,3pd) level of theory (unscaled).

Assignment	Sym.	$\tilde{\nu}_{anharm}$ / cm^{-1}	I_{rel} / km mol^{-1}	$\tilde{\nu}_{harm}$ / cm^{-1}	I_{rel} / km mol^{-1}	$\tilde{\nu}(Ar)_{exp}$ / cm^{-1}	I_{rel}[a]	$\tilde{\nu}(N_2)_{exp}$ / cm^{-1}	I_{rel}[a]
$\rho_w(NH_2)$	*a*''	432.2	144.9	372.8	172.7	393.1	vs	455.6	m
δ(NCS)	*a*'	434.2	2.4	435.4	2.2	n.o.	n.o.	n.o.	n.o.
$\rho_t(NH_2)$	*a*''	607.9	7.6	628.1	3.6	605.4	vw	n.o.	n.o.
ν(C=S)	*a*'	873.2	16.9	885.5	16.7	875.7; 874.4; 816.8	m	873.0; 871.2	w
π(C-H)	*a*''	936.6	26.3	963.9	26.6	n.o.	n.o.	926.8	vw
2x $\rho_w(NH_2)$	*a*'	938.0	89.6	-	-	950.7	m	964.2	w
$\rho_r(NH_2)$	*a*'	1133.1	13.8	1145.1	20.7	1127.6	w	1136.1	w
ν(C-N)	*a*'	1283.3	144.4	1310.8	145.3	1287.6	s	1314.8; 1297.1	m
δ(C-H)	*a*'	1424.2	149.1	1460.2	172.6	1432.0	vs	1443.2; 1450.8	m
$\delta(NH_2)$	*a*'	1596.7	143.7	1637.1	173.9	1597.6	vs	1609.6	vs
ν(C-H)	*a*'	2958.5	30.9	3092.0	23.7	2996.5	w	3000.3	vw
ν_s(N-H)	*a*'	3391.1	25.9	3554.6	45.1	3400.1; 3402.2	m	3403.0; 3399.1	s
ν_a(N-H)	*a*'	3507.2	31.4	3688.1	42.0	3519.4	m	3520.8; 3516.6	s

[a] rel. experimental intensities (vw = very weak, w = weak, m = middle, s = strong, vs = very strong); n.o. = not observed; o.o.r. = out of range

Table S12. Comparison of experimental vibrational frequencies of *trans-cis*-thiolimine **2tc** isolated in an Ar or N_2 matrix at 3 K and computed vibrational frequencies at the B3LYP/6-311++G(3df,3pd) level of theory (unscaled).

Assignment	Sym.	$\tilde{\nu}_{anharm}$ / cm^{-1}	I_{rel} / km mol^{-1}	$\tilde{\nu}_{harm}$ / cm^{-1}	I_{rel} / km mol^{-1}	$\tilde{\nu}(Ar)_{exp}$ / cm^{-1}	I_{rel}[a]	$\tilde{\nu}(N_2)_{exp}$ / cm^{-1}	I_{rel}[a]
ρ_t(C-S)	*a*''	349.9	25.7	387.9	26.6	n.o.	n.o.	n.o.	n.o.
δ(NCS)	*a*'	417.1	14.0	425.0	16.2	n.o.	n.o.	n.o.	n.o.
ν(C-S)	*a*'	673.3	75.4	696.5	74.3	695.3; 691.8	m	n.o.	n.o.
ρ_t(N-H)	*a*''	720.0	73.5	729.7	76.2	717.2	m	720.2	s
δ(S-H)	*a*'	902.5	50.2	928.2	54.0	912.7; 910.7	m	911.4; 917.6	w
π(C-H)	*a*''	1026.5	2.2	1054.7	2.1	n.o.	n.o.	n.o.	n.o.
ρ_r(N-H)	*a*'	1166.7	33.5	1195.3	28.7	n.o.	n.o.	n.o.	n.o.
δ(C-H)	*a*'	1349.1	18.3	1380.6	22.1	1352.7	w	1353.9	m
ν(C=N)	*a*'	1628.6	177.8	1654.3	194.1	1587.8	s	1588.5	s
ν(S-H)	*a*'	2541.8	1.3	2668.2	1.8	n.o.	n.o.	n.o.	n.o.
ν(C-H)	*a*'	2876.1	31.8	3050.3	25.6	n.o.	n.o.	n.o.	n.o.
ν(N-H)	*a*'	3298.1	3.4	3474.2	6.1	n.o.	n.o.	n.o.	n.o.

[a] rel. experimental intensities (vw = very weak, w = weak, m = middle, s = strong, vs = very strong); n.o. = not observed; o.o.r. = out of range

Table S13. Comparison of experimental vibrational frequencies of *cis*-*cis*-thiolimine **2cc** isolated in an Ar or N_2 matrix at 3 K and computed vibrational frequencies at the B3LYP/6-311++G(3df,3pd) level of theory (unscaled).

Assignment	Sym.	$\tilde{\nu}_{\mathrm{anharm}}$ / cm^{-1}	I_{rel} / km mol^{-1}	$\tilde{\nu}_{\mathrm{harm}}$ / cm^{-1}	I_{rel} / km mol^{-1}	$\tilde{\nu}(\mathrm{Ar})_{\mathrm{exp}}$ / cm^{-1}	I_{rel}[a]	$\tilde{\nu}(\mathrm{N_2})_{\mathrm{exp}}$ / cm^{-1}	I_{rel}[a]
ρ_t(C-S)	*a*''	334.5	0.3	282.8	0.3	o.o.r.	o.o.r.	o.o.r.	o.o.r.
δ(NCS)	*a*'	409.7	3.0	413.3	3.8	n.o.	n.o.	n.o.	n.o.
ν(C-S)	*a*'	705.3	79.4	723.4	78.6	713.8	vs	723.2	s
ρ_t(N-H)	*a*''	718.0	68.7	724.6	77.7	726.3; 724.9	s	n.o.	n.o.
δ(S-H)	*a*'	944.2	52.7	969.5	57.3	940.0	m	942.0; 943.6<br>946.1	m
π(C-H)	*a*''	1024.7	2.8	1049.0	3.2	n.o.	n.o.	n.o.	n.o.
ρ_r(N-H)	*a*'	1149.7	38.9	1178.1	35.7	1162.2	m	1178.5	s
δ(C-H)	*a*'	1343.8	14.6	1374.7	16.2	1349.3	m	1349.9	s
ν(C=N)	*a*'	1632.5	216.4	1660.1	227.8	1598.0	s	1597.6	vs
ν(S-H)	*a*'	2576.3	0.3	2686.7	0.1	n.o.	n.o.	n.o.	n.o.
ν(C-H)	*a*'	2884.9	34.9	3053.3	26.8	n.o.	n.o.	n.o.	n.o.
ν(N-H)	*a*'	3308.9	6.4	3487.0	10.1	n.o.	n.o.	n.o.	n.o.

[a] rel. experimental intensities (vw = very weak, w = weak, m = middle, s = strong, vs = very strong); n.o. = not observed; o.o.r. = out of range

Table S14. Comparison of experimental vibrational frequencies of *cis*-*trans*-thiolimine **2ct** isolated in an Ar or N_2 matrix at 3 K and computed vibrational frequencies at the B3LYP/6-311++G(3df,3pd) level of theory (unscaled).

Assignment	Sym.	$\tilde{\nu}_{\mathrm{anharm}}$ / cm^{-1}	I_{rel} / km mol^{-1}	$\tilde{\nu}_{\mathrm{harm}}$ / cm^{-1}	I_{rel} / km mol^{-1}	$\tilde{\nu}(\mathrm{Ar})_{\mathrm{exp}}$ / cm^{-1}	I_{rel}[a]	$\tilde{\nu}(\mathrm{N_2})_{\mathrm{exp}}$ / cm^{-1}	I_{rel}[a]
ρ_t(C-S)	*a*''	312.2	16.0	234.9	18.8	o.o.r.	o.o.r.	o.o.r.	o.o.r.
δ(NCS)	*a*'	392.2	12.4	394.3	13.3	n.o.	n.o.	n.o.	n.o.
ν(C-S)	*a*'	681.1	35.7	698.2	35.4	n.o.	n.o.	n.o.	n.o.
ρ_t(N-H)	*a*''	751.0	8.5	761.0	8.2	706.4; 704.5	w	727.6	s
δ(S-H)	*a*'	914.5	2.2	937.4	2.9	n.o.	n.o.	n.o.	n.o.
π(C-H)	*a*''	1048.8	59.3	1076.6	59.5	1058.9	m	1067.2	m
ρ_r(N-H)	*a*'	1161.3	161.4	1194.8	149.4	1173.1; 1171.8	vs	1180.0	s
δ(C-H)	*a*'	1338.4	59.7	1373.2	66.5	1346.2	m	1346.7	m
ν(C=N)	*a*'	1639.6	181.6	1666.7	191.8	1602.5	s	1603.0	s
ν(S-H)	*a*'	2570.1	0.1	2682.4	0.0(2)	n.o.	n.o.	n.o.	n.o.
ν(C-H)	*a*'	2937.0	13.1	3115.5	9.1	n.o.	n.o.	n.o.	n.o.
ν(N-H)	*a*'	3269.4	4.8	3451.7	5.3	n.o.	n.o.	n.o.	n.o.

[a] rel. experimental intensities (vw = very weak, w = weak, m = middle, s = strong, vs = very strong); n.o. = not observed; o.o.r. = out of range

Table S15. Comparison of experimental vibrational frequencies of *trans-trans*-thiolimine **2tt** isolated in an Ar or N_2 matrix at 3 K and computed vibrational frequencies at the B3LYP/6-311++G(3df,3pd) level of theory (unscaled).

Assignment	Sym.	$\tilde{\nu}_{\mathrm{anharm}}$ / cm^{-1}	I_{rel} / km mol^{-1}	$\tilde{\nu}_{\mathrm{harm}}$ / cm^{-1}	I_{rel} / km mol^{-1}	$\tilde{\nu}(\mathrm{Ar})_{\mathrm{exp}}$ / cm^{-1}	I_{rel}[a]	$\tilde{\nu}(\mathrm{N_2})_{\mathrm{exp}}$ / cm^{-1}	I_{rel}[a]
ρ_t(C-S)	*a''*	321.5	5.6	324.4	5.4	o.o.r.	o.o.r.	o.o.r.	o.o.r.
δ(NCS)	*a'*	421.1	10.5	423.6	11.3	n.o.	n.o.	n.o.	n.o.
ν(C-S)	*a'*	660.4	56.9	667.7	55.4	n.o.	n.o.	n.o.	n.o.
ρ_t(N-H)	*a''*	750.3	8.5	760.5	11.4	n.o.	n.o.	726.0	m
δ(S-H)	*a'*	909.8	1.3	932.6	1.7	n.o.	n.o.	n.o.	n.o.
π(C-H)	*a''*	1048.3	57.3	1078.1	57.7	1059.9	w	1069.0	m
ρ_r(N-H)	*a'*	1161.9	183.5	1197.7	178.1	1176.9;	m	1182.8	w
δ(C-H)	*a'*	1352.2	25.3	1386.5	30.1	1358.0	w	1358.0	vw
ν(C=N)	*a'*	1638.3	170.2	1664.6	175.9	1606.2	w	1605.4	vw
ν(S-H)	*a'*	2549.7	0.8	2666.5	0.7	n.o.	n.o.	n.o.	n.o.
ν(C-H)	*a'*	2929.0	11.4	3102.7	8.5	n.o.	n.o.	n.o.	n.o.
ν(N-H)	*a'*	3276.6	6.3	3458.8	6.5	n.o.	n.o.	n.o.	n.o.

[a] rel. experimental intensities (vw = very weak, w = weak, m = middle, s = strong, vs = very strong); n.o. = not observed; o.o.r. = out of range

Table S16. Comparison of experimental vibrational frequencies of *N*,*N*-dideuterated thioformamide isolated in an Ar or N_2 matrix at 3 K and computed vibrational frequencies at the B3LYP/6-311++G(3df,3pd) levels of theory (unscaled).

Assignment	Sym.	$\tilde{\nu}_{\mathrm{anharm}}$ / cm^{-1}	I_{rel} / km mol^{-1}	$\tilde{\nu}_{\mathrm{harm}}$ / cm^{-1}	I_{rel} / km mol^{-1}	$\tilde{\nu}(\mathrm{Ar})_{\mathrm{exp}}$ / cm^{-1}	I_{rel}[a]	$\tilde{\nu}(\mathrm{N_2})_{\mathrm{exp}}$ / cm^{-1}	I_{rel}[a]
ρ_w(ND_2)	*a''*	312.6	75.9	283.2	87.2	o.o.r.	o.o.r.	o.o.r.	o.o.r.
δ(NCS)	*a'*	389.1	2.4	390.5	1.8	n.o.	n.o.	n.o.	n.o.
ρ_t(ND_2)	*a''*	478.3	11.9	490.4	8.7	477.1; 475.5	w	n.o.	n.o.
2x ρ_w(ND_2)	*a'*	665.9	31.0	-	-	605.9	m	n.o.	n.o.
ν(C=S)	*a'*	762.9	8.1	778.3	6.8	759.7; 761.1; 758.2	w	768.7	m
π(C-H)	*a''*	925.5	20.0	949.0	20.4	938.5; 940.0	m	949.7	m
ρ_r(ND_2)	*a'*	1027.2	14.5	1137.3	16.9	1026.6	m	1026.1	m
δ(ND_2)	*a'*	1113.2	34.6	1338.2	98.0	1115.7	s	1123.5	s
ν(C-N)	*a'*	1306.4	100.2	1338.2	98.0	1310.2	vs	1319.0	vs
π(C-H) + ρ_t(ND_2)	*a'*	1397.6	36.0	-	-	1409.9; 1407.8	m	n.o.	n.o.
δ(C-H)	*a'*	1430.9	266.7	1468.9	334.8	1442.2	vs	1454.9	vs
ν_s(N-D)	*a'*	2470.7	26.5	2566.8	49.5	2482.5; 2483.8	s	2482.7; 2485.3	s
ν_a(N-D)	*a'*	2631.1	20.5	2732.1	24.6	2644.7	s	2644.4; 2646.8	m
ν(C-H)	*a'*	2966.2	24.5	3092.3	21.8	3007.4	w	n.o.	n.o.

[a] rel. experimental intensities (vw = very weak, w = weak, m = middle, s = strong, vs = very strong); n.o. = not observed; o.o.r. = out of range

Table S17. Comparison of experimental vibrational frequencies of *N*-monodeuterated thioformamide (a) isolated in an Ar or N_2 matrix at 3 K and computed vibrational frequencies at the B3LYP/6-311++G(3df,3pd) levels of theory (unscaled).

Assignment	Sym.	$\tilde{\nu}_{anharm}$ / cm^{-1}	I_{rel} / km mol^{-1}	$\tilde{\nu}_{harm}$ / cm^{-1}	I_{rel} / km mol^{-1}	$\tilde{\nu}(Ar)_{exp}$ / cm^{-1}	I_{rel}[a]	$\tilde{\nu}(N_2)_{exp}$ / cm^{-1}	I_{rel}[a]
ρ_w(NDH)	*a''*	393.7	128.9	346.3	146.8	362.1	m	n.o.	n.o.
δ(NCS)	*a'*	399.2	2.5	400.7	1.9	n.o.	n.o.	n.o.	n.o.
ρ_t(NDH)	*a''*	520.4	4.0	540.7	5.3	n.o.	n.o.	n.o.	n.o.
ν(C=S)	*a'*	830.4	6.3	850.1	11.9	n.o.	n.o.	n.o.	n.o.
2x ρ_w(NDH)	*a'*	857.4	63.8	-	-	828.0	vw	n.o.	n.o.
π(C-H)	*a''*	927.4	15.2	950.3	16.8	900.0	w	941.0	w
ρ_r(NDH)	*a'*	1029.4	3.4	1039.4	13.6	n.o.	n.o.	n.o.	n.o.
ν(C-N)	*a'*	1228.3	95.6	1261.0	105.6	1233.8	s	1246.5	w
δ(C-H)	*a'*	1416.0	157.0	1459.2	148.0	1425.6	s	n.o.	n.o.
δ(NDH)	*a'*	1471.2	157.6	1508.3	231.2	1475.0	vs	1434.4	s
ν(N-D)	*a'*	2540.2	20.0	2635.3	29.7	n.o.	n.o.	n.o.	n.o.
ν(C-H)	*a'*	2965.9	26.0	3092.2	23.4	n.o.	n.o.	n.o.	n.o.
ν(N-H)	*a'*	3462.3	29.9	3637.8	47.7	3471.6	m	3470.5; 3473.6	m

[a] rel. experimental intensities (vw = very weak, w = weak, m = middle, s = strong, vs = very strong); n.o. = not observed; o.o.r. = out of range

Table S18. Comparison of experimental vibrational frequencies of *N*-monodeuterated thioformamide (b) isolated in an Ar or N_2 matrix at 3 K and computed vibrational frequencies at the B3LYP/6-311++G(3df,3pd) levels of theory (unscaled).

Assignment	Sym.	$\tilde{\nu}_{anharm}$ / cm^{-1}	I_{rel} / km mol^{-1}	$\tilde{\nu}_{harm}$ / cm^{-1}	I_{rel} / km mol^{-1}	$\tilde{\nu}(Ar)_{exp}$ / cm^{-1}	I_{rel}[a]	$\tilde{\nu}(N_2)_{exp}$ / cm^{-1}	I_{rel}[a]
ρ_w(NDH)	*a''*	324.0	71.9	290.1	87.8	o.o.r.	o.o.r.	o.o.r.	o.o.r.
δ(NCS)	*a'*	420.5	2.1	421.4	1.9	n.o.	n.o.	n.o.	n.o.
ρ_t(NDH)	*a''*	586.7	35.6	599.5	30.5	579.6; 577.1	w	n.o.	n.o.
2x ρ_w(NDH)	*a'*	683.7	32.3	-	-	622.5	w	n.o.	n.o.
ν(C=S)	*a'*	779.1	9.7	793.2	7.3	n.o.	n.o.	n.o.	n.o.
π(C-H)	*a''*	935.0	30.2	961.8	32.1	947.6	m	962.0	w
ρ_r(NDH)	*a'*	1058.6	30.2	1072.0	32.2	1057.8	m	1057.5	w
ν(C-N)	*a'*	1283.2	120.1	1308.5	142.9	1290.1	s	1298.2	w
δ(C-H)	*a'*	1350.6	26.1	1380.0	28.0	n.o.	n.o.	1361.2	w
δ(NDH)	*a'*	1491.7	227.0	1536.6	298.9	1497.5; 1499.5	vs	1485.3	s
ν(N-D)	*a'*	2560.6	27.4	2653.2	46.0	n.o.	n.o.	n.o.	n.o.
ν(C-H)	*a'*	2941.2	23.0	3092.1	22.7	2970.2	vw	n.o.	n.o.
ν(N-H)	*a'*	3438.8	27.3	3615.5	37.7	3456.7	m	3451.3; 3456.2	m

[a] rel. experimental intensities (vw = very weak, w = weak, m = middle, s = strong, vs = very strong); n.o. = not observed; o.o.r. = out of range

Table S19. Comparison of experimental vibrational frequencies of *N*,*S*-dideuterated *trans*-*cis*-thiolimine **2tc** isolated in an Ar or N_2 matrix at 3 K and computed vibrational frequencies at the B3LYP/6-311++G(3df,3pd) levels of theory (unscaled).

Assignment	Sym.	$\tilde{\nu}_{\mathrm{anharm}}$ / cm^{-1}	I_{rel} / km mol^{-1}	$\tilde{\nu}_{\mathrm{harm}}$ / cm^{-1}	I_{rel} / km mol^{-1}	$\tilde{\nu}(\mathrm{Ar})_{\mathrm{exp}}$ / cm^{-1}	I_{rel}[a]	$\tilde{\nu}(\mathrm{N_2})_{\mathrm{exp}}$ / cm^{-1}	I_{rel}[a]
ρ_t(C-S)	*a''*	291.0	23.4	305.1	24.2	o.o.r.	o.o.r.	o.o.r.	o.o.r.
δ(NCS)	*a'*	378.1	13.0	381.9	14.6	n.o.	n.o.	n.o.	n.o.
ρ_t(N-D)	*a''*	568.3	40.9	575.2	41.9	568.6	m	575.9	m
ν(C-S)	*a'*	632.5	34.4	648.4	35.7	643.0	m	n.o.	n.o.
δ(S-D)	*a'*	702.5	55.5	714.9	59.0	710.4	w	n.o.	n.o.
ρ_r(N-D)	*a'*	960.4	20.8	981.2	20.2	973.6	m	n.o.	n.o.
π(C-H)	*a''*	972.5	3.0	991.4	3.2	n.o.	n.o.	n.o.	n.o.
δ(C-H)	*a'*	1322.9	3.2	1358.0	6.7	n.o.	n.o.	n.o.	n.o.
ν(C-S) + ρ_r(N-D)	*a'*	1585.4	14.1	-	-	n.o.	n.o.	n.o.	n.o.
ν(C=N)	*a'*	1611.1	187.0	1637.7	208.8	1577.5	s	1578.7	vs
ν(S-D)	*a'*	1852.5	0.7	1915.6	0.8	n.o.	n.o.	n.o.	n.o.
ν(N-D)	*a'*	2442.2	8.1	2541.9	12.3	n.o.	n.o.	n.o.	n.o.
ν(C-H)	*a'*	2864.2	23.0	3051.3	26.0	n.o.	n.o.	n.o.	n.o.

[a] rel. experimental intensities (vw = very weak, w = weak, m = middle, s = strong, vs = very strong); n.o. = not observed; o.o.r. = out of range

Table S20. Comparison of experimental vibrational frequencies of *N*,*S*-dideuterated *cis*-*cis*-thiolimine **2cc** isolated in an Ar or N_2 matrix at 3 K and computed vibrational frequencies at the B3LYP/6-311++G(3df,3pd) levels of theory (unscaled).

Assignment	Sym.	$\tilde{\nu}_{\mathrm{anharm}}$ / cm^{-1}	I_{rel} / km mol^{-1}	$\tilde{\nu}_{\mathrm{harm}}$ / cm^{-1}	I_{rel} / km mol^{-1}	$\tilde{\nu}(\mathrm{Ar})_{\mathrm{exp}}$ / cm^{-1}	I_{rel}[a]	$\tilde{\nu}(\mathrm{N_2})_{\mathrm{exp}}$ / cm^{-1}	I_{rel}[a]
ρ_t(C-S)	*a''*	234.7	0.6	218.5	0.8	o.o.r.	o.o.r.	o.o.r.	o.o.r.
δ(NCS)	*a'*	387.9	3.9	391.0	4.6	n.o.	n.o.	n.o.	n.o.
ρ_t(N-D)	*a''*	564.3	40.5	572.4	43.6	566.2	m	570.9	m
ν(C-S)	*a'*	629.1	33.1	643.2	31.8	637.6	m	n.o.	n.o.
δ(S-D)	*a'*	722.8	64.5	738.4	67.4	734.6	m	734.6	m
ρ_r(N-D)	*a'*	958.7	15.2	979.9	13.8	969.1	s	975.2	m
π(C-H)	*a''*	964.2	2.8	981.7	2.8	n.o.	n.o.	n.o.	n.o.
δ(C-H)	*a'*	1318.9	6.3	1350.3	9.2	1349.0	w	1348.9	w
ν(C-S) + ρ_r(N-D)	*a'*	1580.1	21.7	-	-	n.o.	n.o.	n.o.	n.o.
ν(C=N)	*a'*	1616.5	211.9	1643.4	244.4	1579.3	vs	1579.7	vs
ν(S-D)	*a'*	1871.1	0.2	1929.4	0.2	n.o.	n.o.	n.o.	n.o.
ν(N-D)	*a'*	2451.2	11.2	2551.7	16.5	n.o.	n.o.	n.o.	n.o.
ν(C-H)	*a'*	2871.9	23.9	3054.5	27.0	n.o.	n.o.	n.o.	n.o.
δ(C-H) + *ν*(C=N)	*a'*	2968.2	11.2	-	-	n.o.	n.o.	n.o.	n.o.

[a] rel. experimental intensities (vw = very weak, w = weak, m = middle, s = strong, vs = very strong); n.o. = not observed; o.o.r. = out of range

Table S21. Comparison of experimental vibrational frequencies of *N*,*S*-dideuterated *cis-trans*-thiolimine **2ct** isolated in an Ar or N_2 matrix at 3 K and computed vibrational frequencies at the B3LYP/6-311++G(3df,3pd) levels of theory (unscaled).

Assignment	Sym.	$\tilde{\nu}_{anharm}$ / cm^{-1}	I_{rel} / km mol^{-1}	$\tilde{\nu}_{harm}$ / cm^{-1}	I_{rel} / km mol^{-1}	$\tilde{\nu}(Ar)_{exp}$ / cm^{-1}	I_{rel}[a]	$\tilde{\nu}(N_2)_{exp}$ / cm^{-1}	I_{rel}[a]
ρ_t(C-S)	*a''*	205.4	8.5	174.9	9.1	o.o.r.	o.o.r.	o.o.r.	o.o.r.
δ(NCS)	*a'*	359.2	13.2	360.5	12.9	n.o.	n.o.	n.o.	n.o.
ρ_t(N-D)	*a'*	616.7	17.7	630.1	16.7	629.3	m	n.o.	n.o.
ν(C-S)	*a''*	660.9	16.4	671.1	15.9	651.8	m	n.o.	n.o.
δ(S-D)	*a'*	715.4	7.8	731.4	8.3	n.o.	n.o.	n.o.	n.o.
ρ_r(N-D)	*a'*	950.1	114.0	973.3	112.4	964.7; 963.4	s	965.9	s
π(C-H)	*a''*	952.7	28.9	971.7	29.2	n.o.	n.o.	n.o.	n.o.
δ(C-H)	*a'*	1336.3	10.0	1353.7	20.3	1346.4	w	1346.8	vw
ρ_t(N-D) + ρ_r(N-D)	*a'*	1560.9	11.6	-	-	n.o.	n.o.	n.o.	n.o.
ν(C-S) + π(C-H)	*a'*	1608.8	65.3	-	-	n.o.	n.o.	n.o.	n.o.
ν(C=N)	*a'*	1624.6	146.6	1649.0	226.6	1598.4	s	1597.0	m
ν(S-D)	*a'*	1867.4	0.1	1926.5	0.0(01)	n.o.	n.o.	n.o.	n.o.
ν(N-D)	*a'*	2422.0	5.1	2527.6	8.5	n.o.	n.o.	n.o.	n.o.
ν(C-H)	*a'*	3000.9	8.7	3115.5	9.2	n.o.	n.o.	n.o.	n.o.

[a] rel. experimental intensities (vw = very weak, w = weak, m = middle, s = strong, vs = very strong); n.o. = not observed; o.o.r. = out of range

Table S22. Comparison of experimental vibrational frequencies of *N*,*S*-dideuterated *trans-trans*-thiolimine **2tt** isolated in an Ar or N_2 matrix at 3 K and computed vibrational frequencies at the B3LYP/6-311++G(3df,3pd) levels of theory (unscaled).

Assignment	Sym.	$\tilde{\nu}_{anharm}$ / cm^{-1}	I_{rel} / km mol^{-1}	$\tilde{\nu}_{harm}$ / cm^{-1}	I_{rel} / km mol^{-1}	$\tilde{\nu}(Ar)_{exp}$ / cm^{-1}	I_{rel}[a]	$\tilde{\nu}(N_2)_{exp}$ / cm^{-1}	I_{rel}[a]
ρ_t(C-S)	*a''*	241.2	1.7	246.4	1.6	o.o.r.	o.o.r.	o.o.r.	o.o.r.
δ(NCS)	*a'*	371.3	10.6	373.6	11.0	n.o.	n.o.	n.o.	n.o.
ρ_t(N-D)	*a'*	616.0	33.9	631.4	33.4	n.o.	n.o.	n.o.	n.o.
ν(C-S)	*a''*	654.5	17.9	664.5	17.7	646.9	m	n.o.	n.o.
δ(S-D)	*a'*	704.1	7.7	719.2	8.5	n.o.	n.o.	n.o.	n.o.
ρ_r(N-D)	*a'*	946.1	125.0	969.5	123.2	960.5; 959.2	m	n.o.	n.o.
π(C-H)	*a''*	961.4	26.9	982.6	27.3	n.o.	n.o.	n.o.	n.o.
δ(C-H)	*a'*	1345.8	7.1	1367.8	8.3	n.o.	n.o.	n.o.	n.o.
ρ_t(N-D) + ρ_r(N-D)	*a'*	1556.1	10.8	-	-	n.o.	n.o.	n.o.	n.o.
ν(C-S) + π(C-H)	*a'*	1611.3	27.1	-	-	n.o.	n.o.	n.o.	n.o.
ν(C=N)	*a'*	1623.3	162.3	1647.8	206.5	1584.6	m	1584.9	m
ν(S-D)	*a'*	1854.1	0.6	1914.8	0.6	n.o.	n.o.	n.o.	n.o.
ν(N-D)	*a'*	2425.9	5.3	2532.4	9.1	n.o.	n.o.	n.o.	n.o.
ν(C-H)	*a'*	2916.9	6.3	3102.6	8.5	n.o.	n.o.	n.o.	n.o.

Matrix-UV/Vis Spectrum in N_2

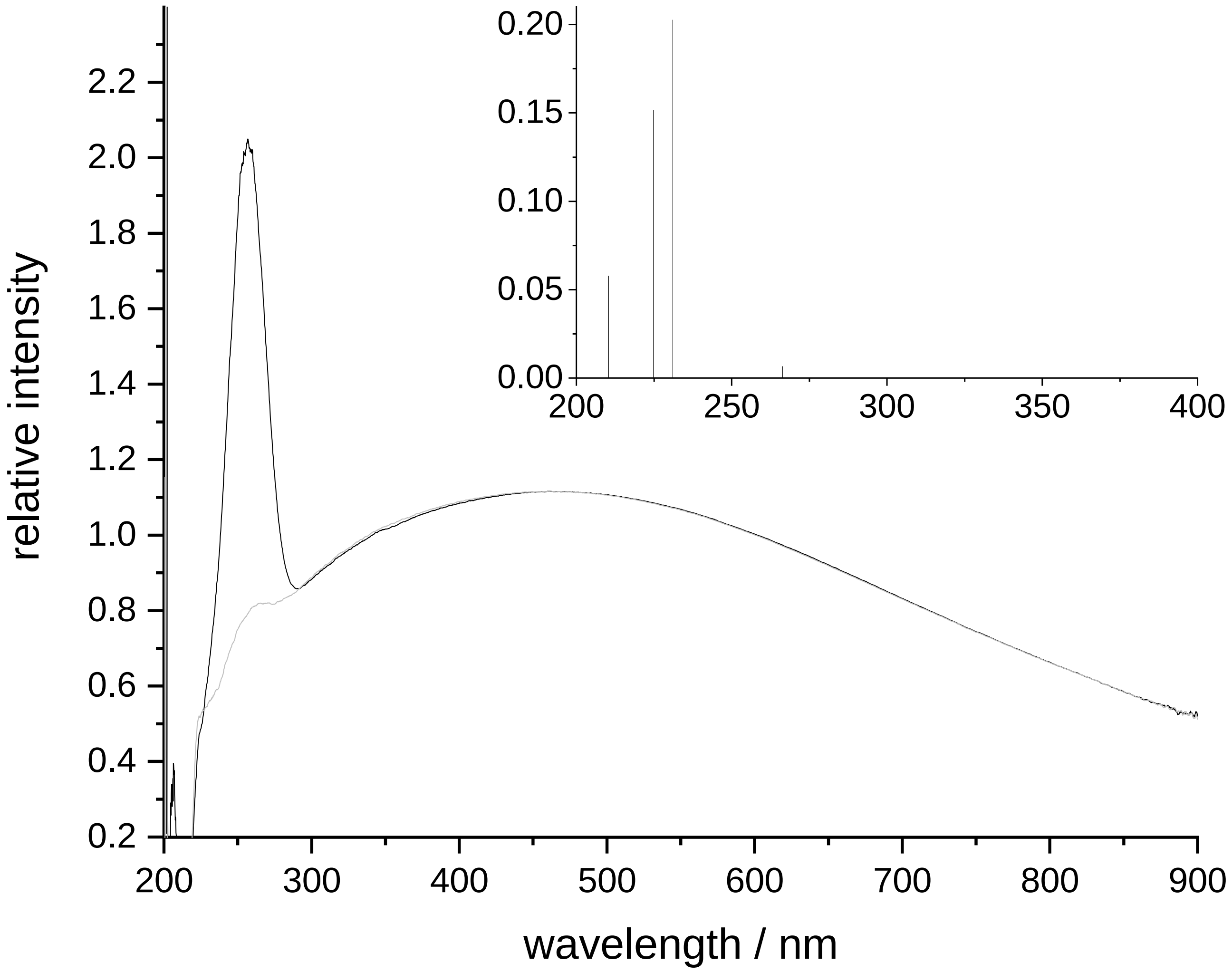

Figure S15. UV/Vis spectrum of thioformamide in an N_2 matrix. Black: After deposition. Grey: After irradiation with 254 nm for 10 min. Inset: Computed spectrum of **1** at the TD-B3LYP/6-311++G(3df,3pd) level of theory.

UV/Vis Spectroscopic Data

Table S23. TD-DFT B3LYP/6-311++G(3df,3pd) computed vertical excitation energies of thioformamide **1** and comparison with the experimental UV/Vis spectra in Ar and N_2.

Excitation Energy λ / nm	Oscillator Strength (*f*)	$\lambda(Ar)_{exp.}$ / nm	$\lambda(N_2)_{exp.}$ / nm	Transition
373.91	0.0001	n.o.	n.o.	HOMO – LUMO
266.42	0.0067	n.o.	n.o.	HOMO−1 – LUMO (16%)<br>HOMO – LUMO+1 (84%)
234.55	0.0001	n.o.	n.o.	HOMO−1 – LUMO+1
231.00	0.2027	256	255	HOMO−1 – LUMO (44%)<br>HOMO – LUMO+1 (9%)<br>HOMO – LUMO+2 (47%)
224.91	0.1517	n.o.	n.o.	HOMO−1 – LUMO (37%)<br>HOMO – LUMO+1 (8%)<br>HOMO – LUMO+2 (55%)
210.33	0.0578	n.o.	n.o.	HOMO – LUMO+3

n.o. = not observed; o.o.r. = out of range

Table S24. TD-DFT B3LYP/6-311++G(3df,3pd) computed vertical excitation energies of *trans-cis*-thiolimine **2tc** and comparison with the experimental UV/Vis spectra in Ar and N_2.

Excitation Energy λ / nm	Oscillator Strength (*f*)	$\lambda(Ar)_{exp.}$ / nm	$\lambda(N_2)_{exp.}$ / nm	Transition
288.14	0.0029	n.o.	n.o.	HOMO−1 – LUMO
221.45	0.0028	n.o.	n.o.	HOMO – LUMO+1 (74%)<br>HOMO – LUMO+2 (23%)<br>HOMO – LUMO+3 (3%)
212.14	0.1426	225	n.o.	HOMO−1 – LUMO+3 (6%)<br>HOMO – LUMO (94%)
200.47	0.0003	n.o.	n.o.	HOMO – LUMO+1 (27%)<br>HOMO – LUMO+2 (69%)<br>HOMO – LUMO+3 (4%)
194.57	0.0188	n.o.	n.o.	HOMO – LUMO+2 (7%)<br>HOMO – LUMO+3 (93%)
191.25	0.0052	n.o.	n.o.	HOMO−1 – LUMO+1

n.o. = not observed; o.o.r. = out of range

Table S25. TD-DFT B3LYP/6-311++G(3df,3pd) computed vertical excitation energies of *cis-cis*-thiolimine **2cc** and comparison with the experimental UV/Vis spectra in Ar and N_2.

Excitation Energy λ / nm	Oscillator Strength (f)	$\lambda(Ar)_{exp.}$ / nm	$\lambda(N_2)_{exp.}$ / nm	Transition
233.37	0.0009	n.o.	n.o.	HOMO−1 – LUMO (8%)<br>HOMO – LUMO+1 (88%)<br>HOMO – LUMO+3 (4%)
228.72	0.0045	n.o.	n.o.	HOMO−1 – LUMO (93%)<br>HOMO – LUMO+1 (7%)
211.03	0.1380	225	n.o.	HOMO−1 – LUMO+2 (6%)<br>HOMO – LUMO (94%)
203.34	0.0073	n.o.	n.o.	HOMO−1 – LUMO+1
198.66	0.0108	n.o.	n.o.	HOMO – LUMO+1 (6%)<br>HOMO – LUMO+2 (67%)<br>HOMO – LUMO+3 (27%)
189.87	0.0175	o.o.r.	o.o.r.	HOMO – LUMO+2 (32%)<br>HOMO – LUMO+3 (68%)

n.o. = not observed; o.o.r. = out of range

Table S26. TD-DFT B3LYP/6-311++G(3df,3pd) computed vertical excitation energies of *cis-trans*-thiolimine **2ct** and comparison with the experimental UV/Vis spectra in Ar and N_2.

Excitation Energy λ / nm	Oscillator Strength (f)	$\lambda(Ar)_{exp.}$ / nm	$\lambda(N_2)_{exp.}$ / nm	Transition
234.73	0.0015	n.o.	n.o.	HOMO – LUMO+1 (95%)<br>HOMO – LUMO+2 (5%)
229.70	0.0024	n.o.	n.o.	HOMO−1 – LUMO
211.98	0.0640	n.o.	n.o.	HOMO−1 – LUMO+1 (23%)<br>HOMO−1 – LUMO+2 (10%)<br>HOMO – LUMO (67%)
204.93	0.0238	n.o.	n.o.	HOMO−1 – LUMO+1 (78%)<br>HOMO – LUMO (22%)
197.99	0.0178	n.o.	n.o.	HOMO – LUMO+1 (5%)<br>HOMO – LUMO+2 (95%)
182.98	0.0003	o.o.r.	o.o.r.	HOMO – LUMO+3

n.o. = not observed; o.o.r. = out of range

Table S27. TD-DFT B3LYP/6-311++G(3df,3pd) computed vertical excitation energies of *trans-trans*-thiolimine **2tt** and comparison with the experimental UV/Vis spectra in Ar and N_2.

Excitation Energy λ / nm	Oscillator Strength (f)	$\lambda(Ar)_{exp.}$ / nm	$\lambda(N_2)_{exp.}$ / nm	Transition
232.80	0.0012	n.o.	n.o.	HOMO−1 – LUMO
228.03	0.0037	n.o.	n.o.	HOMO – LUMO+1 (94%)<br>HOMO – LUMO+2 (6%)
213.67	0.0559	n.o.	n.o.	HOMO−1 – LUMO+1 (22%)<br>HOMO−1 – LUMO+2 (10%)<br>HOMO – LUMO (68%)
202.31	0.0254	n.o.	n.o.	HOMO−1 – LUMO+1 (78%)<br>HOMO−1 – LUMO+2 (4%)<br>HOMO – LUMO (18%)
197.84	0.0112	n.o.	n.o.	HOMO – LUMO+1 (7%)<br>HOMO – LUMO+2 (93%)
181.90	0.0078	o.o.r.	o.o.r.	HOMO – LUMO+2 (3%)<br>HOMO – LUMO+3 (94%)<br>HOMO – LUMO+4 (3%)

n.o. = not observed; o.o.r. = out of range

Natural Bond Orbital Analysis

All natural bond orbitals (NBOs) were computed at the HF/6-311++G(2d,2p) level of theory based on the AE-CCSD(T)/aug-cc-pCVTZ optimized structure of thioformamide (**1**). HOMO = highest occupied molecular orbital. LUMO = lowest unoccupied molecular orbital. Color code: Carbon – black, hydrogen – white, nitrogen – light blue, sulfur – yellow. Opposing signs of the displayed NBOs are represented in red and dark blue color, respectively.

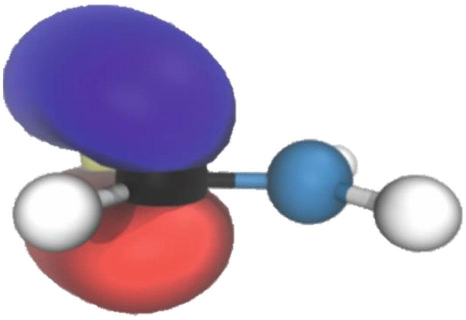

Figure S16. The HOMO−1 NBO (A'') of thioformamide (**1**) is a π-type orbital responsible for the C=S π-bond.

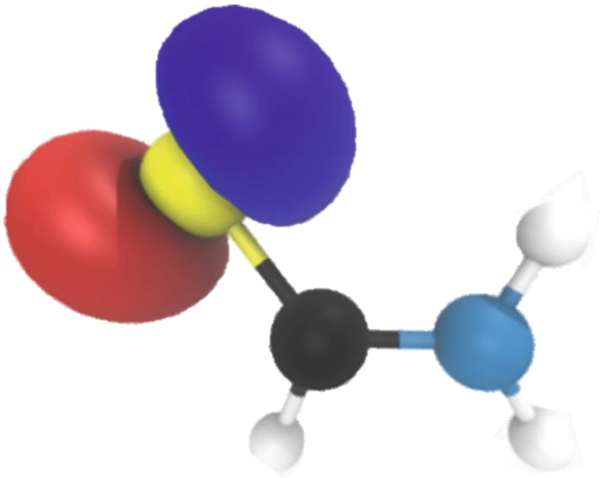

Figure S17. The HOMO NBO (A') of thioformamide (**1**) is an n-type orbital representing a free electron pair located on the sulfur in the molecular plain of symmetry.

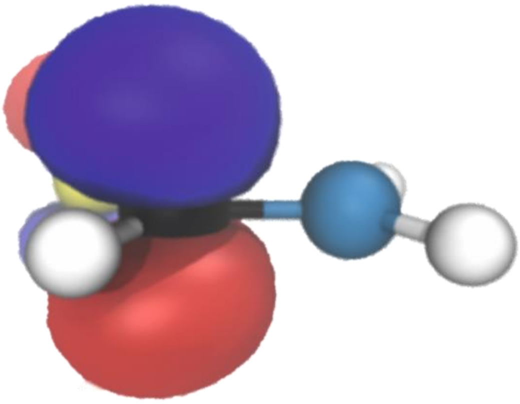

Figure S18. The LUMO NBO (A'') of thioformamide (**1**) is the antibonding π*-type orbital at the C=S bond.

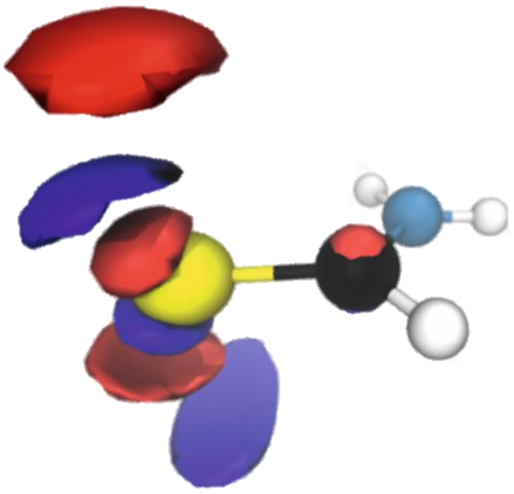

Figure S19. LUMO+1 NBO (A'') of thioformamide (**1**).

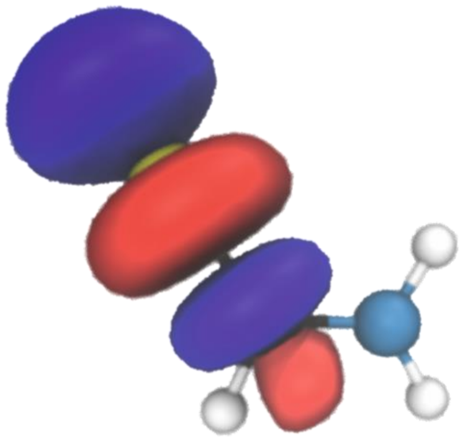

Figure S20. The LUMO+2 NBO (A') of thioformamide (**1**) is a σ*-type orbital with antibonding character along the C-S bond.

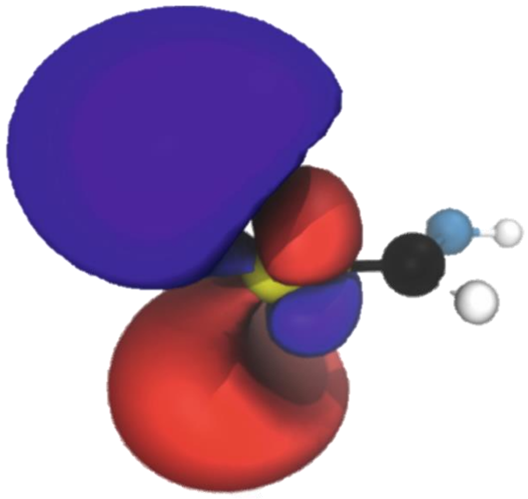

Figure S21. The LUMO+3 NBO (A'') of thioformamide (**1**) can be considered as a d-type orbital located at the sulfur.

Potential Energy Surface and Cartesian Coordinates of Selected Structures

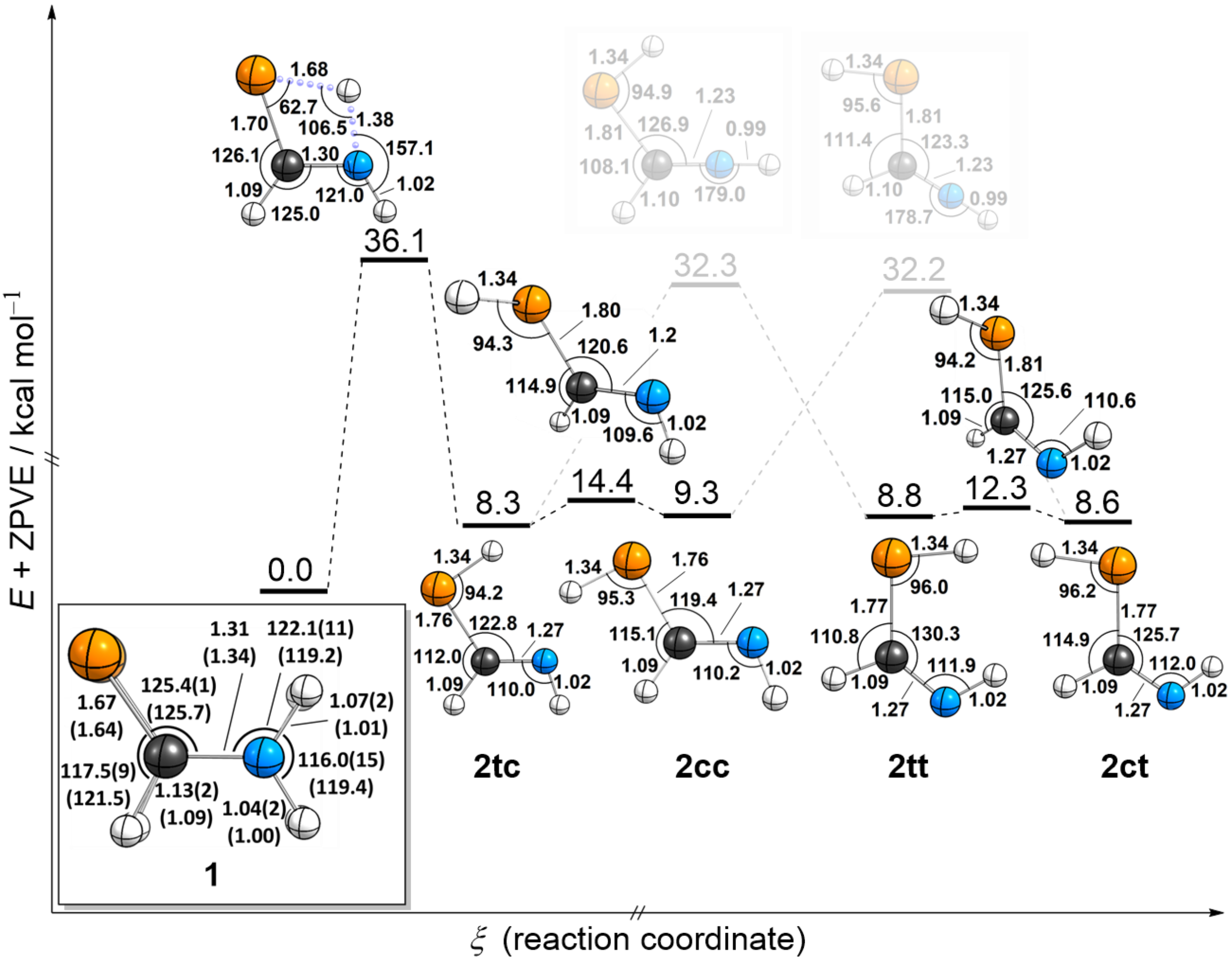

Scheme S1. Potential energy surface of thioformamide (**1**) at AE-CCSD(T)/aug-cc-pCVTZ. Bond lengths are given in Å and angles in degrees. Box: Comparison of the computed structure (background, values in brackets) and the parameters obtained from X-ray analysis (foreground, HAR data). Color code: Carbon – black, hydrogen – white, nitrogen – blue, sulfur – yellow.

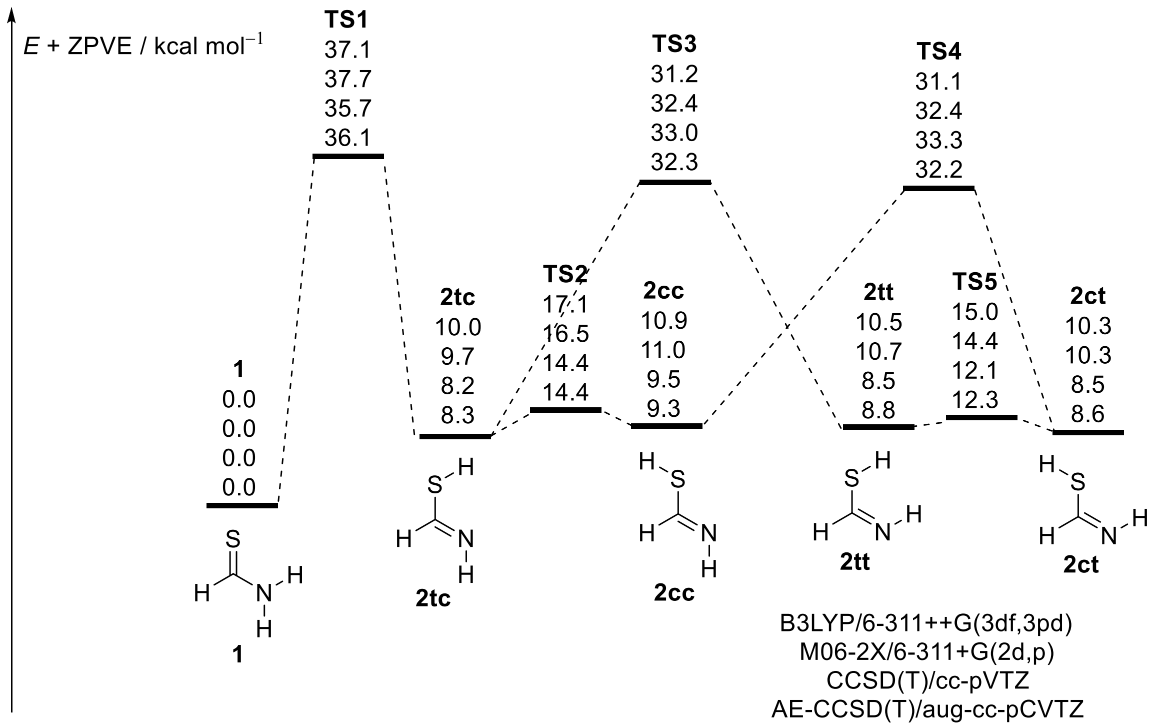

Scheme S2. Potential energy surface of thioformamide (**1**) at various levels of theory.

B3LYP/6-311++G(3df,3pd) optimized structures (Cartesian coordinates in Angstrom), electronic energies, and zero-point vibrational energies (ZPVE):

Thioformamide **1** (C_s)

6	0.000000000	0.636068000	0.000000000
7	1.327115000	0.820371000	0.000000000
1	1.941064000	0.020944000	0.000000000
1	1.730191000	1.742011000	0.000000000
16	-0.775490000	-0.806026000	0.000000000
1	-0.553218000	1.574459000	0.000000000

E = -492.9236526 au
ZPVE = 0.0436805 au

trans-cis-Thiolimine **2tc** (C_s)

6	0.000000000	0.767386000	0.000000000
7	1.228455000	1.061693000	0.000000000
1	1.367845000	2.070330000	0.000000000
1	-0.822835000	1.486625000	0.000000000
16	-0.609185000	-0.882965000	0.000000000
1	0.602756000	-1.465677000	0.000000000

E = -492.9041688 au
ZPVE = 0.0401977 au

cis-cis-Thiolimine **2cc** (C_s)

6	0.000000000	0.765229000	0.000000000
7	1.217858000	1.094158000	0.000000000
1	1.332953000	2.104994000	0.000000000
1	-0.846043000	1.455347000	0.000000000
16	-0.452135000	-0.942326000	0.000000000
1	-1.777755000	-0.733602000	0.000000000

E = -492.9026558 au
ZPVE = 0.0401015 au

cis-trans-Thiolimine **2ct** (C_s)

6	0.000000000	0.794223000	0.000000000
7	1.175422000	1.247267000	0.000000000
1	1.893746000	0.523126000	0.000000000
1	-0.840865000	1.484225000	0.000000000
16	-0.467860000	-0.923699000	0.000000000
1	-1.795070000	-0.724372000	0.000000000

E = -492.9036576 au
ZPVE = 0.0400656 au

trans-trans-Thiolimine **2tt** (C_s)

6	0.000000000	0.793915000	0.000000000
7	1.178410000	1.240820000	0.000000000
1	1.891342000	0.512377000	0.000000000
1	-0.833292000	1.495705000	0.000000000
16	-0.617710000	-0.872945000	0.000000000
1	0.576449000	-1.490189000	0.000000000

E = -492.9034068 au
ZPVE = 0.0402410 au

TS1 (C_s)

7	-1.407111000	-0.374088000	0.000000000
6	-0.538093000	0.585723000	0.000000000
16	1.031761000	-0.075096000	0.000000000
1	-2.406399000	-0.182257000	0.000001000
1	-0.776841000	1.644841000	0.000001000
1	-0.246596000	-1.156772000	0.000000000

E = -492.858073 au
ZPVE = 0.0372829 au
v_i = 1718.3*i* cm^{-1}

TS2 (C_1)

6	-0.660425000	0.438404000	0.022449000
7	-1.587484000	-0.408893000	-0.017782000
1	-2.501241000	0.045603000	0.032891000
1	-0.807123000	1.518322000	0.082746000
16	1.073019000	-0.066674000	-0.085102000
1	1.214995000	-0.265313000	1.235782000

E = -492.8919372 au
ZPVE = 0.0392443 au
v_i = 355.3*i* cm^{-1}

TS3 (C_s)

6	0.000000000	0.804508000	0.000000000
7	1.162157000	1.173278000	0.000000000
1	2.098587000	1.489305000	0.000000000
1	-0.877741000	1.468426000	0.000000000
16	-0.621367000	-0.906423000	0.000000000
1	0.585935000	-1.494953000	0.000000000

E = -492.8675483 au
ZPVE = 0.0372559 au
v_i = 1065.0*i* cm^{-1}

TS4 (C_s)

6	0.000000000	0.799597000	0.000000000
7	1.154205000	1.191500000	0.000000000
1	2.082113000	1.531697000	0.000000000
1	-0.893897000	1.440368000	0.000000000
16	-0.467036000	-0.959631000	0.000000000
1	-1.795078000	-0.756059000	0.000000000

E = -492.86765 au
ZPVE = 0.0371921 au
v_i = 1056.7*i* cm^{-1}

TS5 (C_1)
6 -0.676535000 0.483907000 0.026278000
7 -1.690775000 -0.252790000 -0.004803000
1 -1.448881000 -1.240881000 -0.087615000
1 -0.795768000 1.562634000 0.092028000
16 1.060656000 -0.068994000 -0.084544000
1 1.168781000 -0.351767000 1.224247000
E = -492.8954888 au
ZPVE = 0.0394490 au
v_i = 290.8*i* cm^{-1}

Methylensulfinilimine **32** (C_s)
6 1.302508000 -0.503042000 0.000000000
16 0.000000000 0.442182000 0.000000000
1 2.250300000 0.010675000 0.000000000
1 1.244084000 -1.581322000 0.000000000
7 -1.317989000 -0.394594000 0.000000000
1 -2.083515000 0.276150000 0.000000000
E = -492.8536349 au
ZPVE = 0.0404599 au

Thiaziridine **33** (C_1)
7 0.743281000 0.786439000 -0.157149000
16 -0.848733000 -0.045429000 0.009275000
6 0.847806000 -0.637510000 0.020032000
1 1.216117000 -1.024124000 0.962792000
1 1.157854000 -1.179769000 -0.863506000
1 0.915956000 1.250746000 0.732173000
E = -492.8673739 au
ZPVE = 0.0434991 au

M06-2X/6-311+G(2d,p) optimized structures (Cartesian coordinates in Angstrom), electronic energies, and zero-point vibrational energies (ZPVE):

Thioformamide **1** (C_s)
7 1.325291000 0.813992000 0.000000000
6 0.000000000 0.636691000 0.000000000
16 -0.774305000 -0.802073000 0.000000000
1 -0.553212000 1.575489000 0.000000000
1 1.930141000 0.006246000 0.000000000
1 1.734921000 1.733335000 0.000000000
E = -492.8308471au
ZPVE = 0.0440136 au

trans-cis-Thiolimine **2tc** (C_s)
7 1.228694000 1.048863000 0.000000000
6 0.000000000 0.771512000 0.000000000
16 -0.610321000 -0.880546000 0.000000000
1 1.371633000 2.058306000 0.000000000
1 -0.814136000 1.500046000 0.000000000
1 0.606777000 -1.440730000 0.000000000
E = -492.8118064 au
ZPVE = 0.0405039 au

cis-cis-Thiolimine **2cc** (C_s)
7 1.216126000 1.088501000 0.000000000
6 0.000000000 0.766921000 0.000000000
16 -0.451577000 -0.941150000 0.000000000
1 1.325335000 2.101489000 0.000000000
1 -0.842194000 1.461805000 0.000000000
1 -1.770796000 -0.725928000 0.000000000
E = -492.8096584 au
ZPVE = 0.0402975 au

cis-trans-Thiolimine **2ct** (C_s)

7	1.695105000	-0.197699000	0.000001000
6	0.637150000	0.479711000	-0.000001000
16	-1.015989000	-0.178652000	0.000000000
1	-1.643980000	1.001270000	-0.000001000
1	1.519626000	-1.203977000	-0.000003000
1	0.691547000	1.566765000	0.000001000

E = -492.8105886 au
ZPVE = 0.0405231 au

trans-trans-Thiolimine **2tt** (C_s)

7	1.182112000	1.225081000	0.000000000
6	0.000000000	0.797869000	0.000000000
16	-0.618815000	-0.867389000	0.000000000
1	1.873751000	0.474526000	0.000000000
1	-0.825391000	1.509458000	0.000000000
1	0.577903000	-1.468532000	0.000000000

E = -492.8105197 au
ZPVE = 0.0407076 au

TS1 (C_s)

7	1.403568000	-0.375871000	0.000000000
6	0.542242000	0.583608000	0.000000000
16	-1.032045000	-0.074179000	0.000000000
1	2.403259000	-0.185137000	-0.000001000
1	0.787039000	1.642333000	-0.000001000
1	0.243994000	-1.140876000	0.000000000

E = -492.7645516 au
ZPVE = 0.0378021 au
v_i = 1736.7*i* cm^{-1}

TS2 (C_1)

7	-1.580056000	-0.411249000	-0.016794000
6	-0.659346000	0.439688000	0.018717000
16	1.070189000	-0.067405000	-0.084020000
1	1.196113000	-0.249881000	1.236048000
1	-2.491848000	0.049916000	0.035351000
1	-0.810815000	1.519071000	0.078181000

E = -492.8003824 au
ZPVE = 0.0398479 au
v_i = 409.3*i* cm^{-1}

TS3 (C_s)

7	1.624737000	-0.239394000	0.000000000
6	0.645183000	0.483117000	0.000000000
16	-1.094493000	-0.039321000	0.000000000
1	2.422094000	-0.827518000	0.000001000
1	0.668387000	1.582926000	0.000000000
1	-0.822849000	-1.349220000	-0.000001000

E = -492.7731861 au
ZPVE = 0.0379152 au
v_i = 1079.6*i* cm^{-1}

TS4 (C_s)

7	1.638414000	-0.219054000	0.000000000
6	0.641236000	0.477611000	0.000000000
16	-1.045821000	-0.194674000	0.000000000
1	2.450614000	-0.786487000	0.000000000
1	0.631371000	1.577325000	0.000000000
1	-1.665161000	0.991656000	-0.000001000

E = -492.7728717 au
ZPVE = 0.0377497 au
v_i = 1067.3*i* cm^{-1}

TS5 (C_1)

7	-1.680384000	-0.257364000	-0.007347000
6	-0.674425000	0.487041000	0.028263000
16	1.055628000	-0.065605000	-0.083271000
1	1.132611000	-0.396397000	1.211627000
1	-1.413615000	-1.239481000	-0.104498000
1	-0.799807000	1.564854000	0.107056000

E = -492.8038586 au
ZPVE = 0.0399336 au
v_i = 377.8i cm^{-1}

CCSD(T)/cc-pVTZ optimized structures (Cartesian coordinates in Angstrom), electronic energies, and zero-point vibrational energies (ZPVE):

Thioformamide **1** (C_s)

7	-1.616849701	0.247351067	0.000000000
6	-0.493530007	-0.492573037	0.000000000
1	-1.542299861	1.251029882	0.000000000
1	-2.525174220	-0.180601951	0.000000000
16	1.043531524	0.092069322	0.000000000
1	-0.695889932	-1.563019087	0.000000000

E = −492.2361325 au
ZPVE = 27.3894 kcal mol^{-1}

trans-cis-Thiolimine **2tc** (C_s)

7	1.626672956	0.341454673	0.000000000
6	0.651377511	-0.479584244	0.000000000
1	2.505080540	-0.177093622	0.000000000
16	-1.036183802	0.042876195	0.000000000
1	0.739079802	-1.567954866	0.000000000
1	-0.729844971	1.350887931	0.000000000

E = -492.2197601 au
ZPVE = 25.3080 kcal mol^{-1}

cis-cis-Thiolimine **2cc** (C_s)

7	1.624063197	0.338617077	0.000000000
6	0.654663749	-0.486921006	0.000000000
1	2.506126231	-0.172154673	0.000000000
16	-1.009561752	0.123805738	0.000000000
1	0.744237444	-1.574531069	0.000000000
1	-1.583469539	-1.088085708	0.000000000

E = -492.2175532 au
ZPVE = 25.1989 kcal mol^{-1}

cis-trans-Thiolimine **2ct** (C_s)

7	-1.711935714	0.230263637	0.000000000
6	-0.659804653	-0.484020009	0.000000000
1	-1.484194670	1.226760903	0.000000000
16	1.017235388	0.125560040	0.000000000
1	-0.750778705	-1.567434923	0.000000000
1	1.606785533	-1.078791747	0.000000000

E = -492.2190790 au
ZPVE = 25.1656 kcal mol^{-1}

trans-trans-Thiolimine **2tt** (C_s)

7	-1.724873373	0.227644422	0.000000000
6	-0.658930087	-0.466936500	0.000000000
1	-1.516160691	1.227710072	0.000000000
16	1.048695526	0.042852938	0.000000000
1	-0.736635310	-1.552666391	0.000000000
1	0.795919233	1.362258510	0.000000000

E = -492.2192712 au
ZPVE = 25.3012 kcal mol^{-1}

TS1 (C_s)

7	1.468584486	0.360315736	0.000000000
6	0.591190128	-0.602872309	0.000000000
1	2.465285009	0.156698062	0.000000000
16	-0.979282733	0.080176439	0.000000000
1	0.828230172	-1.662662983	0.000000000
1	0.328857593	1.134405349	0.000000000

E = -492.1730360 au
ZPVE = 23.5345 kcal mol^{-1}
v_i = 1775.9i cm^{-1}

TS2 (C_1)

7	1.632526715	0.358770305	-0.008149097
6	0.679389916	-0.482733447	0.015087669
1	2.521709829	-0.147090013	0.015225022
16	-1.039023441	0.070940211	-0.044285563
1	0.809069108	-1.565641870	0.038376802
1	-1.141252517	0.225180879	1.284886034

E = -492.2089095 au
ZPVE = 24.7133 kcal mol^{-1}
v_i = 316.5i cm^{-1}

TS3 (C_s)

6	-0.682833193	-0.478126969	0.000000000
1	-0.700424881	-1.578015722	0.000000000
16	1.063814057	0.051664840	0.000000000
7	-1.670923260	0.248352789	0.000000000
1	0.776369981	1.362592585	0.000000000
1	-2.477431161	0.818691826	0.000000000

E = -492.1772956 au
ZPVE = 23.4965 kcal mol^{-1}
v_i = 1124.4i cm^{-1}

TS4 (C_s)

6	-0.681553113	0.487376739	0.000000000
16	1.033753032	-0.133433382	0.000000000
1	-0.702127879	1.587319880	0.000000000
7	-1.663737462	-0.246155068	0.000000000
1	1.608883795	1.079837369	0.000000000
1	-2.469674836	-0.817085858	0.000000000

E = -492.1767569 au
ZPVE = 23.4189 kcal mol^{-1}
v_i = 1116.2i cm^{-1}

TS5 (C_1)

7	-1.711128920	0.260811495	0.008561157
6	-0.688202580	-0.489770176	-0.022009487
1	-1.423735164	1.240273793	0.067273679
16	1.043561925	0.068935698	0.044369994
1	-0.820006270	-1.567648055	-0.064703476
1	1.107294323	0.348273175	-1.267044812

E = -492.2127693 au
ZPVE = 24.8313 kcal mol^{-1}
v_i = 257.4i cm^{-1}

AE-CCSD(T)/aug-cc-pCVTZ optimized structures (Cartesian coordinates in Angstrom), electronic energies, and zero-point vibrational energies (ZPVE).

Thioformamide **1** (C_s)

7	-1.612358981	0.246697607	0.000000000
6	-0.490242927	-0.492256637	0.000000000
1	-1.539785327	1.250839732	0.000000000
1	-2.520710277	-0.181551819	0.000000000
16	1.039914200	0.092266128	0.000000000
1	-0.689647419	-1.562810380	0.000000000

E = −492.6898924 au
ZPVE = 27.3710 kcal mol^{-1}

trans-*cis*-Thiolimine **2tc** (C_s)

7	1.622608044	0.339394846	0.000000000
6	0.645932779	-0.477900249	0.000000000
1	2.504389604	-0.171990750	0.000000000
16	-1.032211660	0.042976643	0.000000000
1	0.735916947	-1.565870151	0.000000000
1	-0.730694031	1.349082621	0.000000000

E = -492.6733651 au
ZPVE = 25.2861 kcal mol^{-1}

cis-*cis*-Thiolimine **2cc** (C_s)

7	1.618763471	0.336752669	0.000000000
6	0.648965690	-0.486275812	0.000000000
1	2.505212273	-0.164959863	0.000000000
16	-1.004981305	0.123939000	0.000000000
1	0.740756791	-1.573315564	0.000000000
1	-1.582902256	-1.082501173	0.000000000

E = -492.6716301 au
ZPVE = 25.2023 kcal mol^{-1}

cis-*trans*-Thiolimine **2ct** (C_s)

7	-1.705426834	0.229103512	0.000000000
6	-0.654295135	-0.484011955	0.000000000
1	-1.490267984	1.227798027	0.000000000
16	1.012414340	0.125864317	0.000000000
1	-0.745383378	-1.567102854	0.000000000
1	1.604368072	-1.073790524	0.000000000

E = -492.6726852 au
ZPVE = 25.1651 kcal mol^{-1}

trans-*trans*-Thiolimine **2tt** (C_s)

7	-1.719000109	0.226339656	0.000000000
6	-0.653623847	-0.466249046	0.000000000
1	-1.522735840	1.228358653	0.000000000
16	1.044212792	0.043188844	0.000000000
1	-0.731863523	-1.551803176	0.000000000
1	0.795146280	1.360034082	0.000000000

E = -492.6726125 au
ZPVE = 25.2806 kcal mol^{-1}

TS1 (C_s)

7	1.465470813	0.358445782	0.000000000
6	0.586408832	-0.601652307	0.000000000
1	2.462154613	0.156531366	0.000000000
16	-0.975759566	0.080395111	0.000000000
1	0.822176336	-1.661350904	0.000000000
1	0.326465939	1.137778378	0.000000000

E = -492.6261502 au
ZPVE = 23.4572 kcal mol^{-1}
v_i = 1786.4i cm^{-1}

TS2 (C_1)

7	1.627239001	0.357264479	-0.008337161
6	0.674970725	-0.482149521	0.015437455
1	2.521229183	-0.138453835	0.015278570
16	-1.035145355	0.071019863	-0.044214844
1	0.807705127	-1.564456708	0.037685342
1	-1.136347694	0.226802485	1.281728671

E = -492.6626567 au
ZPVE = 24.7119 kcal mol^{-1}
v_i = 321.8i cm^{-1}

TS3 (C_s)

6	-0.674440944	-0.476644892	0.000000000
1	-0.695978622	-1.575771481	0.000000000
16	1.058061360	0.051936763	0.000000000
7	-1.665588513	0.246596847	0.000000000
1	0.777098007	1.361253439	0.000000000
1	-2.474155834	0.815910978	0.000000000

E = -492.6322098 au
ZPVE = 23.4313 kcal mol^{-1}
v_i = 1123.1i cm^{-1}

TS4 (C_s)

6	-0.672997359	0.487394678	0.000000000
16	1.027352532	-0.133820830	0.000000000
1	-0.697998016	1.586174391	0.000000000
7	-1.657059404	-0.244787452	0.000000000
1	1.607012852	1.073890565	0.000000000
1	-2.463544571	-0.816917981	0.000000000

E = -492.6321514 au
ZPVE = 23.3848 kcal mol^{-1}
v_i = 1114.3i cm^{-1}

TS5 (C_1)

7	-1.704803326	0.260047035	0.007906120
6	-0.683921525	-0.489758947	-0.021067541
1	-1.427984875	1.242118330	0.064798918
16	1.039264386	0.069609817	0.044243113
1	-0.816285847	-1.567500721	-0.060731498
1	1.105294240	0.335383592	-1.266631223

E = -492.6661468 au
ZPVE = 24.8161 kcal mol^{-1}
v_i = 259.2i cm^{-1}

Tunneling Computations

Table S28. Rate constants in s^{-1} of the reaction **2tc** → **1** *via* **TS1** obtained with Polyrate at the CVT/SCT//B3LYP/6-311++G(3df,3pd) level of theory.

Temperature / K	TST	CVT	CVT/ZCT	CVT/SCT
0.10	NaN	NaN	NaN	NaN
8.00	0.00	0.00	NaN	NaN
10.00	0.00	0.00	NaN	NaN
15.00	0.00	0.00	NaN	NaN
20.00	$3.09 \cdot 10^{-285}$	$3.04 \cdot 10^{-285}$	$2.53 \cdot 10^{-10}$	$8.37 \cdot 10^{-8}$
25.00	$6.44 \cdot 10^{-226}$	$6.36 \cdot 10^{-226}$	$2.53 \cdot 10^{-10}$	$8.37 \cdot 10^{-8}$
30.00	$2.34 \cdot 10^{-186}$	$2.32 \cdot 10^{-186}$	$2.53 \cdot 10^{-10}$	$8.37 \cdot 10^{-8}$
35.00	$4.34 \cdot 10^{-158}$	$4.30 \cdot 10^{-158}$	$2.53 \cdot 10^{-10}$	$8.37 \cdot 10^{-8}$
40.00	$7.01 \cdot 10^{-137}$	$6.96 \cdot 10^{-137}$	$2.53 \cdot 10^{-10}$	$8.37 \cdot 10^{-8}$
45.00	$2.23 \cdot 10^{-120}$	$2.21 \cdot 10^{-120}$	$2.53 \cdot 10^{-10}$	$8.37 \cdot 10^{-8}$
50.00	$3.58 \cdot 10^{-107}$	$3.56 \cdot 10^{-107}$	$2.53 \cdot 10^{-10}$	$8.37 \cdot 10^{-8}$
200.00	$8.86 \cdot 10^{-18}$	$8.85 \cdot 10^{-18}$	$6.19 \cdot 10^{-9}$	$2.41 \cdot 10^{-7}$
250.00	$8.61 \cdot 10^{-12}$	$8.60 \cdot 10^{-12}$	$3.89 \cdot 10^{-7}$	$2.69 \cdot 10^{-6}$
300.00	$8.49 \cdot 10^{-8}$	$8.49 \cdot 10^{-8}$	$2.76 \cdot 10^{-5}$	$7.15 \cdot 10^{-5}$
350.00	$6.07 \cdot 10^{-5}$	$6.07 \cdot 10^{-5}$	$1.77 \cdot 10^{-3}$	$2.58 \cdot 10^{-3}$

Table S29. Rate constants in s^{-1} of the reaction **2cc** → **2tc** *via* **TS2** obtained with Polyrate at the CVT/SCT//B3LYP/6-311++G(3df,3pd) level of theory.

Temperature / K	TST	CVT	CVT/ZCT	CVT/SCT
0.10	NaN	NaN	NaN	NaN
8.00	$1.60 \cdot 10^{-182}$	$1.64 \cdot 10^{-182}$	$3.08 \cdot 10^{-9}$	$6.52 \cdot 10^{-7}$
10.00	$9.31 \cdot 10^{-144}$	$9.50 \cdot 10^{-144}$	$3.13 \cdot 10^{-9}$	$6.62 \cdot 10^{-7}$
15.00	$5.03 \cdot 10^{-92}$	$5.09 \cdot 10^{-92}$	$3.19 \cdot 10^{-9}$	$6.75 \cdot 10^{-7}$
20.00	$4.02 \cdot 10^{-66}$	$4.06 \cdot 10^{-66}$	$3.22 \cdot 10^{-9}$	$6.81 \cdot 10^{-7}$
25.00	$1.47 \cdot 10^{-50}$	$1.48 \cdot 10^{-50}$	$3.24 \cdot 10^{-9}$	$6.85 \cdot 10^{-7}$
30.00	$3.62 \cdot 10^{-40}$	$3.64 \cdot 10^{-40}$	$3.26 \cdot 10^{-9}$	$6.88 \cdot 10^{-7}$
35.00	$9.79 \cdot 10^{-33}$	$9.84 \cdot 10^{-33}$	$3.27 \cdot 10^{-9}$	$6.90 \cdot 10^{-7}$
40.00	$3.74 \cdot 10^{-27}$	$3.76 \cdot 10^{-27}$	$3.27 \cdot 10^{-9}$	$6.92 \cdot 10^{-7}$
45.00	$8.32 \cdot 10^{-23}$	$8.36 \cdot 10^{-23}$	$3.28 \cdot 10^{-9}$	$6.93 \cdot 10^{-7}$
50.00	$2.53 \cdot 10^{-19}$	$2.54 \cdot 10^{-19}$	$3.28 \cdot 10^{-9}$	$6.94 \cdot 10^{-7}$
200.00	$1.50 \cdot 10^{5}$	$1.51 \cdot 10^{5}$	$1.05 \cdot 10^{5}$	$1.24 \cdot 10^{5}$
250.00	$6.30 \cdot 10^{6}$	$6.31 \cdot 10^{6}$	$4.42 \cdot 10^{6}$	$4.63 \cdot 10^{6}$
300.00	$7.69 \cdot 10^{7}$	$7.70 \cdot 10^{7}$	$5.58 \cdot 10^{7}$	$5.61 \cdot 10^{7}$
350.00	$4.63 \cdot 10^{8}$	$4.63 \cdot 10^{8}$	$3.47 \cdot 10^{8}$	$3.43 \cdot 10^{8}$

Table S30. Rate constants in s^{-1} of the reaction **2tt** → **2ct** *via* **TS5** obtained with Polyrate at the CVT/SCT//B3LYP/6-311++G(3df,3pd) level of theory.

Temperature / K	TST	CVT	CVT/ZCT	CVT/SCT
0.10	NaN	NaN	NaN	NaN
8.00	$2.42 \cdot 10^{-111}$	$2.43 \cdot 10^{-111}$	$5.67 \cdot 10^{-4}$	$4.21 \cdot 10^{-2}$
10.00	$8.18 \cdot 10^{-87}$	$8.19 \cdot 10^{-87}$	$5.67 \cdot 10^{-4}$	$4.21 \cdot 10^{-2}$
15.00	$4.61 \cdot 10^{-54}$	$4.61 \cdot 10^{-54}$	$5.66 \cdot 10^{-4}$	$4.20 \cdot 10^{-2}$
20.00	$1.19 \cdot 10^{-37}$	$1.19 \cdot 10^{-37}$	$5.66 \cdot 10^{-4}$	$4.20 \cdot 10^{-2}$
25.00	$8.82 \cdot 10^{-28}$	$8.83 \cdot 10^{-28}$	$5.66 \cdot 10^{-4}$	$4.20 \cdot 10^{-2}$
30.00	$3.47 \cdot 10^{-21}$	$3.47 \cdot 10^{-21}$	$5.68 \cdot 10^{-4}$	$4.20 \cdot 10^{-2}$
35.00	$1.82 \cdot 10^{-16}$	$1.82 \cdot 10^{-16}$	$5.80 \cdot 10^{-4}$	$4.24 \cdot 10^{-2}$
40.00	$6.43 \cdot 10^{-13}$	$6.44 \cdot 10^{-13}$	$6.51 \cdot 10^{-4}$	$4.42 \cdot 10^{-2}$
45.00	$3.75 \cdot 10^{-10}$	$3.75 \cdot 10^{-10}$	$1.02 \cdot 10^{-3}$	$5.12 \cdot 10^{-2}$
50.00	$6.19 \cdot 10^{-8}$	$6.19 \cdot 10^{-8}$	$3.23 \cdot 10^{-3}$	$7.91 \cdot 10^{-2}$
200.00	$1.02 \cdot 10^{8}$	$1.02 \cdot 10^{8}$	$1.79 \cdot 10^{8}$	$2.28 \cdot 10^{8}$
250.00	$1.14 \cdot 10^{9}$	$1.14 \cdot 10^{9}$	$1.64 \cdot 10^{9}$	$1.92 \cdot 10^{9}$
300.00	$5.77 \cdot 10^{9}$	$5.77 \cdot 10^{9}$	$7.40 \cdot 10^{9}$	$8.31 \cdot 10^{9}$
350.00	$1.84 \cdot 10^{10}$	$1.84 \cdot 10^{10}$	$2.22 \cdot 10^{10}$	$2.42 \cdot 10^{10}$

Table S31. Rate constants in s^{-1} of the reaction **2tt** → **2tc** *via* **TS3** obtained with Polyrate at the CVT/SCT//B3LYP/6-311++G(3df,3pd) level of theory.

Temperature / K	TST	CVT	CVT/ZCT	CVT/SCT
0.10	NaN	NaN	NaN	NaN
8.00	0.00	0.00	NaN	NaN
10.00	0.00	0.00	NaN	NaN
15.00	$8.84 \cdot 10^{-290}$	$8.65 \cdot 10^{-290}$	$7.47 \cdot 10^{-10}$	$2.37 \cdot 10^{-8}$
20.00	$1.62 \cdot 10^{-214}$	$1.59 \cdot 10^{-214}$	$7.45 \cdot 10^{-10}$	$2.36 \cdot 10^{-8}$
25.00	$2.44 \cdot 10^{-169}$	$2.41 \cdot 10^{-169}$	$7.44 \cdot 10^{-10}$	$2.36 \cdot 10^{-8}$
30.00	$3.33 \cdot 10^{-139}$	$3.29 \cdot 10^{-139}$	$7.44 \cdot 10^{-10}$	$2.36 \cdot 10^{-8}$
35.00	$1.14 \cdot 10^{-117}$	$1.13 \cdot 10^{-117}$	$7.43 \cdot 10^{-10}$	$2.36 \cdot 10^{-8}$
40.00	$1.64 \cdot 10^{-101}$	$1.63 \cdot 10^{-101}$	$7.43 \cdot 10^{-10}$	$2.36 \cdot 10^{-8}$
45.00	$6.16 \cdot 10^{-89}$	$6.11 \cdot 10^{-89}$	$7.43 \cdot 10^{-10}$	$2.36 \cdot 10^{-8}$
50.00	$7.14 \cdot 10^{-79}$	$7.09 \cdot 10^{-79}$	$7.43 \cdot 10^{-10}$	$2.38 \cdot 10^{-8}$
200.00	$1.22 \cdot 10^{-10}$	$1.22 \cdot 10^{-10}$	$6.07 \cdot 10^{-8}$	$7.65 \cdot 10^{-6}$
250.00	$5.07 \cdot 10^{-6}$	$5.06 \cdot 10^{-6}$	$5.62 \cdot 10^{-5}$	$4.20 \cdot 10^{-4}$
300.00	$6.37 \cdot 10^{-3}$	$6.41 \cdot 10^{-3}$	$2.30 \cdot 10^{-2}$	$6.45 \cdot 10^{-2}$
350.00	1.08	1.08	2.31	4.66

Table S32. Rate constants in s^{-1} of the reaction **2cc** → **2ct** *via* **TS4** obtained with Polyrate at the CVT/SCT//B3LYP/6-311++G(3df,3pd) level of theory.

Temperature / K	TST	CVT	CVT/ZCT	CVT/SCT
0.10	NaN	NaN	NaN	NaN
8.00	0.00	0.00	NaN	NaN
10.00	0.00	0.00	NaN	NaN
15.00	$1.30 \cdot 10^{-295}$	$1.27 \cdot 10^{-295}$	$1.31 \cdot 10^{-11}$	$2.06 \cdot 10^{-8}$
20.00	$6.91 \cdot 10^{-219}$	$6.79 \cdot 10^{-219}$	$1.32 \cdot 10^{-11}$	$2.08 \cdot 10^{-8}$
25.00	$7.86 \cdot 10^{-173}$	$7.76 \cdot 10^{-173}$	$1.32 \cdot 10^{-11}$	$2.08 \cdot 10^{-8}$
30.00	$4.12 \cdot 10^{-142}$	$4.07 \cdot 10^{-142}$	$1.33 \cdot 10^{-11}$	$2.09 \cdot 10^{-8}$
35.00	$3.69 \cdot 10^{-120}$	$3.65 \cdot 10^{-120}$	$1.33 \cdot 10^{-11}$	$2.09 \cdot 10^{-8}$
40.00	$1.09 \cdot 10^{-103}$	$1.09 \cdot 10^{-103}$	$1.33 \cdot 10^{-11}$	$2.10 \cdot 10^{-8}$
45.00	$7.19 \cdot 10^{-91}$	$7.14 \cdot 10^{-91}$	$1.33 \cdot 10^{-11}$	$2.10 \cdot 10^{-8}$
50.00	$1.31 \cdot 10^{-80}$	$1.30 \cdot 10^{-80}$	$1.34 \cdot 10^{-11}$	$2.10 \cdot 10^{-8}$
200.00	$4.98 \cdot 10^{-11}$	$4.97 \cdot 10^{-11}$	$1.95 \cdot 10^{-8}$	$6.00 \cdot 10^{-7}$
250.00	$2.60 \cdot 10^{-6}$	$2.59 \cdot 10^{-6}$	$2.84 \cdot 10^{-5}$	$1.44 \cdot 10^{-4}$
300.00	$3.81 \cdot 10^{-3}$	$3.81 \cdot 10^{-3}$	$1.44 \cdot 10^{-2}$	$3.57 \cdot 10^{-2}$
350.00	$7.22 \cdot 10^{-1}$	$7.21 \cdot 10^{-1}$	1.66	3.10

Table S33. Computed Tunneling half-lives obtained with Tunnex at the M06-2X/6-311+G(2d,p) level of theory.

Reaction	Symmetry Number[a]	Tunneling half-life
**2tt → 2ct**	2	3.5 h
**2cc → 2tc**	2	4.1 d
**2cc → 2ct**	1	127 a
**2tt → 2tc**	1	177 a

[a] accounts for the degeneracy of the reaction coordinate.

Kinetic Analysis

Table S34. Experimental tunneling half-lives of **2tt** reacting to **2ct** under different conditions. If not specified otherwise, a 4.5 µm cut-off filter was used during IR measurements. IR bands are given in cm^{-1} and half-lives in min. In some experiments some IR intensities were too low to follow their kinetics reliably. These entries are marked with a hyphen.

IR Band (Ar)	Ar (3 K, without filter)	Ar (3 K)	Ar (20 K)	N_2 (3 K, without filter)	N_2 (3 K)	N_2 (20 K)
1176.9 (**2tt**)	24	36	25	25	45	25
1358.0 (**2tt**)	25	35	25	-	-	-
1173.1 (**2ct**)	24	35	27	26	-	26
1346.2 (**2ct**)	27	36	25	25	-	26

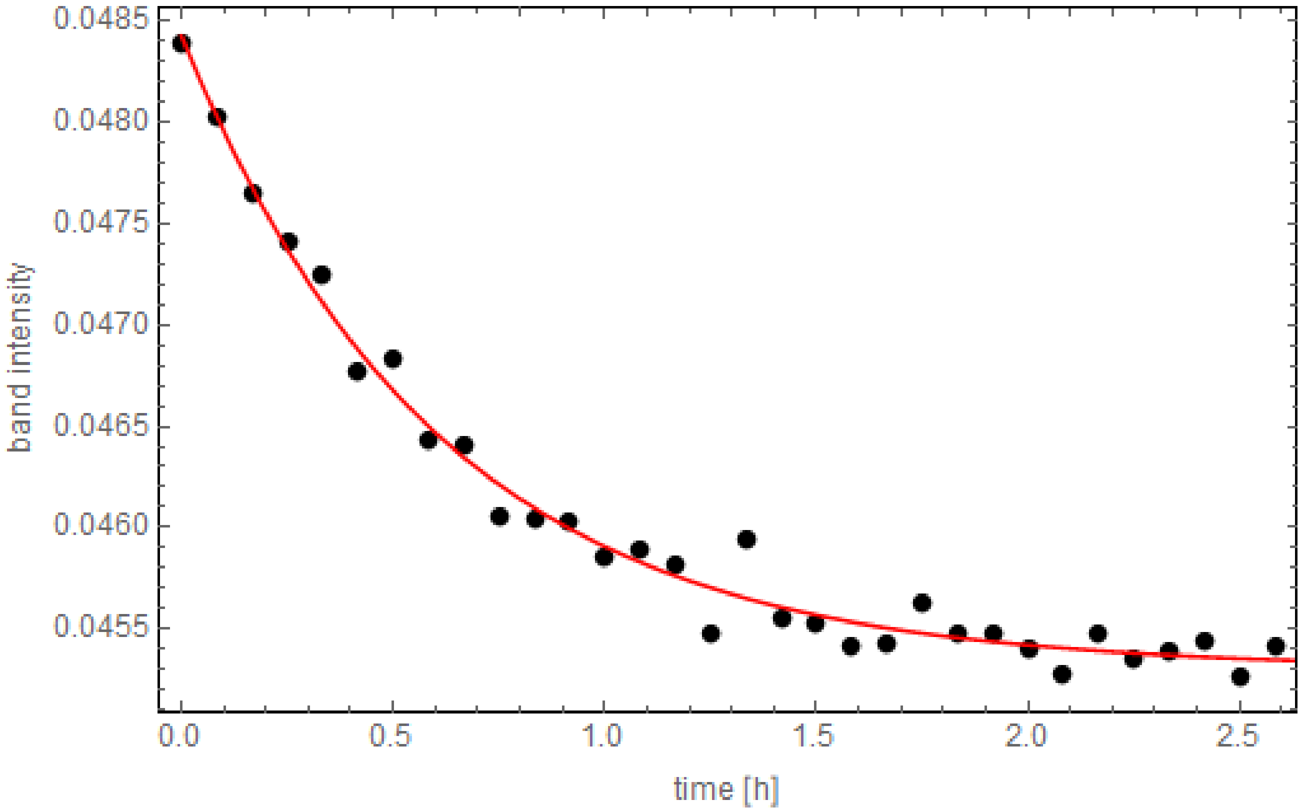

Figure S22. Decay of the IR band at 1358.0 cm^{-1} (**2tt**) in an Ar matrix at 3 K followed over time. A 4.5 µm cut-off filter was used.

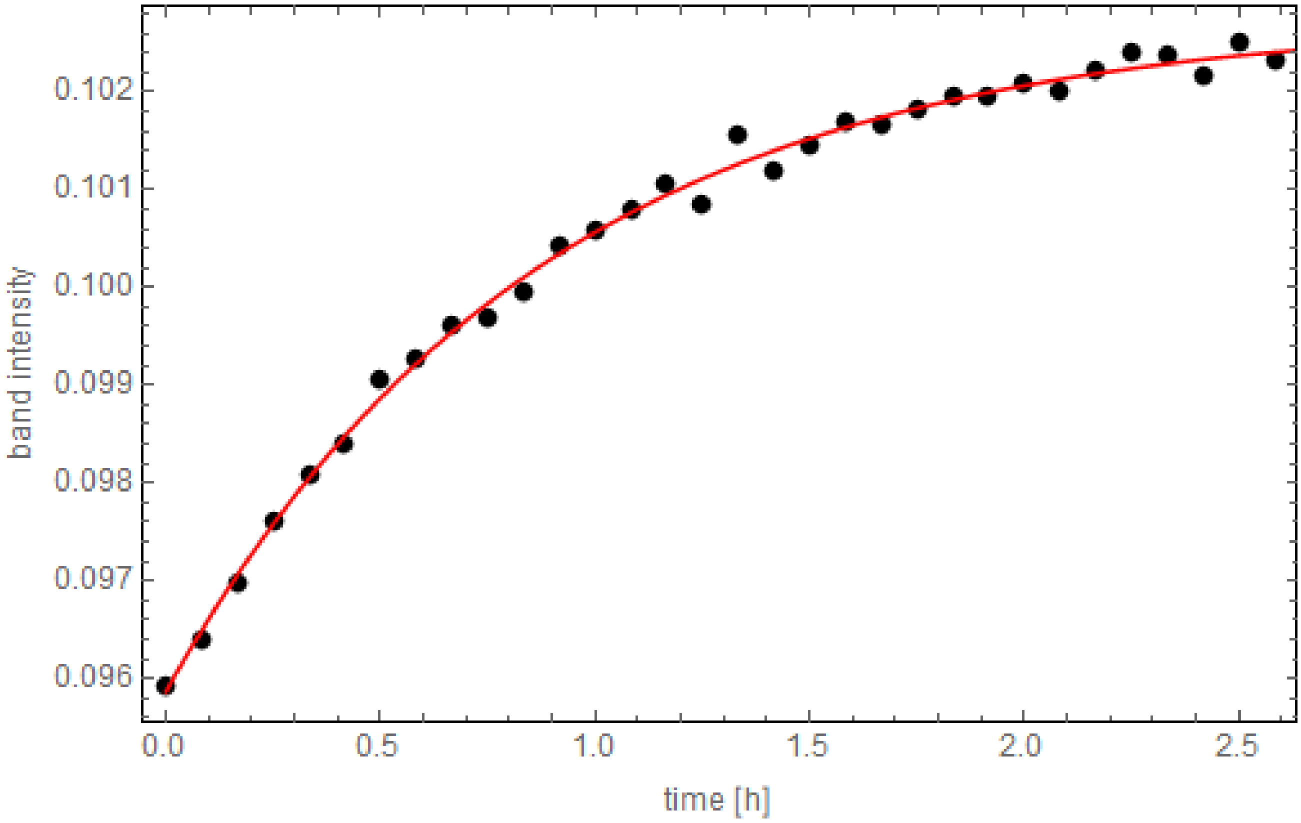

Figure S23. Increase of the IR band at 1346.2 cm^{-1} (**2ct**) in an Ar matrix at 3 K followed over time. A 4.5 μm cut-off filter was used.

Other Matrix Isolation Studies of Thioamides

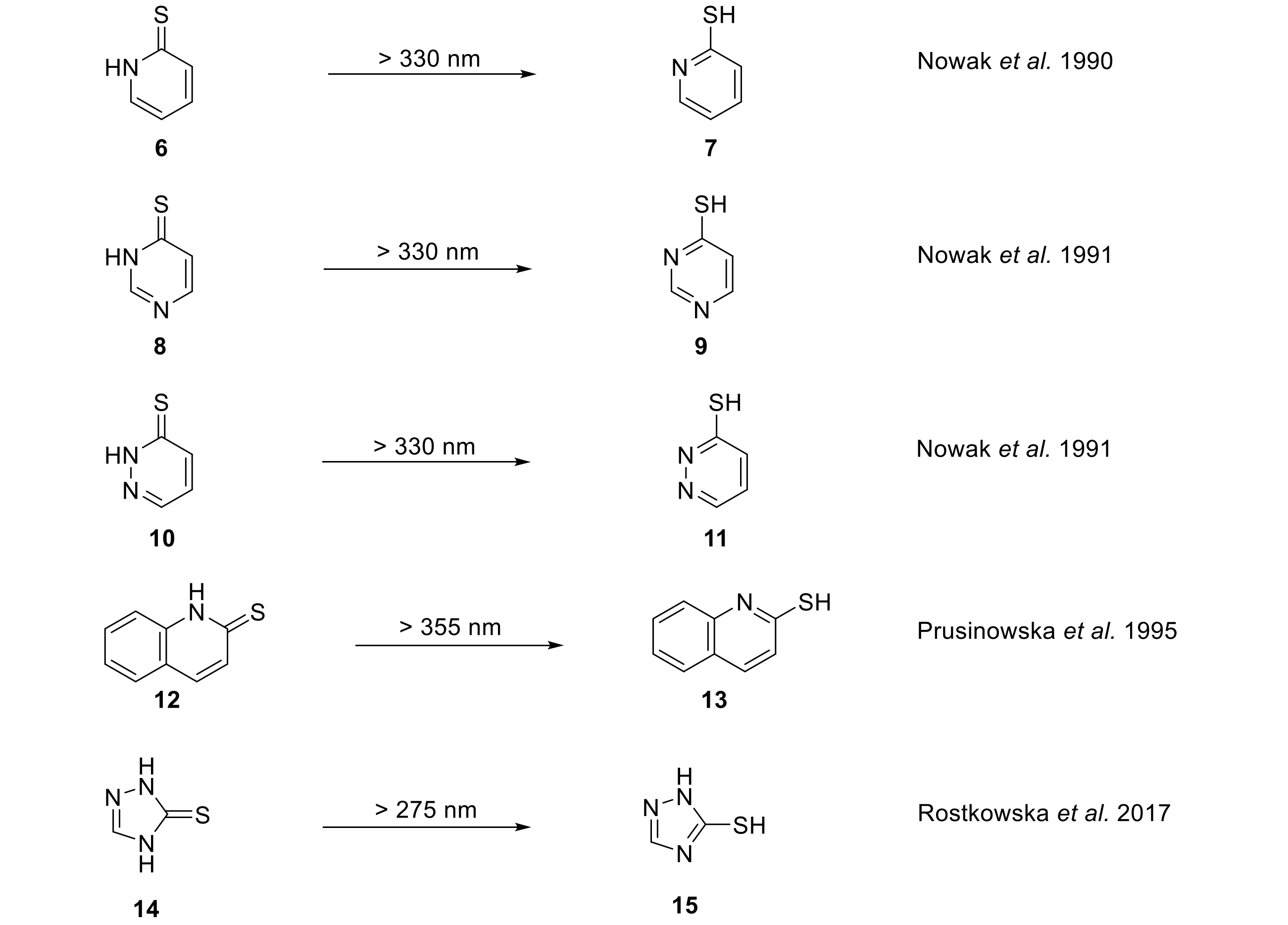

Scheme S3. Thioamide → thiolimine tautomerizations have been reported for some thioamides under matrix isolation conditions.[17–20] Note that for some of the compounds the thiolimine tautomer is lower in energy than its thioamide form due to aromaticity.

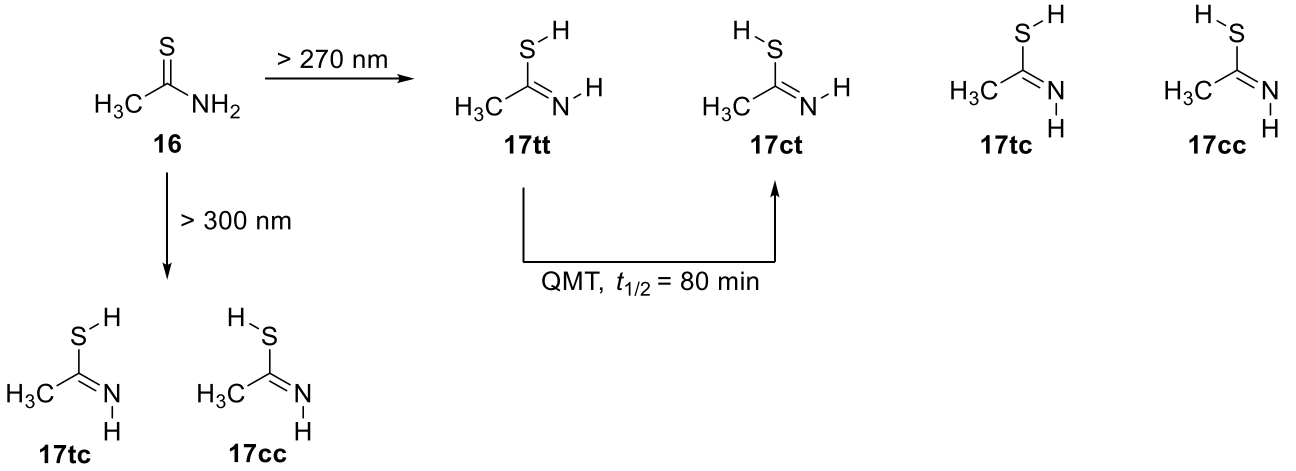

Scheme S4. Investigations on thioacetamide (**16**) under matrix isolation conditions have been reported in three studies. Lapinski *et al.* discussed the different ratios of conformers of **17** forming when applying different wavelengths.[21] Góbi *et al.* reported an rotamerization within these conformers due to quantum mechanical tunneling.[22] These authors were also able to selectively interconvert the four conformers into each other by irradiation with near-infrared light.[23]

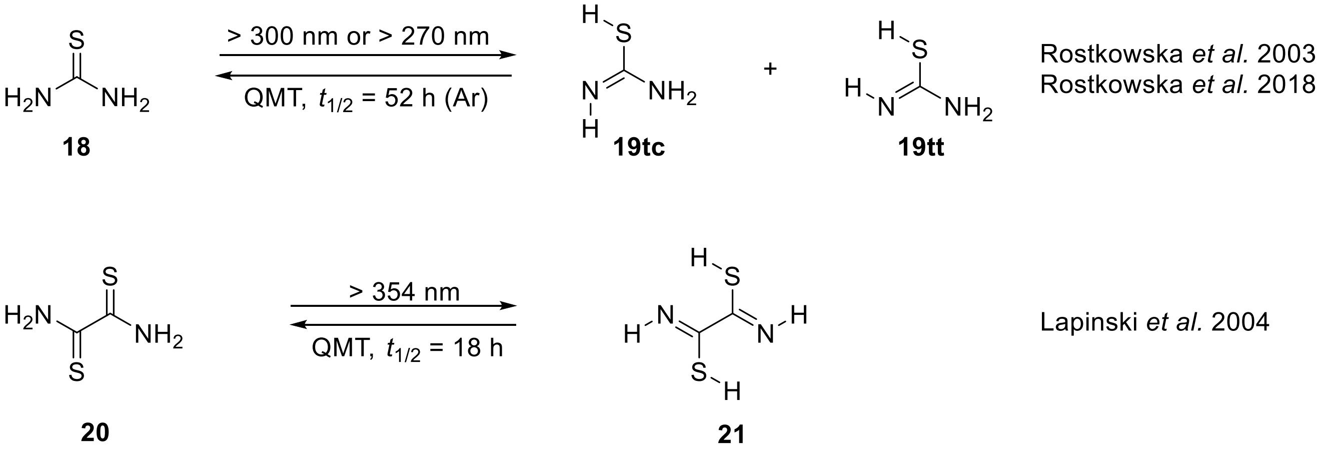

Scheme S5. Excitation of thiourea (**18**) yields two conformers of isothiourea (**19**), one of which (**19tc**) tunnels back to 18.[24,25] A similar (double proton) tunneling process is reported for dithiooxamide (**20**).[26]

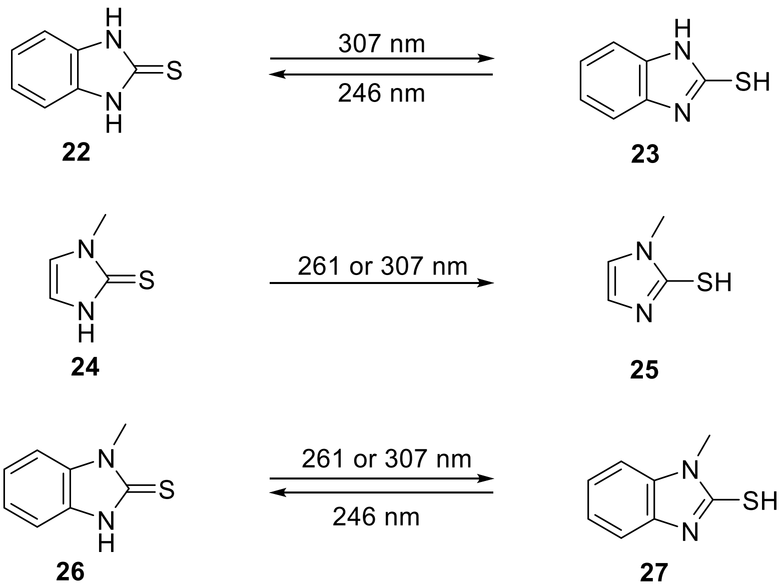

Scheme S6. Brás and Fausto discussed the reversible thioamide → thiolimine tautomerization in the three displayed compounds.[27,28]

More Isomers of Thioformamide

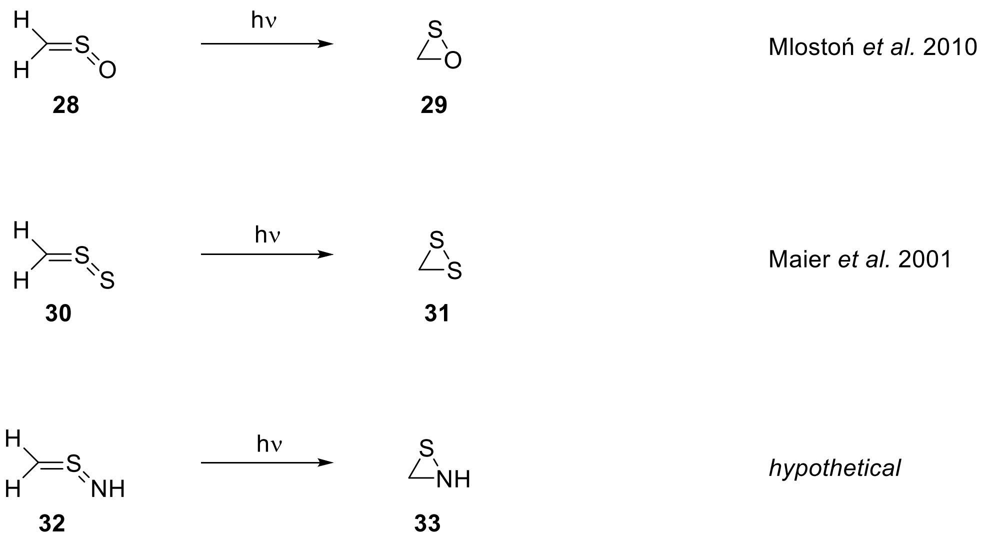

Scheme S7. Two other conceivable isomers on the $HCSNH_2$ PES are methylensulfinilimine (**32**) and thiaziridine (**33**), both of which could, however, not be detected in our experiments. Structurally similar compounds **28**, **29**, **30**, and **31** have been matrix isolated and interconverted photochemically,[29,30] At the B3LYP/6-311++G(3df,3pd) level of theory **32** is 41.9 kcal mol^{-1} and **33** is 35.2 kcal mol^{-1} higher in energy than **1**. We could not find a direct pathway from **1** to neither **32** nor **33** at this level of theory. Both of these hints presumably explain the absence of **32** and **33** in our experiments.

References

[1] R. Willstätter, T. Wirth, *Ber. Dtsch. Chem. Ges.* **1909**, *42*, 1908–1922.

[2] A. D. Becke, *J. Chem. Phys.* **1988**, *88*, 1053–1062.

[3] C. Lee, W. Yang, R. G. Parr, *Phys. Rev. B* **1988**, *37*, 785–789.

[4] Y. Zhao, D. G. Truhlar, *Theor. Chem. Acc.* **2008**, *120*, 215–241.

[5] H. Quanz, P. R. Schreiner, *J. Comput. Chem.* **2019**, *40*, 543–547.

[6] G. D. Purvis, R. J. Bartlett, *J. Chem. Phys.* **1982**, *76*, 1910–1918.

[7] K. Raghavachari, G. W. Trucks, J. A. Pople, M. Head-Gordon, *Chem. Phys. Lett.* **1989**, *157*, 479–483.

[8] T. H. Dunning, *J. Chem. Phys.* **1989**, *90*, 1007–1023.

[9] K. A. Peterson, D. E. Woon, T. H. Dunning, *J. Chem. Phys.* **1994**, *100*, 7410–7415.

[10] D. E. Woon, T. H. Dunning, *J. Chem. Phys.* **1995**, *103*, 4572–4585.

[11] R. A. Kendall, T. H. Dunning, R. J. Harrison, *J. Chem. Phys.* **1992**, *96*, 6796–6806.

[12] L. Krause, R. Herbst-Irmer, G. M. Sheldrick, D. Stalke, *J. Appl. Crystallogr.* **2015**, *48*, 3–10.

[13] G. M. Sheldrick, *Acta Crystallogr. Sect. A Found. Crystallogr.* **2015**, *71*, 3–8.

[14] S. C. Capelli, H. B. Bürgi, B. Dittrich, S. Grabowsky, D. Jayatilaka, *I. U. Cr. J.* **2014**, *1*, 361–379.

[15] O. V. Dolomanov, L. J. Bourhis, R. J. Gildea, J. A. K. Howard, H. Puschmann, *J. Appl. Crystallogr.* **2009**, *42*, 339–341.

[16] F. Weigend, R. Ahlrichs, *Phys. Chem. Chem. Phys.* **2005**, *7*, 3297–3305.

[17] M. J. Nowak, L. Lapinski, H. Rostkowska, A. Leś, L. Adamowicz, *J. Phys. Chem.* **1990**, *94*, 7406–7414.

[18] M. J. Nowak, L. Lapinski, J. Fulara, A. Leś, L. Adamowicz, *J. Phys. Chem.* **1991**, *95*, 2404–2411.

[19] D. Prusinowska, L. Lapinski, M. J. Nowak, L. Adamowicz, *Spectrochim. Acta Part A Mol. Spectrosc.* **1995**, *51*, 1809–1826.

[20] H. Rostkowska, L. Lapinski, M. J. Nowak, *J. Phys. Chem. A* **2017**, *121*, 6932–6941.

[21] L. Lapinski, H. Rostkowska, A. Khvorostov, M. J. Nowak, *Phys. Chem. Chem. Phys.* **2003**, *5*, 1524–1529.

[22] S. Góbi, C. M. Nunes, I. Reva, G. Tarczay, R. Fausto, *Phys. Chem. Chem. Phys.* **2019**, *21*, 17063–17071.

[23] S. Góbi, I. Reva, I. P. Csonka, C. M. Nunes, G. Tarczay, R. Fausto, *Phys. Chem. Chem. Phys.* **2019**, *21*, 24935–24949.

[24] H. Rostkowska, L. Lapinski, A. Khvorostov, M. J. Nowak, *J. Phys. Chem. A* **2003**, *107*, 6373–6380.

[25] H. Rostkowska, L. Lapinski, M. J. Nowak, *Phys. Chem. Chem. Phys.* **2018**, *20*, 13994–14002.

[26] L. Lapinski, H. Rostkowska, A. Khvorostov, M. Yaman, R. Fausto, M. J. Nowak, *J. Phys. Chem. A* **2004**, *108*, 5551–5558.

[27] E. M. Brás, R. Fausto, *J. Photochem. Photobiol. A Chem.* **2018**, *357*, 185–192.

[28] E. M. Brás, R. Fausto, *J. Mol. Struct.* **2018**, *1172*, 42–54.

[29] P. R. Schreiner, H. P. Reisenauer, J. Romański, G. Mlostoń, *J. Am. Chem. Soc.* **2010**, *132*, 7240–7241.

[30] G. Mlostoń, J. Romański, H. P. Reisenauer, G. Maier, *Angew. Chem. Int. Ed.* **2001**, *40*, 393–396.

Full Citations for Electronic Structure Codes

Gaussian 16:
M. J. Frisch, G. W. Trucks, H. B. Schlegel, G. E. Scuseria, M. A. Robb, J. R. Cheeseman, G. Scalmani, V. Barone, G. A. Petersson, H. Nakatsuji, X. Li, M. Caricato, A. V. Marenich, J. Bloino, B. G. Janesko, R. Gomperts, B. Mennucci, H. P. Hratchian, J. V. Ortiz, A. F. Izmaylov, J. L. Sonnenberg, D. Williams-Young, F. Ding, F. Lipparini, F. Egidi, J. Goings, B. Peng, A. Petrone, T. Henderson, D. Ranasinghe, V. G. Zakrzewski, J. Gao, N. Rega, G. Zheng, W. Liang, M. Hada, M. Ehara, K. Toyota, R. Fukuda, J. Hasegawa, M. Ishida, T. Nakajima, Y. Honda, O. Kitao, H. Nakai, T. Vreven, K. Throssell, J. A. Montgomery, Jr., J. E. Peralta, F. Ogliaro, M. J. Bearpark, J. J. Heyd, E. N. Brothers, K. N. Kudin, V. N. Staroverov, T. A. Keith, R. Kobayashi, J. Normand, K. Raghavachari, A. P. Rendell, J. C. Burant, S. S. Iyengar, J. Tomasi, M. Cossi, J. M. Millam, M. Klene, C. Adamo, R. Cammi, J. W. Ochterski, R. L. Martin, K. Morokuma, O. Farkas, J. B. Foresman, D. J. Fox, Gaussian 16 Revision C.01, **2016**, Gaussian Inc., Wallingford.

CFOUR:
CFOUR, a quantum chemical program package written by J.F. Stanton, J. Gauss, L. Cheng, M.E. Harding, D.A. Matthews, P.G. Szalay with contributions from A.A. Auer, R.J. Bartlett, U. Benedikt, C. Berger, D.E. Bernholdt, Y.J. Bomble, O. Christiansen, F. Engel, R. Faber, M. Heckert, O. Heun, M. Hilgenberg, C. Huber, T.-C. Jagau, D. Jonsson, J. Jusélius, T. Kirsch, K. Klein, W.J. Lauderdale, F. Lipparini, T. Metzroth, L.A. Mück, D.P. O'Neill, D.R. Price, E. Prochnow, C. Puzzarini, K. Ruud, F. Schiffmann, W. Schwalbach, C. Simmons, S. Stopkowicz, A. Tajti, J. Vázquez, F. Wang, J.D. Watts and the integral packages *MOLECULE* (J. Almlöf and P.R. Taylor), *PROPS* (P.R. Taylor), *ABACUS* (T. Helgaker, H.J. Aa. Jensen, P. Jørgensen, and J. Olsen), and ECP routines by A. V. Mitin and C. van Wüllen. For the current version, see http://www.cfour.de.

make_projections:
This helper tool of TUNNEX is an interface to Gaussian09/16 to facilitate the projected frequency computation from IRC forward and reverse output files. See: https://github.com/prs-group/make_projections.

Polyrate:
J. Zheng, J. L. Bao, R. Meana-Pañeda, S. Zhang, B. J. Lynch, J. C. Corchado, Y.-Y. Chuang, P. L. Fast, W.-P. Hu, Y.-P. Liu, G. C. Lynch, K. A. Nguyen, C. F. Jackels, A. Fernandez Ramos, B. A. Ellingson, V. S. Melissas, J. Villà, I. Rossi, E. L. Coitiño, J. Pu, T. V. Albu, A. Ratkiewicz, R. Steckler, B. C. Garrett, A. D. Isaacson, D. G. Truhlar, Polyrate Version 2017-C, **2017**, University of Minnesota, Minneapolis.

NBO7:
E. D. Glendening, J, K. Badenhoop, A. E. Reed, J. E. Carpenter, J. A. Bohmann, C. M. Morales, P. Karafiloglou, C. R. Landis, F. Weinhold, NBO 7.0, **2018**, Theoretical Chemistry Institute, University of Wisconsin, Madison.

HARt:
Tonto 20.01.31, github v. d26b49d https://github.com/dylan-jayatilaka/tonto.

Author Contributions

B. B. performed the matrix isolation experiments. F. D. conducted the synthesis. B. B. and A. K. E. did the computational work. J. B. performed the analysis of the single crystal X-ray structure. P. R. S. supervised the project. B. B. and P. R. S. co-wrote the manuscript.
